# Navigating new norms: a systematic review of factors for the development of effective digital tools in higher education

**DOI:** 10.1002/2211-5463.70151

**Published:** 2025-10-30

**Authors:** Akmal Arzeman, Jessica Haines, Connie Pritchard, Stephen Rutherford, Nigel Francis

**Affiliations:** ^1^ School of Journalism and Marketing Cardiff University UK; ^2^ School of Biosciences Cardiff University UK

**Keywords:** blended learning, digital resources, digital tools, higher education, learner engagement, student‐centred learning

## Abstract

The rapid shift to online and blended learning in higher education has led to the development and use of digital tools that support student engagement and learning outcomes. This systematic review examines the effectiveness of these digital tools across various disciplines in higher education, focussing on factors that promote or hinder student engagement. A criteria‐based comprehensive systematic search of three databases (Scopus, Web of Science and ProQuest, last date of enquiry 20 August 2024) identified 25 studies, inclusion criteria focussing on primary studies describing and evaluating interactive digital tools designed to enhance learning and/or assessment in higher education. Papers were analysed for bias using JBI checklists, and the papers' findings were analysed using a thematic analysis approach. Analysis of the papers uncovered four key design features that foster engagement with effective digital tools: interactivity, ease of use, immediate feedback and personalised learning experiences. Based on these findings, this review proposes a cyclic model for designing digital tools, emphasising an initial needs analysis, integration with course content, active engagement of students and educators, and ongoing refinement based on feedback. This model offers actionable guidelines for educators and institutions aiming to optimise digital tool development in higher education. The papers identified were typically short‐term studies, on specific cohorts of students, and more long‐term studies of the impact of digital resources are needed to determine long‐term learning gain. The systematic review underscores practical strategies for leveraging digital tools to promote active, self‐directed learning by focussing on evidence‐based principles.

## Graphical abstract

What factors make for an effective digital learning tool in Higher Education? This systematic review identifies elements of a digital tool that published examples reveal to be features of an engaging and impactful digital tool. A systematic literature search yielded 25 research papers for analysis. Findings reveal four key features, and an idealised design process, for impactful digital learning tools.

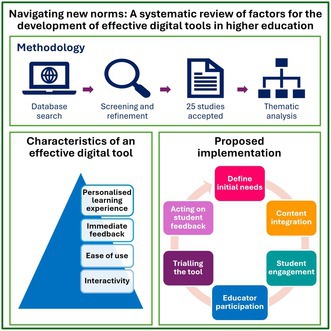

**Abbreviations**
CoAST: collaborative augmentation and simplification of text
HE: higher education
HEI: higher education institution
MATLAB: Matrix Laboratory
MOOC: massive open online course
OVL: Online Virtual Laboratory
PDA: Portable Digital Assistants
PRISMA: preferred reporting items for systematic reviews and meta‐analyses
SISTer: Student's Interactive Skull‐Base Trainer
VLP: virtual learning platform
VMAT: Virtual Microscopy Adaptive Tutorial

The COVID‐19 pandemic forced an unprecedented shift in Higher Education (HE), requiring educators to rapidly transition to remote learning. Within weeks, HE Institutions (HEIs) faced the challenge of migrating traditional, in‐person pedagogies to digital platforms [1]. This operational and existential disruption required a reimagining of established educational approaches, presenting complex challenges for educators, who had to familiarise themselves with new digital tools while redeveloping the delivery of courses within the constraints of these new modalities [2]. The continuity of students' education was dependent on educators' capabilities to rapidly adapt to the ‘new norm’, a task that was compounded by a lack of prior experience with online teaching [3].

The paradigm shift caused significant stress for both academics and students, revealing a digital divide that highlighted inequities in technology access and raised fairness concerns in digital education [4], including hardware availability and internet access. Nonetheless, the transition to online learning sparked change and new teaching practices, prompting a re‐evaluation of effective learning, teaching and assessment in higher education [5, 6, 7]. Initially, digital tools and online teaching practices were adopted out of necessity rather than choice. The rapid switch to digital platforms was often made without a clear understanding of effective pedagogy [8], resulting in this widespread drastic change in learning methods being termed by some as ‘Panic‐gogy’ [9, 10]. Various applications quickly became essential for teaching, with platforms such as Zoom, Microsoft Teams and Google Classroom integrated into learning management systems (LMS), virtual learning environments (VLE) or virtual learning platforms (VLP) such as Moodle, Canvas or Blackboard, simulating traditional classrooms virtually [11].

The shift to online platforms and the rapid adoption of more bespoke digital tools were largely reactive, focussing on immediate needs rather than on best practices or evidence for effective online learning. The choice of tools prioritised availability and ease of use over a thorough understanding of their potential and limitations [12]. This led to a varied online learning experience; some students benefited from engaging digital environments, while others encountered challenges such as reduced engagement, access issues and digital fatigue.

Before the pandemic, research into digital learning predominantly focussed on blended learning or narrow applications of online learning. The pandemic forced a shift to fully online emergency teaching [1], leaving educators navigating unfamiliar territory through trial and error. This underscored the need for evidence‐based guidelines for digital tools in teaching and assessment [13]. As the pandemic continued, higher education adapted; the initial urgency shifted to a better understanding of integrating these technologies into various disciplines.

Best practices have improved the use of digital tools, moving from emergency remote instruction to informed digital education strategies. In biomedical sciences, virtual laboratories and simulations addressed the challenges of practical instruction, allowing continuity of learning and access to complex procedures otherwise limited by traditional laboratories due to logistical constraints [14]. For example, engineering students engaged in project‐based learning using software such as Autodesk Tinkercad [15] or MATLAB [16], maintaining hands‐on education while learning remote collaboration practices common in professional fields [17]. In the humanities, digital tools enabled interactive learning through video conferencing for lectures and seminars, using breakout rooms and instant feedback, which enhanced engagement and facilitated discussion despite physical separation [18].

From all these examples, it is clear that following the enforced re‐evaluation of teaching methodologies, digital tools became enablers of engagement that facilitated the development of both discipline‐specific and key transferrable skills. As we have returned to face‐to‐face teaching, the challenge is seamlessly integrating digital tools with conventional teaching methods [19]. However, to achieve this, it is important to understand the key features that contribute to an effective digital tool. We present a systematic review of the literature to elucidate these factors.

A series of objectives underpins this systematic review:

1. to assess the digital tools used for learning and assessment across diverse disciplines in higher education, focussing on those endorsed by student feedback and engagement;
2. to explore tertiary‐level students' perceptions and experiences with digital tools through the lens of student satisfaction and engagement;
3. to determine the key features and characteristics of digital tools that correlate with positive student learning experiences; and
4. to summarise the findings into practicable recommendations for higher education institutions, centred on how digital tools and staff‐led pedagogical approaches can be optimised, implemented and integrated, based on student feedback, to support effective learning and assessment in online and blended learning environments.

This systematic review aimed to contribute to the ongoing discourse on digital education, offering evidence‐based insights to shape future practices and ensure that the rapid advancements in digital teaching and learning tools are fully leveraged to augment and enhance student learning experiences and outcomes. Student researchers spearheaded this review to provide a particularly user‐focussed view of the factors underpinning a successful digital tool.

## Methods

### Comprehensive literature searching

Three databases, Scopus, Web of Science and ProQuest, were searched systematically, using Boolean search strings (Table 1). These strings were customised to the requirements of the individual databases to match each other as closely as possible to ensure alignment between searches. The search strings were designed to identify papers that evaluated the effectiveness of the use of digital tools in higher education. The keywords were designed to identify constructs labelled as both digital tools and digital resources. For clarity and to underpin the focus on approaches that were substantive, interactive learning items, we adopt the term ‘digital tool’ throughout the review.

**Table 1:** Database search strings. Aligned search strings were used to search the three databases—Scopus, Web of Science and ProQuest—with any limits noted.

Database	Search string
Scopus	( TITLE‐ABS‐KEY ( "digital education" OR diged OR "digital teaching" OR "digital learning" OR "digital assessment" OR "blended learning" OR "virtual learning" OR "digital eLearning" OR "digital e‐learning" OR "hybrid learning" OR "mobile learning" OR "distance learning" ) AND TITLE‐ABS‐KEY ( "higher education" OR college OR "tertiary education" OR "undergraduate education" OR "postgraduate education" OR "post‐secondary education" OR university OR undergrad* OR postgrad* ) AND TITLE‐ABS‐KEY ( "educational technology" OR edtech OR "ed tech" OR "eLearning tools" ) AND TITLE‐ABS‐KEY ( effective OR impact* ) ) Limited to title, abstract and keywords
Web of Science	(((TS=("digital education" OR diged OR "digital teaching" OR "digital learning" OR "digital assessment" OR "blended learning" OR "virtual learning" OR "digital eLearning" OR "digital e‐learning" OR "hybrid learning" OR "mobile learning" OR "distance learning" )) AND TS=("higher education" OR college OR "tertiary education" OR "undergraduate education" OR "postgraduate education" OR "post‐secondary education" OR university OR undergrad* OR postgrad* )) AND TS=("educational technology" OR edtech OR "ed tech" OR "eLearning tools")) AND TS=(effective OR impact*) Limited to Topic
ProQuest	abstract("digital education" OR diged OR "digital teaching" OR "digital learning" OR "digital assessment" OR "blended learning" OR "virtual learning" OR "digital eLearning" OR "digital e‐learning" OR "hybrid learning" OR "mobile learning" OR "distance learning") AND abstract("higher education" OR college OR "tertiary education" OR "undergraduate education" OR "postgraduate education" OR "post‐secondary education" OR university OR undergrad* OR postgrad*) AND abstract("educational technology" OR edtech OR "ed tech" OR "eLearning tools") AND abstract(effective OR impact*) Limited to abstract

### Systematic refinement using inclusion and exclusion criteria

**Fig. 1:**
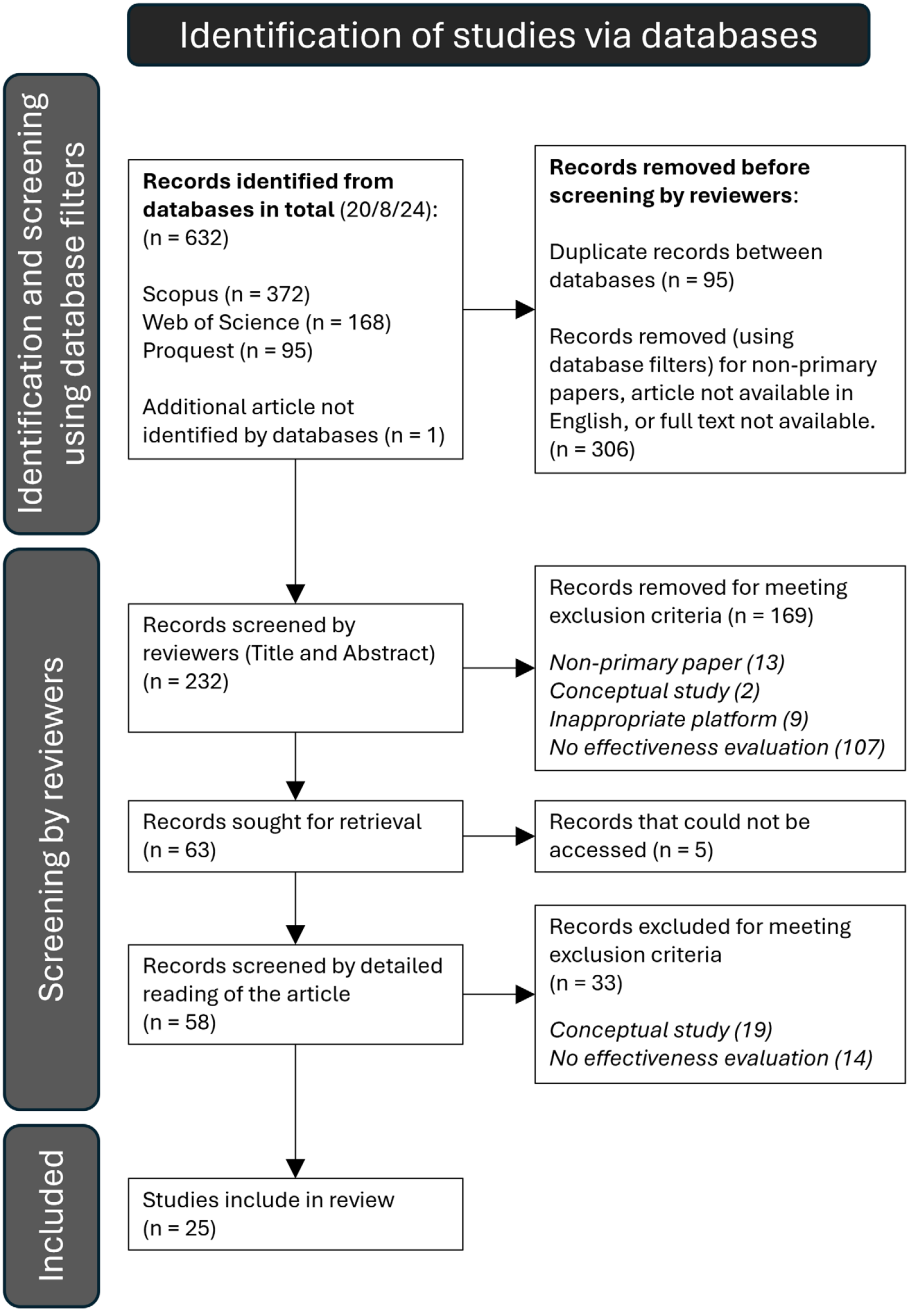
PRISMA flowchart detailing the study search and selection process applied during the systematic review. The flowchart depicts the process of identification, screening, eligibility assessment, and inclusion of studies. The initial search across three databases (Scopus, Web of Science and ProQuest) resulted in 632 records, with one additional article identified through other sources. After the removal of duplicates (*n* = 95) and using filters on the databases themselves to exclude articles based on the predefined criteria (shown in bold in Table 2; *n* = 306). A total of 232 records were screened by the review team evaluating the title and abstract, again using the exclusion criteria listed in Table 2. Full text reports were sought for 63 articles, with 58 accessible. These were assessed against the same exclusion criteria through a detailed reading of the article. Of these, 33 were excluded, resulting in 25 studies that met the inclusion criteria and were incorporated into the final analysis.

A total of 632 papers were identified through the initial screen (last date of screening 20 August 2024). Exclusion criteria (Table 2) were applied to the papers for a systematic screening process according to the PRISMA analysis protocol of Page *et al*. [20]. Numbers of records excluded and retained at each stage are summarised in a PRISMA diagram shown in Fig. 1, with a summary of the number of papers excluded for specific reasons noted in the secondary and tertiary screening stages. Primary screening was conducted using the filter features of the databases themselves to exclude papers that were not primary research papers, not available in English or not available to access. Each remaining paper was evaluated independently by the same four members of the research team against the exclusion criteria in Table 2. Initially, the title and abstract were reviewed, and then, any remaining papers were read in full and evaluated. Where differences of opinion occurred, the fifth member of the research team reviewed the paper independently to provide the final decision. After the application of exclusion criteria, the final number of papers included in the review was 25; these are listed in Table S1. Papers identified for potential inclusion were evaluated for potential bias using the JBI criteria. The ‘Checklist for quasi‐experimental studies’ [21] was used for quantitative studies. In those cases where the research was purely qualitative, the ‘Checklist for qualitative studies’ [22] was used. For mixed‐methods studies, both of these criteria sets were applied. The outputs of each of these evaluations for the 25 papers are included in Appendix S1. As this was not a healthcare‐related systematic review, the protocol was not registered or published. A PRISMA checklist is included in Appendix S2.

**Table 2:** Exclusion criteria, in order of application, used to screen initial literature search results. Criteria in bold were applied during primary screening using database filters. All of the criteria were applied in secondary and tertiary screening rounds by the review team.

Criterion
**Non‐primary paper** (e.g. review or conference proceeding)
**Article not available in English**
**Full text article not available**
No evaluation of a specific technology or tool or broad conceptual study
Study not conducted in University or Higher Education setting
No evaluation of the effectiveness of the tool or technology using qualitative or quantitative data

### Use of AI during literature search

As a final check of papers to ensure that no papers had been missed, Claude (Anthropic AI) was used to summarise the papers and give an opinion on whether they met the inclusion and exclusion criteria. No papers were included or excluded without human oversight. The final prompt used for Claude.ai is provided in Appendix S3.

### Thematic analysis of papers

The selected papers were reviewed for common features of effective and engaging online/digital tools. The detailed review of the papers was deliberately undertaken by two student researchers to encourage the analysis to be undertaken from a learner's perspective rather than an educator's perspective. This approach aimed to align any evolving framework to the needs of users of digital learning tools rather than their designers.

A thematic analysis process was adopted, adapted from the 6‐stage structure described by Naeem *et al*. [23] as follows: Step 1: Transcription and familiarisation with the data was undertaken through detailed reading of the papers by a minimum of two researchers. Step 2: Selection of keywords was interpreted as the selection of common factors that supported engagement (affordances) or inhibited engagement (barriers) through a detailed examination of the results sections of the selected papers. Step 3: Coding: this was undertaken through a detailed review of each paper and noting specific examples of data showing high or low engagement and the characteristics associated with this. These examples and factors were listed in a single shared document and agreed upon by all researchers collectively. Step 4: Theme development: the document from Stage 3 was reviewed by the two researchers from Stage 2 to identify common recurring factors. A parallel analysis process was undertaken using a review of the Stage 3 document by Claude.ai in response to a series of prompts asking the AI tool to summarise recurring factors from different perspectives (see Appendix S3). The interpretations of the AI review were then compared with the two independent human reviews to identify any elements that the human review had omitted. A final document was created to evidence the discussion of the validity of the factors identified. This review enabled a summary of generic affordances and barriers to be reported. Step 5: Conceptualisation through interpretation of keywords, codes and themes was interpreted through comparing the affordances and barriers to determine a set of stages in the design and implementation of effective digital tools. Step 6: Development of conceptual model; the final step involved creating a framework for the design priorities and a timeline of the design process.

**Fig. 2:**
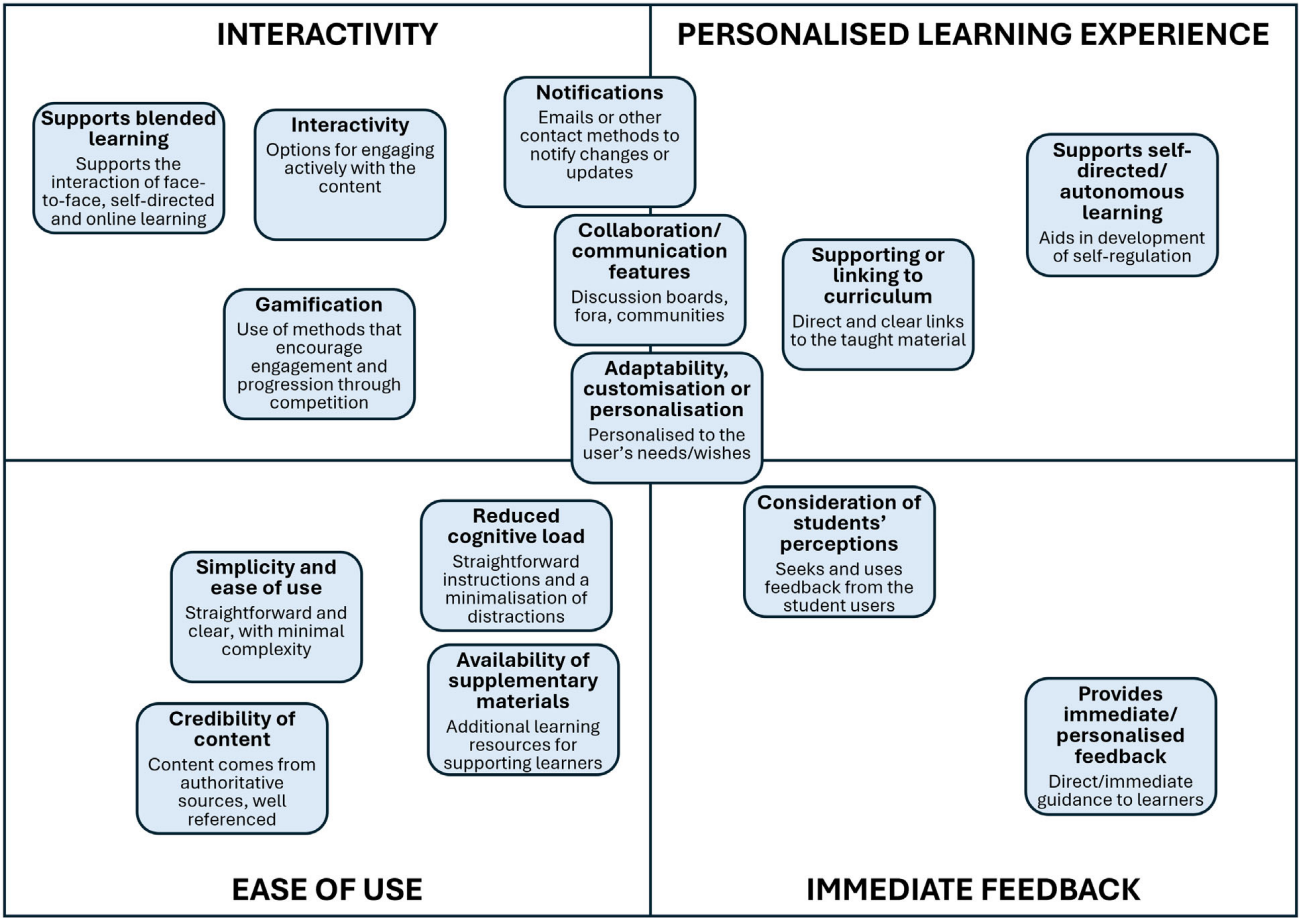
Factors contributing to an effective and engaging digital tool. Individual boxes represent factors which lead to enhanced engagement with the resource and/or increased student satisfaction. The positioning of the boxes is a nonquantitative representation of how closely the factor aligns with the four broad themes, identified in Fig. 4, of interactivity, ease of use, feedback and personalised learning experiences.

**Fig. 3:**
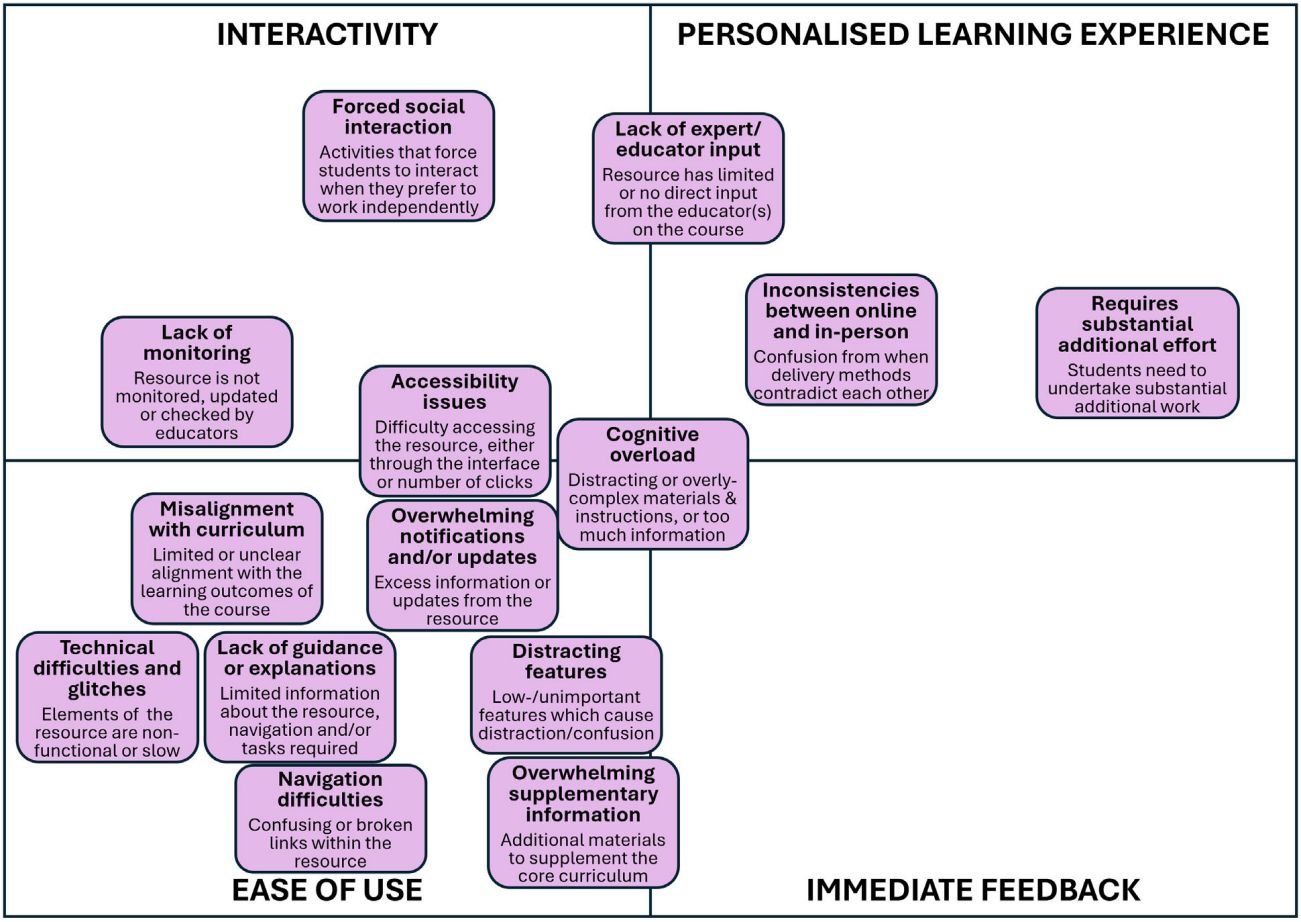
Factors that act as barriers to an effective and engaging digital tool. Individual boxes represent factors causing a lack of, or limited, engagement with the resource, and/or student dissatisfaction or frustration. Positioning of the boxes is a nonquantitative representation of how closely the factor aligns with the four broad themes, identified in Fig. 4, of interactivity, ease of use, feedback and personalised learning experiences.

**Fig. 4:**
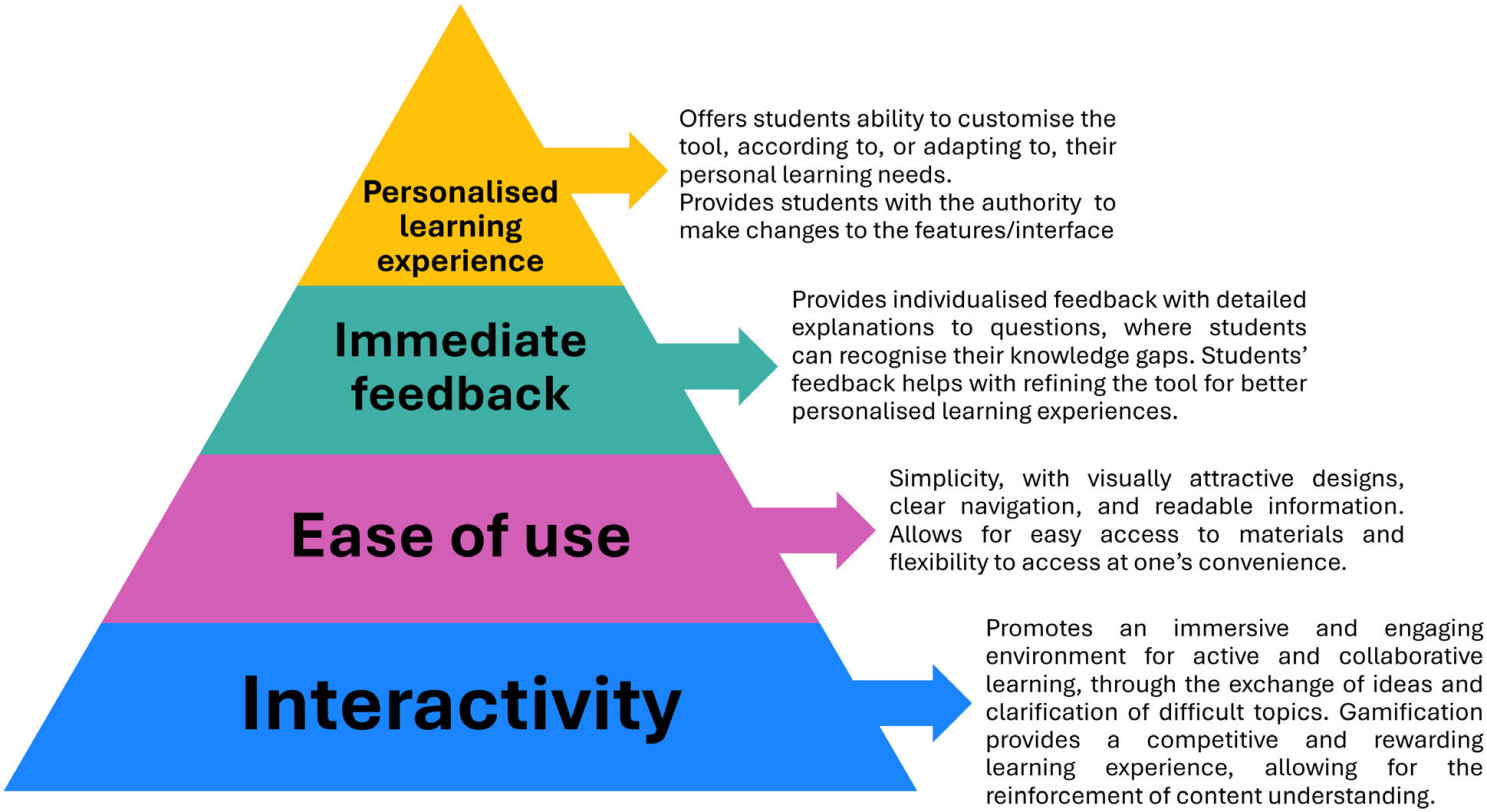
Pyramid model of core factors for designing effective digital tools in Higher Education. The figure illustrates four hierarchical levels essential for creating engaging digital learning tools. The foundation, ‘Interactivity’, supports an immersive learning environment that promotes active engagement and collaboration. The next level, ‘Ease of Use’, ensures simplicity in design for ease of navigation and accessibility. ‘Immediate Feedback’ provides real‐time, individualised responses to foster understanding and guide student learning. The pinnacle, ‘Personalised Learning Experience’, empowers students to customise the tool to their learning needs, enhancing autonomy and overall user experience. Each level contributes to building an effective, student‐centred digital tool.

**Fig. 5:**
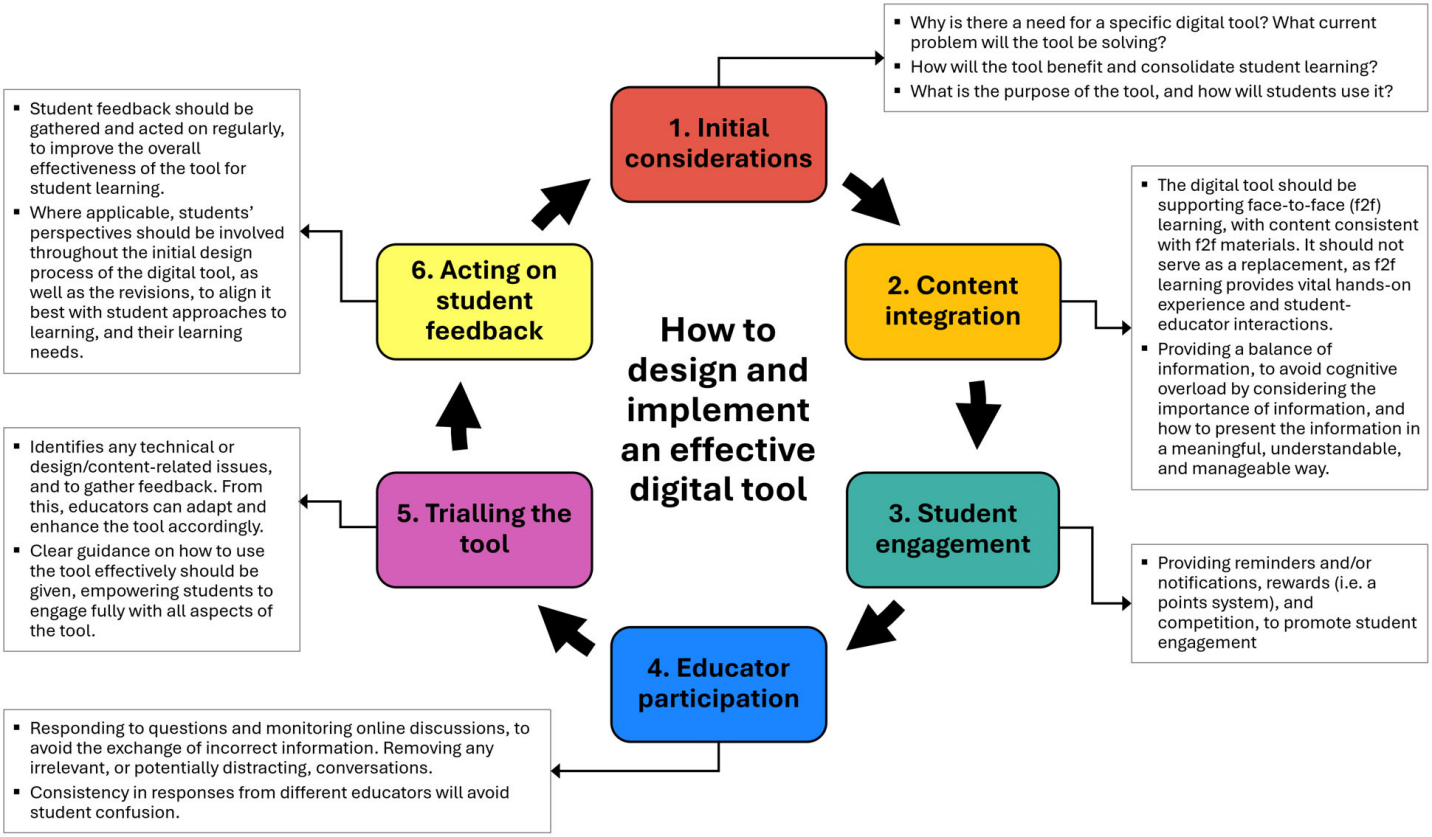
Cyclical process for designing and implementing effective digital tools in Higher Education. This figure illustrates a six‐step cyclical framework that guides the development of digital tools aimed at enhancing student engagement and learning outcomes. The process begins with ‘Initial Considerations’, where the need for the tool and its intended benefits are assessed. ‘Content Integration’ ensures the tool complements face‐to‐face learning and avoids cognitive overload. ‘Student Engagement’ strategies are devised to motivate and involve learners actively. ‘Lecturer Participation’ highlights the role of educators in maintaining relevance and accuracy within the tool's usage. ‘Trialling the Tool’ allows for the identification of technical and design issues and ensures clear guidance is provided. Finally, ‘Acting on Student Feedback’ involves using gathered insights to refine and improve the tool. The cyclical nature emphasises continuous improvement, reinforcing the tool's alignment with educational goals and user needs.

The findings of the thematic analysis were purely qualitative, and as a result were not quantifiable. The findings were synthesised into factor maps (Figs 2 and 3), a hierarchy of requirements (Fig. 4) and a development cycle (Fig. 5). Ordering and positioning of elements within these figures are relative interpretations, based on a combination of prevalence and reported importance within the source material, relative impact of the factor on the design and efficacy of the tool and relative impact of the factor on student engagement with the tool. The resulting figures are therefore representational and do not imply any specific numerical quantity of any factors.

## Results

### Summary of digital resources identified from the scoping review

The 25 papers identified covered a range of digital resource types, ranging from platforms and learning environments to specific tools and interactive simulations. Table 3 summarises the tools in broad categories. As stated in the Methods section, we defined a ‘digital tool’ as an online resource that performed a specific means of delivering or reinforcing content to students whose impact could be evaluated either by engagement levels or by the impact on student outcomes. Typically, VLE or content systems were excluded unless used interactively or having measurable impact.

**Table 3:** Digital tools included in this review. Some papers selected from the literature search involved more than one tool, and hence are included more than once in the table.

Digital tool	Features	References
Online discussion boards	Promotes discussion and exchange of ideas between students. Provides formative feedback.	[26, 32, 34]
Virtual learning environment	e.g. Moodle. Easily accessible modules and supporting content (e.g. videos) with interactive elements. May include adaptive‐mediated instruction to provide personalised learning paths or allow the students to personalise their own platform. May also include discussion forums and support feedback between student and educator. Can be aligned with principles of gamification.	[27, 28, 31, 32, 37, 39, 47, 56, 57]
Text simplification tool	Supports engagement and understanding of complex texts.	[41]
Polling tool	Online polling tool can contribute to active learning.	[57]
Online quiz	Promotes revision of material and self‐evaluation.	[24, 30]
Educational online games or gamified learning environment	e.g. Escapp escape room with team‐based quiz Qs and puzzles, leaderboards and time limitations.	[24, 39, 40]
Computer programming platform	e.g. Wampserver allows students to develop computer programming techniques.	[58]
Collaborative learning tool	Utilised in problem‐based learning. Supports students to identify group challenges and develop strategies to overcome them.	[33]
Reinforcement learning tool	e.g. YouTube channels, iBook, MOOC, podcasts to support learning. Portable tools such as mobile phones or portable digital assistants can be used to access other online tools, keep notes, collaborate and receive regular, bite‐sized information.	[35, 45, 50, 59]
Consolidatory learning tools	e.g. Interactive video lectures and massive online open courses (MOOC).	[29, 59]
Interactive anatomical training tool	e.g. Student's Interactive Skull‐Base Trainer (SISTer). Interactive, multi‐media‐based content, quiz questions with immediate feedback, 3D models, examples of diagnostic findings.	[48]
Virtual adaptive medical tutorials	Interactive tool including questions with immediate feedback, educational images and videos. User personalisation can be tailored to level of knowledge.	[36]
Laboratory simulation	Interactive simulation of laboratory techniques with ability to design experiment, run it and interpret the data. Includes multiple‐choice questions and drag and drop exercises.	[25]

### Characteristics of an effective digital tool

A thematic analysis of 25 papers identified a range of common factors as either affordances, contributing to an effective tool (Fig. 2), or barriers, reducing student engagement (Fig. 3). These factors align with four development stages, which were subsequently identified as key for creating an effective digital tool. The positioning of the factors in Figs 2 and 3 within these four themes indicates which of these themes they were most strongly aligned with.

Engagement can be enhanced through interactivity—features that promote active participation, such as simulations, games, quizzes, adaptive activities and interactive videos. Collaboration and/or communication features—tools for online discussions, shared annotations and peer assessments facilitate collective intelligence and peer learning. Gamification—elements such as challenges, targets and progressive achievement levels foster a challenge mentality, motivating students to achieve and improve.

Utility and ease of use were key for engagement. Simplicity was vital for a user‐friendly tool that was easy to navigate; complex tools often caused disengagement. Effective features included intuitive, visually appealing interfaces with straightforward navigation. Frequent targeted notifications about updates and deadlines aided student learning. Reducing distractions minimised cognitive load, aiding their learning. Designing features with user experience in mind was ideal. Cocreation of tools with students in the design process was rare but beneficial where adopted (e.g., in Nuci *et al*. [24] and Francis *et al*. [25]).

Other factors emphasised personalisation and how the tool could be tailored to individual students' needs. Adaptability allowed for customised tools and content, fostering student engagement through personalised learning paths. Adaptive tutorials and reminders enabled this. Features that encouraged self‐directed learning, helping students explore subjects independently, were seen as beneficial. Immediate feedback from automated grading and progress tracking helped identify knowledge gaps and improve outcomes.

Features enabling the digital tool to mirror or reinforce core content improved trust and engagement. Alignment with the curriculum—tools linked to learning outcomes engaged students, demonstrating clear benefits. Credibility—validation by subject experts enhanced student trust and engagement. Blended learning—combining digital tools with traditional methods fostered a cohesive approach; however, alignment of delivery modes was essential (see below). Supplementary resources—providing study materials and guides connected online and in‐class content, enhancing user experience and deep learning.

Many features were identified as barriers to effective engagement (Fig. 3). Notably, barriers centred on ‘ease of use’, indicating that direct interaction with the tool posed the most significant challenge for students. Distracting elements and navigation difficulties discouraged students from exploring the tool. Additionally, a lack of guidance and explanations frustrated students, complicating their user experience. This closely aligns with frustrations from technical difficulties and accessibility issues, such as the need for complicated authentication to access the tool. Surprisingly, the papers reviewed did not highlight accessibility concerns for specific learning support needs, disabilities or neurodiversities.

Accessibility features also strongly align with barriers through excessive cognitive load on students, through distractions, irrelevant information or complex interfaces. This was closely aligned with student concerns over overwhelming numbers of notifications and/or updates relating to revisions to the tool, errata or additional *ad hoc* guidance. Finally, if the tool required substantial extra effort on the part of the student, they were unlikely to want to engage with the additional workload.

Other barriers related to students being able to see clear alignments between the digital tool and their core curriculum content. Therefore, inconsistencies between online and in‐person content were potentially confusing and discouraging for the students. General misalignment with the curriculum was a potential problem, as it would prohibit students from seeing a purpose in using the tool.

Peer–peer and peer–educator interaction was also a potential barrier if handled wrongly. Forced social interaction between students was sometimes reported as problematic if it forced students to engage with each other when they would have preferred to be autonomous learners. Student–educator barriers were where there was either a lack of expert/educator input in discussions or online interactions and in some cases, a lack of monitoring or moderation that led to students feeling disengaged from their educator and unsupported.

An overview of the factors associated with the design and implementation of these tools identified factors that aligned with successful engagement and/or impact. The overview also inferred a series of key stages in a digital tool's design and development process. This review of the features led to the production of a series of ‘design considerations’ for effective online tools.

### A model for the design features of an effective digital tool

Analysis of the 25 papers identified in the systematic review defined key factors of effective digital tools. These factors were synthesised into four core themes: interactivity, ease of use, immediate feedback and personalised learning experience (Fig. 4). Based on the relative reported impact on the utility of the tools, and the apparent impact of the design on student engagement, we propose a qualitative hierarchy of importance to these factors. The hierarchy is based on the importance of one factor in underpinning the needs of another and has been inferred from an overview of all the platforms. Interactivity emerged as the most fundamental factor, as it underpinned the efficacy of all of the platforms. Subsequent layers reflect less ubiquitous elements due to increased specialisation and individualisation of the tools, making development more challenging and limiting their adoption. Based on their apparent impact on other factors, we have proposed these in an ascending hierarchy of importance. Conversely, however, it was noted that these less‐ubiquitous features appeared to be of increasing impact on learning.

#### Interactivity

The most heavily represented factor, by far, for a successful digital tool was the extent of interactivity between the tool and the user. This has, therefore, been suggested as the foundation for all design considerations.

Interactive tools encouraged students' active participation in learning, featuring activities that required activity and cognitive engagement to improve understanding and enhance experiences. Examples include interactive videos, gamified activities, simulations, quizzes, discussion forums, Portable Digital Assistants (PDAs), Virtual Microscopy Adaptive Tutorials (VMATs), Kahoot and Google Form Quiz and VLPs such as Moodle.

James *et al*. [26] outlined the two‐way interactions within their tool, facilitating two‐way interactions by enabling virtual exchanges and allowing students to connect. This interpersonal interaction enhances engagement, particularly for those lacking physical interaction, as students contribute new ideas and feel part of a community. Pereira *et al*. [27], Francis *et al*. [25] and Deperlioglu and Kose [28] also had platforms that promote peer information sharing. Hung and Chen [29] noted that discussion forums foster ‘collective intelligence’, empowering students to share knowledge and ideas. Evans *et al*. [30] highlighted the impact of this interaction, significantly boosting students' engagement with course materials and each other. Yang *et al*. [31] observed that online discussions developed higher levels of interaction, leading to deeper learning.

Evenhouse *et al*. [32] observed that online forums bridged social boundaries, allowing students to connect and collaborate without prior personal connections. This connectivity enabled them to ask questions anonymously, resolving issues without losing face or sharing personal contact information. In contrast, the approach adopted by Lyons *et al*. [33] required students to work in groups within a nonthreatening online environment. This method was more socially acceptable, especially for shy or introverted students, reducing awkwardness and providing a ‘safe space’ for interactions. Students could view and thoughtfully respond to each other's opinions, enhancing group dynamics. Student feedback on discussion fora in Shana [34] indicated that they encouraged new perspectives and idea generation by collectively reading diverse responses. Forum discussions prepared students for collaboration in class through idea exchange and peer assessment. Online discussions boosted participation, enabling reserved students to gain confidence and ensuring all had a chance to engage. Ultimately, discussion fora fostered intellectual interactions and connections, broadening opportunities for shared knowledge and reflection.

Hung and Chen [29] and Shana [34] highlighted opportunities for student‐educator interactions. This was also emphasized by Reynolds *et al*. [35], whose tool provided interactive features, such as class voting, online questionnaires, and self‐assessment quizzes for students and lecturers to enjoy. Shana [34] noted that these tools not only enabled interaction between students and teachers but also produced students who engaged actively in learning and collaboration.

Van Es *et al*. [36] noted that interactive opportunities enhanced the quantity and quality of engagement with the VLP. Ahmed and Hasegawa [37] echoed this observation, observing that interactive learning content enhanced the effectiveness of the VLP.

Integrating fun elements boosts student engagement. Gamification, adding self‐competitive aspects to learning, enhances engagement [38]. Estriégana *et al*. [39] linked gamification's recreational benefits to its educational uses. They proposed including educational games for improved engagement. Nuci *et al*. [24] noted that gamification increases student engagement and interaction in lectures, prompting the incorporation of these elements into their tool. Gamified elements in quizzes improved student engagement and enhanced the user experience.

Gamification was taken to its full potential by Lopez‐Pernas *et al*. [40] in their ‘Escapp Room’. The online, open‐source Escapp client library (*ad hoc* software used to create the Escapp rooms) allowed students to communicate with the Escapp platform for clues or assistance. This stepwise information release synchronised students' progress as a team and provided relevant updates to help them advance in the task. With synchronisation, students could collaboratively solve Escapp room puzzles remotely.

#### Ease of use

Multiple papers specifically emphasised ‘ease of use’ as fundamentally important to the success of a digital tool [27, 28, 35, 40, 41], while others implied this through their design choices. Ease of use and simplicity of the interface are, therefore, important considerations once the overall interactivity design of the tool has been determined.

Ease of use lowers engagement barriers. Cognitive load is a key challenge in any learning activity [42, 43], especially with new activities. Elements that minimise ‘Extrinsic Load’ (the complexity of the instruction presented to a learner) and ‘Extraneous Load’ (the complexity of format and/or visual distractions) help reduce frustration and enhance user experience [44]. Today's students expect instant access to online media, making it crucial to streamline access to materials for effective engagement. The reviewed papers highlighted ease of use through a self‐explanatory interface, easy navigation, clear and attractive visuals, flexibility and usability on multiple forms of devices.

An intuitive, self‐explanatory interface was essential for Pereira *et al*. [27] and Reynolds *et al*. [35]. The tools were self‐explanatory and straightforward to learn to use, allowing users to quickly find their desired information. This ease of use and navigation was also crucial for Shardlow *et al*. [41], where users valued quick access to information. Annotation features helped users understand words and phrases, saving time and reducing frustration [33]. Ahmed and Hasegawa [37] embedded their tool within a familiar VLP, offering guidance for effective access. They provided theoretical explanations and videos covering essential skills for designing and producing an ‘Online Virtual Laboratory’ (OVL). The ‘Escapp Room’ of Lopez‐Pernas *et al*. [40] included clear instructions to assist users in navigating the platform.

A visually appealing and attractive tool helps smooth the ease of use. If an activity is visually engaging, the user will likely want to persist in its use. This was a priority for Lopez‐Pernas *et al*. [40] as well as Ahmed and Hasegawa [37].

Flexibility was key, especially for accessing tools via various media (computers, tablets and smartphones). Deperlioglu and Kose [28] designed a tool that allowed students to communicate asynchronously with teachers. Flexibility to facilitate easy access to peer collaboration was vital to success for Evenhouse *et al*. [32]. While the time of access was crucial for Shana [34], as the tool provided instant help and allowed students to access materials anytime, enabling frequent revisits. Ibtissam *et al*. [45] introduced flexibility, allowing students to rewatch podcasts at their convenience, noting the strong potential to consolidate learning.

Ease of use for the educator is also essential. Ahmed and Hasegawa [37] provided ready‐made templates for their VLP, providing training on how to produce OVL products by using an OVL creator tool. It was, therefore, straightforward to navigate, design, produce and publish the OVLs using the VLP, reducing staff workload and cognitive load and providing more time to perfect the content.

Reducing barriers to use is key to enhancing engagement. With many competing demands on student (and staff) time, minimising effort that could be perceived as wasteful time is essential. Instant impact and gratification lead to persistence and greater learning gain. Reducing the extrinsic cognitive load by simplifying and supporting access and by making it a pleasurable experience visually enhances the impact.

#### Immediate feedback

Engaging users also involves providing immediate feedback and guidance while they use the tool, especially regarding discussion boards [26], quizzes and self‐tests [24]. Feedback was delivered through various means, including comments on discussion board posts [26], immediate feedback on quiz questions using apps such as Kahoot or Google Forms [24], or multiple attempts for students to achieve the correct answer, employing an assessment as learning approach [25].

Timely formative feedback through task‐oriented activities helps students gauge their understanding. The closer to an activity that feedback occurs, the higher the impact on the learning gain [46]. Shana [34] and Yang *et al*. [31] enabled a facility for immediate feedback to close the learning loop with the users for maximum learning gain. Feedback was provided via interactive online discussion boards [34] or online group activities facilitated via the VLP (Moodle) [31, 36]. Van Es *et al*. [36] also embedded instant feedback into their tool, noting that it positively impacted student performance.

The role of the educator is important in this process. If the feedback is not automated, then the educator needs to be responsive to student engagement. For example, Ahmed and Hasegawa [37] provided real‐time feedback on students' performance. Mayo‐Cubero [47] was able to provide feedback to students on their written work, indicating areas or aspects that needed improvement through annotations and underlining, allowing students to track their progress and improvement. Through this, the authors aimed to replicate ‘real’ practices as closely as possible.

Evenhouse *et al*. [32] noted the importance of having someone perceived as a reliable source of information, either a teaching assistant or professor, providing helpful or correct information to students. This is especially important if there is a dispute in the online discussion's comment section. However, James *et al*. [26] enabled students to make a comparison to other students' answers to enhance their understanding. These authors also enabled a ‘question‐and‐answer’ feature, offering students guidance on where to search for answers.

With this factor, immediacy or real‐time (or as close to it as possible) engagement was crucial to provide that sense of interactivity and direct engagement. This all led towards the focal element of the tools, which was the development of a ‘personalised learning experience’.

#### Personalised learning experience

The focal aim for many tools was to enable students to customise the tools to suit their own educational needs and learning approaches. Building these features into a tool effectively assisted students in engaging more effectively and improved learning.

Ahmed and Hasegawa [37] provided training about designing Online Virtual Laboratories from preformed templates and the skills required to produce the OVL with the OVL creator tool. They also provided specialised tools for creating OVLs in diverse domains. Mayo‐Cubero [47] directed the personalised experience via the use of individualised comments for students, although that required substantial input from the educator. The ‘Student's Interactive Skull‐Base Trainer’ (SISTer) tool allowed for self‐directed learning in which students could identify their own knowledge gaps [48]. Yang *et al*. [31] adopted a more automated approach, using adaptive learning to enable the tool to tailor itself to the user's needs.

The relative paucity of these personalised learning experiences shows the difficulty of incorporating them into a tool without substantial coding and development costs. However, a personalised experience is highly important in any online activity—from shopping to gaming. So, there are clear parallels between these activities and the learning experience of a digital tool evidenced by these papers [49].

### Proposed development cycle of digital tools

The review identified an idealised process for developing and delivering effective online tools. Summarised in Fig. 5, this cyclical process—developed from a broad comparison of the development and review processes reported in the papers—illustrates that these tools need constant revision and refinement, with six crucial steps. The cyclical framework is based on both positive and negative feedback from students, along with recorded suggestions for improvement reported in the papers. The framework outlines key factors and actions for educators in designing digital tools. The analysis utilised Claude.ai to identify negative themes and factors to avoid when designing digital tools, as well as suggestions for improvements in the outputs from the collected articles. Considerations were organised logically based on cause and effect from the identified requirements. This model is derived from a synthesis of the reported factors into a logical sequence, and this cycle was not suggested in any of the papers.

#### Initial planning and needs analysis

When implementing a digital tool, the first stage is to reflect on what the intended educational objectives for the learning activity are, why a specific digital tool is necessary, and the objective/problem it addresses. The evidence for this stage was based on the different ways in which the tools were used. Some tools were more generalised enhancements of learning objectives (e.g. discussion boards and quizzes) and applicable across diverse subject areas. Other tools were specifically designed for a specific subject or topic; for example, SISTer [48] was designed to enhance the otorhinolaryngology (diseases of the ear, nose and throat) knowledge of medical students, and the Virtual Flow Cytometer [25] virtually replicates a flow cytometry practical for immunology students. Each addressed either a general need or a specific knowledge gap or requirement. When designing and implementing a digital tool, it is crucial to align it with the intended learning objectives, and to consider the problem it addresses, whether this is specific to a subject area that students struggle with or a more general tool applicable across various subjects or applications.

Another key consideration is to assess how the tool will benefit and consolidate student learning. After establishing the need for a digital tool, educators can explore the advantages and benefits for students by incorporating the tool into their learning. This links to the final question: how will students use the tool, for what purpose, and when? However, this may not align with actual student behaviour. Shana [34] incorporated a web discussion forum in their digital tool. However, students who did not favour online discussion fora expressed concerns about time commitment and the impact of distracting discussions, perceived as irrelevant to the content material. The students' use of the tool for purposes other than learning, discouraged students from using it for its intended purpose. As such, when designing a tool, it is important to consider how students might use these tools inappropriately and how to monitor and maintain their relevance to the content material.

#### Content integration

As with any learning activity, a digital tool should be designed around the content being delivered so that it best suits the subject matter and/or skills being supported by the tool. The digital tool should support face‐to‐face learning with consistent content but should not serve as a replacement for face‐to‐face opportunities, as in‐person provides vital hands‐on experience and student–educator interactions.

Von Sass *et al*. [48] designed ‘SISTer’ to integrate with the curriculum in clearly identifiable ways. As a result, 80% positive student feedback highlighted that SISTer blended well into the existing curriculum, and 90% of students said that they were able to identify gaps in knowledge through its use. This positive feedback was a consequence of the tool's integration into the curriculum. Zhang *et al*. [50], on their text message system, reported student feedback that the text alone was insufficient to learn vocabulary. Students said that reading the vocabulary in the text message did not aid memorisation. They needed to write the words down to memorise and keep track of the words. This example illustrates how digital tools should not replace traditional teaching methods but should be used to supplement them, as students still find traditional teaching methods useful. Finally, Evenhouse *et al*. [32] observed that inconsistency between the videos on the tool and the content learned in class caused some confusion for students. Therefore, it needs to be ensured that the digital tool properly aligns with the material taught in class.

Consideration also needs to be given to ensuring a balanced amount of information to avoid cognitive overload by considering the importance of the information and how to present it in a meaningful, understandable and manageable way. Zhang *et al*. [50] divided vocabulary deliveries into chunks of five words sent out daily. This broke up the information so as not to overload students. Shardlow *et al*. [41] used their platform ‘CoAST’ to show definitions of keywords. However, students were concerned by the size of the font and length of the text. It was difficult to read text on a small screen (a particular problem for students with disabilities affecting the written word), leading to students suggesting that definition lengths should be standardised. Consistency and accessibility of content are important considerations.

#### Strategy for engaging students in an online space

The next consideration is how to ensure and enhance student engagement with the tool. In most cases, a digital tool would be a formative activity in addition to the core curriculum activities and classes. Therefore, encouragement needs to be proffered to ensure the students engage with it. Providing reminders, notifications, rewards (e.g. a point system) and competition (through gamification) promotes student engagement. Zhang *et al*. [50] used text message notifications to learn vocabulary. Notifications acted as a reminder/motivator for students to learn the vocabulary rather than relying on self‐motivation.

Tools that have some sort of interactive feature, for example simulations, quizzes, promote student engagement. Nuci *et al*. [24] used Kahoot and Google form quizzes. These authors reported a significant increase in students' engagement and interaction levels in lectures where quizzes were used (Student's *t*‐test was used with a significance level of *P* < 0.05).

Personalisation was also a consideration. The ‘Collabucate’ web‐based tool [33] was designed to aid students in overcoming challenges when working in a team. Student suggestions to improve this tool generally consisted of having the ability to write their own challenges and to have adaptive challenge prompts as some students grew tired of the same questions/prompts. Allowing students to have some aspect of control or personalisation of the tool content could improve overall student engagement with the tool. The use of online discussion boards by James *et al*. [26] promoted engagement by catering to different styles of learners. Anonymous posting empowered students to share ideas without fear of judgement. With recent developments in Artificial Intelligence platforms, the potential for adapting these affordances and large language models in particular, to enhance personalisation may be increasingly possible.

#### Educator engagement, and preparation for student‐staff‐tool interaction

After the engagement strategy is determined, the tool can be designed. However, a critical step at the start of this process is ensuring correct alignment with the other taught materials, so engaging with other educators on the course is essential. Consistency in response types and timings from different educators will avoid student confusion. Equally if the digital tool involves active interaction by the educators (such as responding to questions, monitoring online discussions to avoid the exchange of incorrect information, and removing any irrelevant, potentially distracting, conversations), then buy‐in from all the educators on the associated course is needed. For example, student feedback on the online discussion boards used by James *et al*. [26] suggested students wanted lecturer participation to moderate the sharing of information to prevent the sharing of personal information and keep the content of discussions focussed. Some students also said they would have participated more if the lecturer had kept discussions on track. Therefore, educator participation could potentially motivate students to engage with the digital tool. Similar feedback was received by Shana [34], noting that students were discouraged from engaging with discussion fora, where irrelevant material was being shared. Feedback suggested that educator participation in online discussions would minimise distracting irrelevant discussions. However, this needs to be balanced against the presence of educators and ‘experts’ in discussion fora making students more passive and less likely to volunteer answers that they felt an expert would be better placed to provide [51].

#### Trialling the tool

To create a smooth and effective engagement, trialling the digital tool before general release to students is essential. This process will first ensure that the tool will not only fulfil its intended purpose but also identify any technical or design/content‐related issues, and by gathering both student and educator feedback, the tool can be adapted accordingly. Francis *et al*. [25] gained valuable trial feedback on their Virtual flow cytometer, with feedback showing that some students experienced technical difficulties when using this tool for the first time. Nuci *et al*. [24], trialling their Kahoot and Google Form quizzes, identified technical issues with the Google Form quiz. Trailing the tool allows for the identification of technical difficulties unforeseen by the designers and permits students and lecturers to become equipped with how to deal with these.

Trialling the tool will also enable the authoring of clear guidance on how to use the tool effectively, allowing students to fully engage with all aspects of the tool. Gaining student perspectives from questions asked during the trialling of the tool forestalls problems if these can be incorporated into guidance. Shana [34] provided guidance to students on how to get the most out of the tool based on student feedback. This could be applied to a range of digital tools and would be particularly useful when implementing a novel tool or a tool that is less familiar to students.

#### Acting on student feedback

Once the tool has been used with students and educators (either on a trial basis or as an educational activity), student and educator feedback should be gathered and acted on regularly to improve the overall effectiveness of the tool for student learning. Where applicable, students' and educators' perspectives, and any platform‐related usage metrics, should be involved throughout the initial design process of the digital tool intended to benefit student learning. The majority of articles gathered feedback from students using tools (via questionnaires, surveys and interviews). However, there was a lack of articles providing evidence that these suggestions were considered to improve the tool and that the tool was then retested to see if these improvements positively impacted student learning.

## Discussion

This systematic review aimed to highlight the key factors in implementing a successful and engaging online tool in Higher Education. Since digital tools and online resources are, by default, self‐directed, supporting student engagement and independence is essential. The key themes focus on leveraging interactivity, collaboration, customisation and feedback while maintaining simplicity and credibility to actively engage students and enhance learning outcomes. Avoiding cognitive overload and minimising technical issues is critical, as is consideration of inclusivity for students with differing educational support needs. In summary, effectiveness is centred on student experience and enabling active learning tailored to individuals' needs and preferences.

Experiences from the literature suggest that functionality and interactivity are the baseline requirements, with ease of use also fundamental. This aligns with any standard effective pedagogic practice, where clarity and simplicity are most often the primary concerns for student engagement. All educational resources and practices need to encourage student interaction and engagement and be effective at communicating ideas. More bespoke elements, such as immediate feedback and personalisation, are desirable but cannot be prioritised over that basic functionality. The design of digital tools, therefore, needs to consider the fundamental learning aims the tool is trying to address.

These guidelines align with several guidelines for effective online teaching developed and shared during the COVID‐19 pandemic and lockdowns—keeping the design simple, aligned to the learning outcomes and focussing on learning rather than technological affordances. For example, Mandernach *et al*. [52] highlighted that online learning needs the learner experience as its core, starting with basic factors of behavioural, cognitive, social and emotional considerations. In a scoping review of online learning, Kavashev [53] highlighted the importance of ‘heutagogical’ learning (student‐centred learning, emphasising the development of independence, self‐reliance and autonomy). Kavashev [53] observed that the most significant factor in effective online learning was collaboration and that interactivity supported the development of self‐regulated learners. A meta‐analysis of effective online learning approaches, by Holland [54], identified two core factors: interaction opportunities to support learner empowerment and the construction of knowledge; and clearly segmented, titled, or tagged learning objects to enable easy navigation and personalised learning. All of these many factors align with the findings of this review, and place the learner experience at the centre of the design process—digital tools are learning activities foremost, with the technologies being subsidiary considerations.

One of the key strengths of this review was that the analysis and model design were undertaken by two undergraduate students. As a result, the focus on the models and guidance is seen from a learner's perspective. Student feedback on the digital tools in the papers studied was an important factor in determining the efficacy of those tools. The student perspective should be a key feature in any design process for digital (or indeed any) pedagogical approach.

### Implications for biomolecular and biological sciences education

This review highlights the key factors required for a student‐focussed digital resource for general education, although several examples were of direct relevance to the biosciences [22, 24, 34], or medicine [24, 32, 33, 45]. If designing a resource to support scientific content of classes, then interactivity is a fundamental concern, in order to support active learning and reinforcement of learning through retrieval practice [55]. Designing resources to support skills development (academic, communication or laboratory) requires a particular emphasis on interactivity, as evidenced in the virtual flow cytometer of Francis *et al*. [25]. Two fundamental factors underpin successful digital resources for the biosciences. First, the involvement of students in their design and development is essential to ensure that there is a high level of interest and utility for the students. Second, the resource dovetails clearly with the core course content and is seen to support that material rather than force additional new tasks for the learners. Digital tools can have a powerful impact on helping deliver complex bioscience skills and concepts, provided that they are used within a clear context for the learners.

### Limitations and future directions

This review focussed on digital tools developed as learning‐support objects and activities. We acknowledge that digital education comprises a much more comprehensive array of learning affordances, including using online learning platforms and bespoke online teaching, such as MOOCs and distance learning courses. However, the focus on tools designed for specific educational activities enabled an overview of what factors impact stand‐alone learning objects rather than integrated online courses. The review also focussed explicitly on tools used in Higher Education, deliberately excluding those designed for the primary, secondary and adult education sectors. This specificity aimed to identify effective tools for a very specific constituency of learners. There will doubtless be similar factors of importance for other learners, but the learning goals and behaviours of those other learner types will be subtly different. Further work looking at the intersection between these factors in other educational settings would provide a more holistic overview of digital tools in general.

## Conclusions

This systematic review of 25 studies has highlighted the defining features that underpin effective digital tools in higher education, and the challenges that limit their use. Collectively, these findings emphasise that digital tools are most effective when they align closely with course objectives, support rather than complicate learning and provide clear benefits to students. The student‐centred design approach highlighted here aims to support the design of such future digital tools in considering the key elements that lead to effective engagement with those tools by the learners, leading to more efficient learning environments.

With the increased use of online learning to support face‐to‐face pedagogies, the use of digital tools will likely increase in the coming years. The findings highlight the need for careful planning, curriculum integration, iterative testing and responsiveness to student and educator feedback. Digital tools need to be considered as evolving resources that require ongoing refinement, rather than one‐off interventions.

Well‐designed digital tools can foster independence, collaboration and confidence, preparing students for lifelong learning and professional practice. For disciplines with complex skill requirements, such as biosciences and medicine, interactive and adaptive tools offer scalable solutions to reinforce practical competencies. Ultimately, the findings of this review underscore that the value of digital tools lies not in technological novelty but in their capacity to enhance student experience and outcomes through purposeful, student‐centred design.

## Conflict of interest

The authors declare no conflict of interest.

## Author contributions

AA and JH performed the initial literature search and the majority of the article screening. CP, SR and NF contributed to the later stages of article evaluation and decision‐making. AA, JH and CP performed the analysis of the articles, thematic analysis and model design. AA and JH wrote the initial draft of the manuscript. CP, NF and SR refined the manuscript for publication. NF and SR conceived the project.

## Supporting information


**Table S1.** Summary of the papers identified to review.

**Appendix S1.** Bias analysis checklists for the 25 papers included in the study.

**Appendix S2.** PRISMA 2020 checklists.

**Appendix S3.** Claude.ai prompt used to utilise generative artificial intelligence to support and confirm the paper evaluations.

## Data Availability

Data sharing is not applicable to this article as no new data were created or analysed in this study.
