# Supplementary material for: Navigating new norms: a systematic review of factors for the development of effective digital tools in higher education
Source: FEBS Open Bio. 2025 Oct 30;16(3):610–27. doi: 10.1002/2211-5463.70151 (PMC12955751; doi:10.1002/2211-5463.70151)
Supplement: Supplementary file 1 — Table S1. Summary of the papers identified to review. [file FEB4-16-610-s004.docx]

**Supplementary Information S1**

**Table S1 - Summary of the papers identified to review.** The 25 papers identified for review are listed, including the reference number in the main text of this article, the item number in the original list of papers, the year of publication, full citation, and the methodology of the study.

| **Ref** | **Item** | **Year** | **Full Citation** | **Methodology** |
| --- | --- | --- | --- | --- |
| 24 | 30 | 2021 | Nuci, K. P., Tahir, R., Wang, A. I. & Imran, A. S. (2021) Game-Based Digital Quiz as a Tool for Improving Students' Engagement and Learning in Online Lectures, *IEEE Access*, 9, 91220-91234. | Quantitative |
| 25 | 222 | 2022 | Francis, N. J., Ruckley, D. & Wilkinson, T. S. (2022) The virtual flow cytometer: A new learning experience and environment for undergraduate teaching, *Front Educ*, 7. | Mixed methods |
| 26 | 150 | 2022 | James, A. J., Douglas, T. A., Earwaker, L. A. & Mather, C. A. (2022) Student experiences of facilitated asynchronous online discussion boards: Lessons learned and implications for teaching practice, *J Univ Teach Learn Pract*, 19. | Qualitative |
| 27 | 154 | 2020 | Pereira, A. L. M. et al. (2020) Web-Based Virtual Learning Environment for Medicine Administration in Pediatrics and Neonatology: Content Evaluation, *JMIR Serious Games*, 8, 10. | Quantitative |
| 28 | 184 | 2013 | Deperlioglu, O. & Kose, U. (2013) The effectiveness and experiences of blended learning approaches to computer programming education, *Comput Appl Eng Educ*, 21, 328-342. | Mixed methods |
| 29 | 179 | 2018 | Hung, I. C. & Chen, N. S. (2018) Embodied interactive video lectures for improving learning comprehension and retention, *Comput Educ*, 117, 116-131. | Quantitative |
| 30 | 161 | 2021 | Evans, T., Kensington-Miller, B. & Novak, J. (2021) Effectiveness, efficiency, engagement: Mapping the impact of pre-lecture quizzes on educational exchange, *Australas J Educ Technol*, 37, 163-177. | Mixed methods |
| 31 | 198 | 2014 | Yang, Y.-T. C., Gamble, J. H., Hung, Y.-W. & Lin, T.-Y. (2014) An Online Adaptive Learning Environment for Critical-Thinking-Infused English Literacy Instruction, *Br J Educ Technol*, 45, 723-747. | Quantitative |
| 32 | 173 | 2020 | Evenhouse, D., Kandakatla, R., Berger, E., Rhoads, J. F. & DeBoer, J. (2020) Motivators and barriers in undergraduate mechanical engineering students’ use of learning resources, *Eur J Eng Educ*, 45, 879-899. | Qualitative |
| 33 | 169 | 2021 | Lyons, K., Lobczowski, N., Greene, J., Whitley, J. & McLaughlin, J. (2021) Using a design-based research approach to develop and study a web-based tool to support collaborative learning, *Comput Educ*, 161, 104064. | Mixed methods |
| 34 | 181 | 2009 | Shana, Z. (2009) Learning with Technology: Using Discussion Forums to Augment a Traditional-Style Class, *Educ Technol Soc*, 12, 214-228. | Qualitative |
| 35 | 189 | 2007 | Reynolds, P. A., Harper, J., Dunne, S., Cox, M. & Myint, Y. K. (2007) Portable Digital Assistants (PDAs) in dentistry: Part II - Pilot study of PDA use in the dental clinic, *Br Dent J*, 202, 477-483. | Quantitative |
| 36 | 195 | 2016 | Van Es, S. L., Kumar, R. K., Pryor, W. M., Salisbury, E. L. & Velan, G. M. (2016) Cytopathology whole slide images and adaptive tutorials for senior medical students: a randomized crossover trial, *Diagn Pathol*, 11, 9. | Quantitative |
| 37 | 203 | 2019 | Ahmed, M. & Hasegawa, S. (2019) The effects of a new virtual learning platform on improving student skills in designing and producing online virtual laboratories, *Knowl Manage E-Learn*, 11, 364–377. | Quantitative |
| 39 | 15 | 2021 | Estriégana, R., Medina-Merodio, J.-A., Robina-Ramírez, R. & Barchino, R. (2021) Analysis of Cooperative Skills Development through Relational Coordination in a Gamified Online Learning Environment, *Electronics*, 10, 2032. | Quantitative |
| 40 | 168 | 2021 | Lopez-Pernas, S., Gordillo, A., Barra, E. & Quemada, J. (2021) Escapp: A Web Platform for Conducting Educational Escape Rooms, *IEEE Access*, 9, 38062-38077. | Quantitative |
| 41 | 155 | 2022 | Shardlow, M., Sellar, S. & Rousell, D. (2022) Collaborative augmentation and simplification of text (CoAST): pedagogical applications of natural language processing in digital learning environments, *Learn Environ Res*, 25, 399-421. | Mixed methods |
| 45 | 223 | 2019 | Ibtissam, C., Elmostafa, T., Radid, M. & Yazza, Y. (2019) Learning Electrolysis with Podcasting in the Higher Education: From Implementation to Results, *Int J Recent Technol Eng*, 8, 8204-8208. | Quantitative |
| 47 | 167 | 2021 | Mayo-Cubero, M. (2021) Teaching Innovation Experience for COVID-19 Times: A Case Study on Blended Learning of Television Journalism Courses with Moodle, *Asia Pac Media Educ*, 31, 178-194. | Qualitative (case study) |
| 48 | 194 | 2015 | Von Sass, P. F. et al. (2015) Taking a fresh look at the skull base in otorhinolaryngology with web-based simulation: Student's Interactive Skull-Base Trainer (SISTer), *JAMA Otolaryngol Head Neck Surg*, 141, 154-159. | Quantitative |
| 50 | 192 | 2011 | Zhang, H., Song, W. & Burston, J. (2011) Reexamining the effectiveness of vocabulary learning via mobile phones, *Turk Online J Educ Technol*, 10, 203-214. | Quantitative |
| 56 | 157 | 2020 | Alwadei, A. H. et al. (2020) Effectiveness of an adaptive eLearning intervention on dental students’ learning in comparison to traditional instruction, *J Dent Educ*, 84, 1294-1302. | Quantitative |
| 57 | 156 | 2021 | Choo, W. S. (2021) Student perspectives of various learning approaches used in an undergraduate food science and technology subject, *J Food Sci Educ*, 20, 146-154. | Qualitative |
| 58 | 165 | 2020 | Salas-Rueda, R. A. (2020) Impact of the WampServer application in Blended learning considering data science, machine learning, and neural networks, *E-Learn Digit Media*, 17, 199-217. | Quantitative |
| 59 | 170 | 2019 | Pickering, J. D. & Swinnerton, B. J. (2019) Exploring the Dimensions of Medical Student Engagement with Technology-Enhanced Learning Resources and Assessing the Impact on Assessment Outcomes, *Anat Sci Educ*, 12, 117-128. | Mixed methods |
