# Supplementary material for: Navigating new norms: a systematic review of factors for the development of effective digital tools in higher education
Source: FEBS Open Bio. 2025 Oct 30;16(3):610–27. doi: 10.1002/2211-5463.70151 (PMC12955751; doi:10.1002/2211-5463.70151)
Supplement: Supplementary file 2 — Appendix S1. Bias analysis checklists for the 25 papers included in the study. [file FEB4-16-610-s003.docx]

**Appendix S1**

**Bias analysis checklists for the 25 papers included in the study.** Analysis was performed using the JBI evaluation criteria. The ‘Checklist for quasi-experimental studies’ [21] was used for quantitative studies. In those cases where the research was purely qualitative, the ‘Checklist for Qualitative studies’ [22] was used. For mixed-methods studies, both sets of criteria were applied. Papers are listed according to reference number in the main text of this article.

| **RoB Assessor:** SMR | | **Date of Appraisal:** 27/8/25 | | **Record Number:** 30 Ref # 24 | | | | |
| --- | --- | --- | --- | --- | --- | --- | --- | --- |
| **Study Author:** Nuci, K. P., Tahir, R., Wang, A. I. & Imran, A. S. | | **Study Title:** Game-Based Digital Quiz as a Tool for Improving Students' Engagement and Learning in Online Lectures | | **Study Year:** 2021 | | | | |
|  | |  | |  | | | | |
| **Internal Validity** | | | **Choice - Comments/Justification** | | **Yes** | **No** | **Unclear** | **N/A** |
| **Bias related to temporal precedence** | | | | | | | | |
| **1** | **Is it clear in the study what is the “cause” and what is the “effect” (i.e. there is no confusion about which variable comes first)?** | |  | |  |  |  |  |
| **Bias related to selection and allocation** | | | | | | | | |
| **2** | **Was there a control group?** | | Google vs Kahoot | |  |  |  |  |
| **Bias related to confounding factors** | | | | | | | | |
| **3** | **Were participants included in any comparisons similar?** | |  | |  |  |  |  |
| **Bias related to administration of intervention/exposure** | | | | | | | | |
| **4** | **Were the participants included in any comparisons receiving similar treatment/care, other than the exposure or intervention of interest?** | |  | |  |  |  |  |

| **Bias related to assessment, detection and measurement of the outcome** | | | | | | |
| --- | --- | --- | --- | --- | --- | --- |
| **5** | **Were there multiple measurements of the outcome, both pre and post the intervention/exposure?** |  | **Yes** | **No** | **Unclear** | **N/A** |
|  | **Outcome 1** | Impact on knowledge retention |  |  |  |  |
|  | **Outcome 2** | Engagement/Enjoyment |  |  |  |  |
|  | **Outcome 3** | Perceptions of the platform |  |  |  |  |
|  | **Outcome 4** | Learning Curve and Learning Gain |  |  |  |  |
|  | **Outcome 7** |  |  |  |  |  |
|  |  |  |  |  |  |  |
| **6** | **Were the outcomes of participants included in any comparisons measured in the same way?** |  | **Yes** | **No** | **Unclear** | **N/A** |
|  | **Outcome 1** | Impact on knowledge retention |  |  |  |  |
|  | **Outcome 2** | Engagement/Enjoyment |  |  |  |  |
|  | **Outcome 3** | Perceptions of the platform |  |  |  |  |
|  | **Outcome 4** | Learning Curve and Learning Gain |  |  |  |  |
|  | **Outcome 7** |  |  |  |  |  |
|  |  |  |  |  |  |  |
| **7** | **Were outcomes measured in a reliable way?** |  | **Yes** | **No** | **Unclear** | **N/A** |
|  | **Outcome 1** | Impact on knowledge retention |  |  |  |  |
|  | **Outcome 2** | Engagement/Enjoyment |  |  |  |  |
|  | **Outcome 3** | Perceptions of the platform |  |  |  |  |
|  | **Outcome 4** | Learning Curve and Learning Gain |  |  |  |  |
|  | **Outcome 7** |  |  |  |  |  |

| **Bias related to participant retention** | | | | | | | | | | | | | | | |
| --- | --- | --- | --- | --- | --- | --- | --- | --- | --- | --- | --- | --- | --- | --- | --- |
| **8** | **Was follow-up complete and if not, were differences between groups in terms of their follow-up adequately described and analyzed?** | | | | | |  | |  | | | | | | |
|  | **Outcome 1** | | | | | |  | | **Yes** | **No** | | **Unclear** | | **N/A** | |
|  |  | Result 1 | | | | | Fig 3 | |  |  |  | | |  | |
|  | **Outcome 2** | | | | | |  | | **Yes** | **No** | **Unclear** | | | **N/A** | |
|  |  | Result 1 | | | | | Fig 4 | |  |  |  | | |  | |
|  |  | Result 2 | | | | | Table 3 | |  |  |  | | |  | |
|  | **Outcome 3** | | | | | |  | | **Yes** | **No** | **Unclear** | | | **N/A** | |
|  |  | Result 1 | | | | | Fig 5 | |  |  |  | | |  | |
|  | **Outcome 4** | | | | | |  | | **Yes** | **No** | **Unclear** | | | **N/A** | |
|  |  | Result 1 | | | | | Fig 6 | |  |  |  | | |  | |
|  |  | Result 2 | | | | | Fig 7 | |  |  |  | | |  | |
|  |  | Result 3 | | | | | Fig 8 | |  |  |  | | |  | |
|  |  |  | | | | | | |  |  |  | | |  | |
|  | **Statistical Conclusion Validity** | | | | | | | |  |  |  | | |  | |
| **9** | **Was appropriate statistical analysis used?** | | | | | | |  |  | | | | | | |
|  | **Outcome 1** | | |  | | | |  | **Yes** | **No** | **Unclear** | | | **N/A** | |
|  |  | Result 1 | | | | | | Fig 3 |  |  |  | | |  | |
|  | **Outcome 2** | | |  | | | |  | **Yes** | **No** | **Unclear** | | | **N/A** | |
|  |  | Result 1 | | | | | | Fig 4 |  |  |  | | |  | |
|  |  | Result 2 | | | | | | Table 3 |  |  |  | | |  | |
|  | **Outcome 3** | | |  | | | |  | **Yes** | **No** | **Unclear** | | | **N/A** | |
|  |  | Result 1 | | | | | | Fig 5 |  |  |  | | |  | |
|  | **Outcome 4** | | | | | | |  |  | **No** | | | **Unclear** | | **N/A** |
|  |  | Result 1 | | | | | | Fig 6 |  |  | | |  | |  |
|  |  | Result 2 | | | | | | Fig 7 |  |  | | |  | |  |
|  |  | Result 3 | | | | | | Fig 8 |  |  | | |  | |  |
|  | | | | | | | | | | | | | | | |
| **Overall appraisal:** | | | **Include:** | | **Exclude:** | **Seek Further Info:** | | | | | | | | | |
| **Comments:** | | | | | | | | | | | | | | | |

| **RoB Assessor:** SMR | | **Date of Appraisal:** 27/8/25 | | **Record Number:** 222 **Ref #** 25 | | | | |
| --- | --- | --- | --- | --- | --- | --- | --- | --- |
| **Study Author:** Francis, N. J., Ruckley, D. & Wilkinson, T. S. | | **Study Title:**  The virtual flow cytometer: A new learning experience and environment for undergraduate teaching | | **Study Year:** 2022 | | | | |
|  | |  | |  | | | | |
| **Internal Validity** | | | **Choice - Comments/Justification** | | **Yes** | **No** | **Unclear** | **N/A** |
| **Bias related to temporal precedence** | | | | | | | | |
| **1** | **Is it clear in the study what is the “cause” and what is the “effect” (i.e. there is no confusion about which variable comes first)?** | |  | |  |  |  |  |
| **Bias related to selection and allocation** | | | | | | | | |
| **2** | **Was there a control group?** | |  | |  |  |  |  |
| **Bias related to confounding factors** | | | | | | | | |
| **3** | **Were participants included in any comparisons similar?** | |  | |  |  |  |  |
| **Bias related to administration of intervention/exposure** | | | | | | | | |
| **4** | **Were the participants included in any comparisons receiving similar treatment/care, other than the exposure or intervention of interest?** | |  | |  |  |  |  |

| **Bias related to assessment, detection and measurement of the outcome** | | | | | | |
| --- | --- | --- | --- | --- | --- | --- |
| **5** | **Were there multiple measurements of the outcome, both pre and post the intervention/exposure?** |  | **Yes** | **No** | **Unclear** | **N/A** |
|  | **Outcome 1** | Academic performance |  |  |  |  |
|  | **Outcome 2** | Internal consistency |  |  |  |  |
|  | **Outcome 3** | Cohort similarity |  |  |  |  |
|  | **Outcome 4** | Student perceptions |  |  |  |  |
|  | **Outcome 7** |  |  |  |  |  |
|  |  |  |  |  |  |  |
| **6** | **Were the outcomes of participants included in any comparisons measured in the same way?** |  | **Yes** | **No** | **Unclear** | **N/A** |
|  | **Outcome 1** | Academic performance |  |  |  |  |
|  | **Outcome 2** | Internal consistency |  |  |  |  |
|  | **Outcome 3** | Cohort similarity |  |  |  |  |
|  | **Outcome 4** | Student perceptions |  |  |  |  |
|  | **Outcome 7** |  |  |  |  |  |
|  |  |  |  |  |  |  |
| **7** | **Were outcomes measured in a reliable way?** |  | **Yes** | **No** | **Unclear** | **N/A** |
|  | **Outcome 1** | Academic performance |  |  |  |  |
|  | **Outcome 2** | Internal consistency |  |  |  |  |
|  | **Outcome 3** | Cohort similarity |  |  |  |  |
|  | **Outcome 4** | Student perceptions |  |  |  |  |
|  | **Outcome 7** |  |  |  |  |  |

| **Bias related to participant retention** | | | | | | | | | | | | | | | |
| --- | --- | --- | --- | --- | --- | --- | --- | --- | --- | --- | --- | --- | --- | --- | --- |
| **8** | **Was follow-up complete and if not, were differences between groups in terms of their follow-up adequately described and analyzed?** | | | | | |  | |  | | | | | | |
|  | **Outcome 1** | | | | | |  | | **Yes** | **No** | | **Unclear** | | **N/A** | |
|  |  | Result 1 | | | | | Fig 3 | |  |  |  | | |  | |
|  |  | Result 2 | | | | | Fig 4 | |  |  |  | | |  | |
|  |  | Result 3 | | | | |  | |  |  |  | | |  | |
|  | **Outcome 2** | | | | | |  | | **Yes** | **No** | **Unclear** | | | **N/A** | |
|  |  | Result 1 | | | | | Fig 4 | |  |  |  | | |  | |
|  |  | Result 2 | | | | |  | |  |  |  | | |  | |
|  |  | Result 3 | | | | |  | |  |  |  | | |  | |
|  | **Outcome 3** | | | | | |  | | **Yes** | **No** | **Unclear** | | | **N/A** | |
|  |  | Result 1 | | | | | Fig 5 | |  |  |  | | |  | |
|  | **Outcome 4** | | | | | |  | | **Yes** | **No** | **Unclear** | | | **N/A** | |
|  |  | Result 1 | | | | | Table 3 | |  |  |  | | |  | |
|  |  |  | | | | | | |  |  |  | | |  | |
|  | **Statistical Conclusion Validity** | | | | | | | |  |  |  | | |  | |
| **9** | **Was appropriate statistical analysis used?** | | | | | | |  |  | | | | | | |
|  | **Outcome 1** | | |  | | | |  | **Yes** | **No** | **Unclear** | | | **N/A** | |
|  |  | Result 1 | | | | | | Fig 3 |  |  |  | | |  | |
|  |  | Result 2 | | | | | | Fig 4 |  |  |  | | |  | |
|  |  | Result 3 | | | | | |  |  |  |  | | |  | |
|  | **Outcome 2** | | |  | | | |  | **Yes** | **No** | **Unclear** | | | **N/A** | |
|  |  | Result 1 | | | | | | Fig 4 |  |  |  | | |  | |
|  | **Outcome 3** | | |  | | | |  | **Yes** | **No** | **Unclear** | | | **N/A** | |
|  |  | Result 1 | | | | | | Fig 5 |  |  |  | | |  | |
|  | **Outcome 4** | | | | | | |  |  | **No** | | | **Unclear** | | **N/A** |
|  |  | Result 1 | | | | | | Table 3 |  |  | | |  | |  |
|  | | | | | | | | | | | | | | | |
| **Overall appraisal:** | | | **Include:** | | **Exclude:** | **Seek Further Info:** | | | | | | | | | |
| **Comments:** | | | | | | | | | | | | | | | |

© JBI, 2022. All rights reserved. JBI grants use of these

 tools for research purposes only. All other enquiries

 should be sent to[jbisynthesis@adelaide.edu.au](mailto:jbisynthesis@adelaide.edu.au)

JBI Critical Appraisal Checklist for
Qualitative Research

Reviewer: S. Rutherford Date: 26/08/2025

Author: . Francis, N. J., Ruckley, D. & Wilkinson, T. S. (2022) The virtual flow cytometer: A new learning experience and environment for undergraduate teaching, *Front Educ*. **7.**

Year: 2022 Record Number: 169 Ref#25

|  | Yes | No | Unclear | Not applicable |
| --- | --- | --- | --- | --- |
| 1. Is there congruity between the stated philosophical perspective and the research methodology? | x | □ | □ | □ |
| 1. Is there congruity between the research methodology and the research question or objectives? | x | □ | □ | □ |
| 1. Is there congruity between the research methodology and the methods used to collect data? | x | □ | □ | □ |
| 1. Is there congruity between the research methodology and the representation and analysis of data? | x | □ | □ | □ |
| 1. Is there congruity between the research methodology and the interpretation of results? | x | □ | □ | □ |
| 1. Is there a statement locating the researcher culturally or theoretically? | □ | x | □ | □ |
| 1. Is the influence of the researcher on the research, and vice- versa, addressed? | x | □ | □ | □ |
| 1. Are participants, and their voices, adequately represented? | x | □ | □ | □ |
| 1. Is the research ethical according to current criteria or, for recent studies, and is there evidence of ethical approval by an appropriate body? | x | □ | □ | □ |
| 1. Do the conclusions drawn in the research report flow from the analysis, or interpretation, of the data? | x | □ | □ | □ |

Overall appraisal: Include x Exclude □ Seek further info □

Comments (Including reason for exclusion): Mixed Methods, thematic analysis of the survey responses, but most of the paper is quantitative..

JBI Critical Appraisal Checklist for
Qualitative Research

Reviewer: S. Rutherford Date: 26/08/2025

Author: James, A. J., Douglas, T. A., Earwaker, L. A. & Mather, C. A. (2022) Student experiences of facilitated asynchronous online discussion boards: Lessons learned and implications for teaching practice, *J Univ Teach Learn Pract*, 19.

Year: 2022 Record Number: 150 Ref#26

|  | Yes | No | Unclear | Not applicable |
| --- | --- | --- | --- | --- |
| 1. Is there congruity between the stated philosophical perspective and the research methodology? | □ | □ | X | □ |
| 1. Is there congruity between the research methodology and the research question or objectives? | X | □ | □ | □ |
| 1. Is there congruity between the research methodology and the methods used to collect data? | X | □ | □ | □ |
| 1. Is there congruity between the research methodology and the representation and analysis of data? | X | □ | □ | □ |
| 1. Is there congruity between the research methodology and the interpretation of results? | X | □ | □ | □ |
| 1. Is there a statement locating the researcher culturally or theoretically? | □ | X | □ | □ |
| 1. Is the influence of the researcher on the research, and vice- versa, addressed? | □ | X | □ | □ |
| 1. Are participants, and their voices, adequately represented? | X | □ | □ | □ |
| 1. Is the research ethical according to current criteria or, for recent studies, and is there evidence of ethical approval by an appropriate body? | X | □ | □ | □ |
| 1. Do the conclusions drawn in the research report flow from the analysis, or interpretation, of the data? | X | □ | □ | □ |

Overall appraisal: Include X Exclude □ Seek further info □

Comments (Including reason for exclusion): The article describes qualitative methods and thematic analysis but does not explicitly state a philosophical or theoretical perspective.

| **RoB Assessor:** SMR | | **Date of Appraisal:** 27/8/25 | | **Record Number:** 154 Ref # 27 | | | | |
| --- | --- | --- | --- | --- | --- | --- | --- | --- |
| **Study Author:** Pereira, A. L. M., Leon, C., Ribeiro, L. M., Brasil, G. C., Carneiro, K. K. G., Vieira, G. B., Barbalho, Y. G. D., Da Silva, I. C. R. & Funghetto, S. S. | | **Study Title:**  Web-Based Virtual Learning Environment for Medicine Administration in Pediatrics and Neonatology: Content Evaluation | | **Study Year:** 2020 | | | | |
|  | |  | |  | | | | |
| **Internal Validity** | | | **Choice - Comments/Justification** | | **Yes** | **No** | **Unclear** | **N/A** |
| **Bias related to temporal precedence** | | | | | | | | |
| **1** | **Is it clear in the study what is the “cause” and what is the “effect” (i.e. there is no confusion about which variable comes first)?** | | The implementation of a virtual learning environment (VLE) focused on pediatric and neonatal medication administration. Content validation by experts, user feedback from students, and knowledge gains measured via pretest–posttest comparisons. | |  |  |  |  |
| **Bias related to selection and allocation** | | | | | | | | |
| **2** | **Was there a control group?** | | The study employs expert validation and student evaluation, along with pretest–posttest measures, consistent with design-based descriptive methodology | |  |  |  |  |
| **Bias related to confounding factors** | | | | | | | | |
| **3** | **Were participants included in any comparisons similar?** | | Experts vs students | |  |  |  |  |
| **Bias related to administration of intervention/exposure** | | | | | | | | |
| **4** | **Were the participants included in any comparisons receiving similar treatment/care, other than the exposure or intervention of interest?** | | All participants in both expert and student groups interacted with the same VLE under similar conditions. | |  |  |  |  |

| **Bias related to assessment, detection and measurement of the outcome** | | | | | | |
| --- | --- | --- | --- | --- | --- | --- |
| **5** | **Were there multiple measurements of the outcome, both pre and post the intervention/exposure?** |  | **Yes** | **No** | **Unclear** | **N/A** |
|  | **Outcome 1** | Knowledge gain |  |  |  |  |
|  |  |  |  |  |  |  |
| **6** | **Were the outcomes of participants included in any comparisons measured in the same way?** |  | **Yes** | **No** | **Unclear** | **N/A** |
|  | **Outcome 1** | Knowledge gain |  |  |  |  |
|  |  |  |  |  |  |  |
| **7** | **Were outcomes measured in a reliable way?** |  | **Yes** | **No** | **Unclear** | **N/A** |
|  | **Outcome 1** | Knowledge gain |  |  |  |  |

| **Bias related to participant retention** | | | | | | | | | | | | | |
| --- | --- | --- | --- | --- | --- | --- | --- | --- | --- | --- | --- | --- | --- |
| **8** | **Was follow-up complete and if not, were differences between groups in terms of their follow-up adequately described and analyzed?** | | | | | |  | |  | | | | |
|  | **Outcome 1** | | | | | | Knowledge gain | | **Yes** | **No** | | **Unclear** | **N/A** |
|  |  | Result 1 | | | | | Figure 3 | |  |  |  | |  |
|  |  | Result 2 | | | | | Table 4 | |  |  |  | |  |
|  |  |  | | | | | | |  |  |  | |  |
|  | **Statistical Conclusion Validity** | | | | | | | |  |  |  | |  |
| **9** | **Was appropriate statistical analysis used?** | | | | | | |  |  | | | | |
|  | **Outcome 1** | | |  | | | | Knowledge gain | **Yes** | **No** | **Unclear** | | **N/A** |
|  |  | Result 1 | | | | | | Figure 3 |  |  |  | |  |
|  |  | Result 2 | | | | | | Table 4 |  |  |  | |  |
|  | | | | | | | | | | | | | |
| **Overall appraisal:** | | | **Include:** | | **Exclude:** | **Seek Further Info:** | | | | | | | |
| **Comments:** | | | | | | | | | | | | | |

© JBI, 2022. All rights reserved. JBI grants use of these

 tools for research purposes only. All other enquiries

 should be sent to[jbisynthesis@adelaide.edu.au](mailto:jbisynthesis@adelaide.edu.au)

| **RoB Assessor:** SMR | | **Date of Appraisal:** 27/8/25 | | **Record Number:** 184 Ref # 28 | | | | |
| --- | --- | --- | --- | --- | --- | --- | --- | --- |
| **Study Author:** Deperlioglu & Kose | | **Study Title:** The effectiveness and experiences of blended learning approaches to computer programming education | | **Study Year:** 2013 | | | | |
|  | |  | |  | | | | |
| **Internal Validity** | | | **Choice - Comments/Justification** | | **Yes** | **No** | **Unclear** | **N/A** |
| **Bias related to temporal precedence** | | | | | | | | |
| **1** | **Is it clear in the study what is the “cause” and what is the “effect” (i.e. there is no confusion about which variable comes first)?** | |  | |  |  |  |  |
| **Bias related to selection and allocation** | | | | | | | | |
| **2** | **Was there a control group?** | |  | |  |  |  |  |
| **Bias related to confounding factors** | | | | | | | | |
| **3** | **Were participants included in any comparisons similar?** | |  | |  |  |  |  |
| **Bias related to administration of intervention/exposure** | | | | | | | | |
| **4** | **Were the participants included in any comparisons receiving similar treatment/care, other than the exposure or intervention of interest?** | |  | |  |  |  |  |

| **Bias related to assessment, detection and measurement of the outcome** | | | | | | |
| --- | --- | --- | --- | --- | --- | --- |
| **5** | **Were there multiple measurements of the outcome, both pre and post the intervention/exposure?** |  | **Yes** | **No** | **Unclear** | **N/A** |
|  | **Outcome 1** | Results |  |  |  |  |
|  | **Outcome 2** | Grade Ranges |  |  |  |  |
|  | **Outcome 3** | Survey |  |  |  |  |
|  | **Outcome 4** |  |  |  |  |  |
|  |  |  |  |  |  |  |
| **6** | **Were the outcomes of participants included in any comparisons measured in the same way?** |  | **Yes** | **No** | **Unclear** | **N/A** |
|  | **Outcome 1** | Results |  |  |  |  |
|  | **Outcome 2** | Grade Ranges |  |  |  |  |
|  | **Outcome 3** | Survey |  |  |  |  |
|  | **Outcome 4** |  |  |  |  |  |
|  |  |  |  |  |  |  |
| **7** | **Were outcomes measured in a reliable way?** |  | **Yes** | **No** | **Unclear** | **N/A** |
|  | **Outcome 1** | Results |  |  |  |  |
|  | **Outcome 2** | Grade Ranges |  |  |  |  |
|  | **Outcome 3** | Survey |  |  |  |  |
|  | **Outcome 4** |  |  |  |  |  |

| **Bias related to participant retention** | | | | | | | | | | | | | |
| --- | --- | --- | --- | --- | --- | --- | --- | --- | --- | --- | --- | --- | --- |
| **8** | **Was follow-up complete and if not, were differences between groups in terms of their follow-up adequately described and analyzed?** | | | | | |  | |  | | | | |
|  | **Outcome 1** | | | | | |  | | **Yes** | **No** | | **Unclear** | **N/A** |
|  |  | Result 1 | | | | | Table 3 | |  |  |  | |  |
|  | **Outcome 2** | | | | | |  | | **Yes** | **No** | **Unclear** | | **N/A** |
|  |  | Result 1 | | | | | Figure 13 | |  |  |  | |  |
|  |  | Result 2 | | | | |  | |  |  |  | |  |
|  |  | Result 3 | | | | |  | |  |  |  | |  |
|  |  |  | | | | | | |  |  |  | |  |
|  | **Statistical Conclusion Validity** | | | | | | | |  |  |  | |  |
| **9** | **Was appropriate statistical analysis used?** | | | | | | |  |  | | | | |
|  | **Outcome 1** | | |  | | | |  | **Yes** | **No** | **Unclear** | | **N/A** |
|  |  | Result 1 | | | | | | Table 3 |  |  |  | |  |
|  |  | Result 2 | | | | | |  |  |  |  | |  |
|  |  | Result 3 | | | | | |  |  |  |  | |  |
|  | **Outcome 2** | | |  | | | |  | **Yes** | **No** | **Unclear** | | **N/A** |
|  |  | Result 1 | | | | | | Figure 13 |  |  |  | |  |
|  | **Outcome 3** | | |  | | | |  | **Yes** | **No** | **Unclear** | | **N/A** |
|  |  | Result 1 | | | | | | Table 4 |  |  |  | |  |
|  | | | | | | | | | | | | | |
| **Overall appraisal:** | | | **Include:** | | **Exclude:** | **Seek Further Info:** | | | | | | | |
| **Comments:** | | | | | | | | | | | | | |

© JBI, 2022. All rights reserved. JBI grants use of these

 tools for research purposes only. All other enquiries

 should be sent to[jbisynthesis@adelaide.edu.au](mailto:jbisynthesis@adelaide.edu.au)

JBI Critical Appraisal Checklist for
Qualitative Research

Reviewer: S. Rutherford Date: 26/08/2025

Author: Deperlioglu, O. & Kose, U. (2013) The effectiveness and experiences of blended learning approaches to computer programming education, *Comput Appl Eng Educ*, 21, 328-342

Year: 2013 Record Number: 184 Ref# 28

|  | Yes | No | Unclear | Not applicable |
| --- | --- | --- | --- | --- |
| 1. Is there congruity between the stated philosophical perspective and the research methodology? | □ | x | □ | □ |
| 1. Is there congruity between the research methodology and the research question or objectives? | x | □ | □ | □ |
| 1. Is there congruity between the research methodology and the methods used to collect data? | x | □ | □ | □ |
| 1. Is there congruity between the research methodology and the representation and analysis of data? | □ | x | □ | □ |
| 1. Is there congruity between the research methodology and the interpretation of results? | x | □ | □ | □ |
| 1. Is there a statement locating the researcher culturally or theoretically? | □ | x | □ | □ |
| 1. Is the influence of the researcher on the research, and vice- versa, addressed? | □ | x | □ | □ |
| 1. Are participants, and their voices, adequately represented? | □ | □ | x | □ |
| 1. Is the research ethical according to current criteria or, for recent studies, and is there evidence of ethical approval by an appropriate body? | □ | □ | x | □ |
| 1. Do the conclusions drawn in the research report flow from the analysis, or interpretation, of the data? | x | □ | □ | □ |

Overall appraisal: Include X Exclude □ Seek further info □

Comments (Including reason for exclusion):

| **RoB Assessor:** SMR | | **Date of Appraisal:** 27/8/25 | | **Record Number:** 179 Ref # 29 | | | | |
| --- | --- | --- | --- | --- | --- | --- | --- | --- |
| **Study Author:** Hung and Chen | | **Study Title:** Embodied interactive video lectures for improving learning comprehension and retention | | **Study Year:** 2018 | | | | |
|  | |  | |  | | | | |
| **Internal Validity** | | | **Choice - Comments/Justification** | | **Yes** | **No** | **Unclear** | **N/A** |
| **Bias related to temporal precedence** | | | | | | | | |
| **1** | **Is it clear in the study what is the “cause” and what is the “effect” (i.e. there is no confusion about which variable comes first)?** | | Learners' comprehension and retention of the learning content, as well as their cognitive load during learning. | |  |  |  |  |
| **Bias related to selection and allocation** | | | | | | | | |
| **2** | **Was there a control group?** | | The conventional video lecture group serves as a control, representing passive watching without interactivity.  The non-embodied interactive group (interactive but without embodied gestures) offers a form of active control, allowing comparison against the full embodied experience. | |  |  |  |  |
| **Bias related to confounding factors** | | | | | | | | |
| **3** | **Were participants included in any comparisons similar?** | | The participants were 90 university students, randomly and equally assigned to the three groups | |  |  |  |  |
| **Bias related to administration of intervention/exposure** | | | | | | | | |
| **4** | **Were the participants included in any comparisons receiving similar treatment/care, other than the exposure or intervention of interest?** | | All participants were exposed to video lectures with the same content, duration, and learning objectives.  The only systematic difference was the format: presence/absence of embodied gestures and interactivity. Hence, aside from the intervention (media format), all others were kept consistent | |  |  |  |  |

| **Bias related to assessment, detection and measurement of the outcome** | | | | | | |
| --- | --- | --- | --- | --- | --- | --- |
| **5** | **Were there multiple measurements of the outcome, both pre and post the intervention/exposure?** |  | **Yes** | **No** | **Unclear** | **N/A** |
|  | **Outcome 1** | Knowledge Test |  |  |  |  |
|  | **Outcome 2** | Delayed Test |  |  |  |  |
|  | **Outcome 3** | Perceived Difficulty |  |  |  |  |
|  | **Outcome 4** |  |  |  |  |  |
|  |  |  |  |  |  |  |
| **6** | **Were the outcomes of participants included in any comparisons measured in the same way?** |  | **Yes** | **No** | **Unclear** | **N/A** |
|  | **Outcome 1** | Knowledge Test |  |  |  |  |
|  | **Outcome 2** | Delayed Test |  |  |  |  |
|  | **Outcome 3** | Perceived Difficulty |  |  |  |  |
|  | **Outcome 4** |  |  |  |  |  |
|  |  |  |  |  |  |  |
| **7** | **Were outcomes measured in a reliable way?** |  | **Yes** | **No** | **Unclear** | **N/A** |
|  | **Outcome 1** | Knowledge Test |  |  |  |  |
|  | **Outcome 2** | Delayed Test |  |  |  |  |
|  | **Outcome 3** | Perceived Difficulty |  |  |  |  |

| **Bias related to participant retention** | | | | | | | | | | | | | | | |
| --- | --- | --- | --- | --- | --- | --- | --- | --- | --- | --- | --- | --- | --- | --- | --- |
| **8** | **Was follow-up complete and if not, were differences between groups in terms of their follow-up adequately described and analyzed?** | | | | | |  | |  | | | | | | |
|  | **Outcome 1** | | | | | | Knowledge test | | **Yes** | **No** | | **Unclear** | | **N/A** | |
|  |  | Result 1 | | | | | Fig 10 | |  |  |  | | |  | |
|  |  | Result 2 | | | | | Table 4 | |  |  |  | | |  | |
|  |  | Result 3 | | | | | Table 5 | |  |  |  | | |  | |
|  | **Outcome 2** | | | | | | Delayed Test | | **Yes** | **No** | **Unclear** | | | **N/A** | |
|  |  | Result 1 | | | | | Figure 11 | |  |  |  | | |  | |
|  |  | Result 2 | | | | | Table 7 | |  |  |  | | |  | |
|  |  | Result 3 | | | | |  | |  |  |  | | |  | |
|  | **Outcome 3** | | | | | | Perceived Difficulty | | **Yes** | **No** | **Unclear** | | | **N/A** | |
|  |  | Result 1 | | | | | Table 8 | |  |  |  | | |  | |
|  |  |  | | | | | | |  |  |  | | |  | |
|  | **Statistical Conclusion Validity** | | | | | | | |  |  |  | | |  | |
| **9** | **Was appropriate statistical analysis used?** | | | | | | |  |  | | | | | | |
|  | **Outcome 1** | | |  | | | | Knowledge test | **Yes** | **No** | **Unclear** | | | **N/A** | |
|  |  | Result 1 | | | | | | Fig 10 |  |  |  | | |  | |
|  |  | Result 2 | | | | | | Table 4 |  |  |  | | |  | |
|  |  | Result 3 | | | | | | Table 5 |  |  |  | | |  | |
|  | **Outcome 2** | | |  | | | | Delayed Test | **Yes** | **No** | **Unclear** | | | **N/A** | |
|  |  | Result 1 | | | | | | Figure 11 |  |  |  | | |  | |
|  |  | Result 2 | | | | | | Table 7 |  |  |  | | |  | |
|  |  | Result 3 | | | | | |  |  |  |  | | |  | |
|  | **Outcome 3** | | | | | | | Perceived Difficulty | **Yes** | **No** | | | **Unclear** | | **N/A** |
|  |  | Result 1 | | | | | | Table 8 |  |  | | |  | |  |
|  |  | Result 2 | | | | | |  |  |  | | |  | |  |
|  | | | | | | | | | | | | | | | |
| **Overall appraisal:** | | | **Include:** | | **Exclude:** | **Seek Further Info:** | | | | | | | | | |
| **Comments:** The authors clearly establish the cause (media format) and effect (learning performance and cognitive load), include proper control conditions, and ensure comparability across groups. | | | | | | | | | | | | | | | |

© JBI, 2022. All rights reserved. JBI grants use of these

 tools for research purposes only. All other enquiries

 should be sent to[jbisynthesis@adelaide.edu.au](mailto:jbisynthesis@adelaide.edu.au)

| **RoB Assessor:** SMR | | **Date of Appraisal:** 27/8/25 | | | | **Record Number:** 161 Ref # 30 | | | | | |
| --- | --- | --- | --- | --- | --- | --- | --- | --- | --- | --- | --- |
| **Study Author:** Evans, T., Kensington-Miller, B. & Novak, J. | | **Study Title:** Effectiveness, efficiency, engagement: Mapping the impact of pre-lecture quizzes on educational exchange | | | | **Study Year:** 2021 | | | | | |
|  | |  | |  | | | | | |  |  |
| **Internal Validity** | | | **Choice - Comments/Justification** | | **Yes** | | **No** | **Unclear** | **N/A** | |  |
| **Bias related to temporal precedence** | | | | | | | | | | |  |
| **1** | **Is it clear in the study what is the “cause” and what is the “effect” (i.e. there is no confusion about which variable comes first)?** | | Cause: Introduction of regular online pre-lecture quizzes in a university mathematics course, intended to encourage spaced practice.  Effect: Changes in student engagement (frequency and quality), lecture attendance, and academic performance (grades) | |  | |  |  |  | |  |
| **Bias related to selection and allocation** | | | | | | | | | | |  |
| **2** | **Was there a control group?** | | The study compares one semester with pre-lecture quizzes to previous semesters without them.  There’s no randomized control group; rather, a historical comparison serves as the control | |  | |  |  |  | |  |
| **Bias related to confounding factors** | | | | | | | | | | |  |
| **3** | **Were participants included in any comparisons similar?** | | All participants were students in the same undergraduate mathematics course at the University of Auckland—differences between cohorts are minimized by consistent course context.  While demographic or baseline academic characteristics aren’t detailed in the summary, the consistent course framework supports comparability | |  | |  |  |  | |  |
| **Bias related to administration of intervention/exposure** | | | | | | | | | | |  |
| **4** | **Were the participants included in any comparisons receiving similar treatment/care, other than the exposure or intervention of interest?** | | Aside from the quiz intervention, instruction, course content, and structure appear to be consistent across semesters.  The summary implies that only the addition of quizzes varied; other teaching elements remained stable | |  | |  |  |  | |  |

| **Bias related to assessment, detection and measurement of the outcome** | | | | | | |
| --- | --- | --- | --- | --- | --- | --- |
| **5** | **Were there multiple measurements of the outcome, both pre and post the intervention/exposure?** |  | **Yes** | **No** | **Unclear** | **N/A** |
|  | **Outcome 1** | Time spent studying |  |  |  |  |
|  | **Outcome 2** | Student survey |  |  |  |  |
|  | **Outcome 3** | Grades Comparison |  |  |  |  |
|  | **Outcome 4** |  |  |  |  |  |
|  |  |  |  |  |  |  |
| **6** | **Were the outcomes of participants included in any comparisons measured in the same way?** |  | **Yes** | **No** | **Unclear** | **N/A** |
|  | **Outcome 1** | Time spent studying |  |  |  |  |
|  | **Outcome 2** | Student survey |  |  |  |  |
|  | **Outcome 3** | Grades Comparison |  |  |  |  |
|  | **Outcome 7** |  |  |  |  |  |
|  |  |  |  |  |  |  |
| **7** | **Were outcomes measured in a reliable way?** |  | **Yes** | **No** | **Unclear** | **N/A** |
|  | **Outcome 1** | Time spent studying |  |  |  |  |
|  | **Outcome 2** | Student survey |  |  |  |  |
|  | **Outcome 3** | Grades Comparison |  |  |  |  |
|  | **Outcome 7** |  |  |  |  |  |

| **Bias related to participant retention** | | | | | | | | | | | | | | | |
| --- | --- | --- | --- | --- | --- | --- | --- | --- | --- | --- | --- | --- | --- | --- | --- |
| **8** | **Was follow-up complete and if not, were differences between groups in terms of their follow-up adequately described and analyzed?** | | | | | |  | |  | | | | | | |
|  | **Outcome 1** | | | | | | Time spent studying | | **Yes** | **No** | | **Unclear** | | **N/A** | |
|  |  | Result 1 | | | | | Figure 3 | |  |  |  | | |  | |
|  |  | Result 2 | | | | | Figure 4 | |  |  |  | | |  | |
|  |  | Result 3 | | | | |  | |  |  |  | | |  | |
|  | **Outcome 2** | | | | | | Grades Comparison | | **Yes** | **No** | **Unclear** | | | **N/A** | |
|  |  | Result 1 | | | | | Figure 5 | |  |  |  | | |  | |
|  |  | Result 2 | | | | |  | |  |  |  | | |  | |
|  |  | Result 3 | | | | |  | |  |  |  | | |  | |
|  |  |  | | | | | | |  |  |  | | |  | |
|  | **Statistical Conclusion Validity** | | | | | | | |  |  |  | | |  | |
| **9** | **Was appropriate statistical analysis used?** | | | | | | |  |  | | | | | | |
|  | **Outcome 1** | | |  | | | | Time spent studying | **Yes** | **No** | **Unclear** | | | **N/A** | |
|  |  | Result 1 | | | | | | Figure 3 |  |  |  | | |  | |
|  |  | Result 2 | | | | | | Figure 4 |  |  |  | | |  | |
|  |  | Result 3 | | | | | |  |  |  |  | | |  | |
|  | **Outcome 2** | | |  | | | | Grades Comparison | **Yes** | **No** | **Unclear** | | | **N/A** | |
|  |  | Result 1 | | | | | | Figure 5 |  |  |  | | |  | |
|  |  | Result 3 | | | | | |  |  |  | | |  | |  |
|  | | | | | | | | | | | | | | | |
| **Overall appraisal:** | | | **Include:** | | **Exclude:** | **Seek Further Info:** | | | | | | | | | |
| **Comments:** | | | | | | | | | | | | | | | |

© JBI, 2022. All rights reserved. JBI grants use of these

 tools for research purposes only. All other enquiries

 should be sent to[jbisynthesis@adelaide.edu.au](mailto:jbisynthesis@adelaide.edu.au)

JBI Critical Appraisal Checklist for
Qualitative Research

Reviewer: S. Rutherford Date: 27/08/2025

Author: Evans, T., Kensington-Miller, B. & Novak, J. (2021) Effectiveness, efficiency, engagement: Mapping the impact of pre-lecture quizzes on educational exchange, *Australasian Journal of Educational Technology*. **37**, 163-177

Year: 2021 Record Number: 161 Ref #30

|  | Yes | No | Unclear | Not applicable |
| --- | --- | --- | --- | --- |
| 1. Is there congruity between the stated philosophical perspective and the research methodology? | x | □ | □ | □ |
| 1. Is there congruity between the research methodology and the research question or objectives? | x | □ | □ | □ |
| 1. Is there congruity between the research methodology and the methods used to collect data? | x | □ | □ | □ |
| 1. Is there congruity between the research methodology and the representation and analysis of data? | x | □ | □ | □ |
| 1. Is there congruity between the research methodology and the interpretation of results? | x | □ | □ | □ |
| 1. Is there a statement locating the researcher culturally or theoretically? | □ | x | □ | □ |
| 1. Is the influence of the researcher on the research, and vice- versa, addressed? | x | □ | □ | □ |
| 1. Are participants, and their voices, adequately represented? | □ | x | □ | □ |
| 1. Is the research ethical according to current criteria or, for recent studies, and is there evidence of ethical approval by an appropriate body? | □ | x | □ | □ |
| 1. Do the conclusions drawn in the research report flow from the analysis, or interpretation, of the data? | x | □ | □ | □ |

Overall appraisal: Include x Exclude □ Seek further info □

Comments (Including reason for exclusion): Mixed Methods, so some qualitative approaches less relevant. Conclusions do align with the data, but qualitative results are more for support than proving hypotheses.

| **RoB Assessor:** SMR | | **Date of Appraisal:** 4/9/25 | | **Record Number:** 198 Ref # 31 | | | | |
| --- | --- | --- | --- | --- | --- | --- | --- | --- |
| **Study Author:** Yang, Y.-T. C., Gamble, J. H., Hung, Y.-W. & Lin, T.-Y. | | **Study Title:** An Online Adaptive Learning Environment for Critical-Thinking-Infused English Literacy Instruction | | **Study Year:** 2014 | | | | |
|  | |  | |  | | | | |
| **Internal Validity** | | | **Choice - Comments/Justification** | | **Yes** | **No** | **Unclear** | **N/A** |
| **Bias related to temporal precedence** | | | | | | | | |
| **1** | **Is it clear in the study what is the “cause” and what is the “effect” (i.e. there is no confusion about which variable comes first)?** | | Cause (intervention): A critical-thinking infused adaptive English literacy instruction delivered via a Moodle-based blended adaptive learning environment. This included grouping students by proficiency, providing level-specific content, and individualized feedback.  Effect (outcomes): Improvements in students’ critical thinking skills (CTS) and English literacy, and elevated quality of student online discussions.  The study clearly structures the intervention preceding and potentially causing the outcomes | |  |  |  |  |
| **Bias related to selection and allocation** | | | | | | | | |
| **2** | **Was there a control group?** | | The study employed a one-group pre test/post test design with no separate control or comparison group. The authors note institutional constraints prevented inclusion of a non-adaptive comparator | |  |  |  |  |
| **Bias related to confounding factors** | | | | | | | | |
| **3** | **Were participants included in any comparisons similar?** | | Participants were 83 freshman undergraduates enrolled in the same general studies course, sharing similar educational backgrounds | |  |  |  |  |
| **Bias related to administration of intervention/exposure** | | | | | | | | |
| **4** | **Were the participants included in any comparisons receiving similar treatment/care, other than the exposure or intervention of interest?** | | All participants received the same blended instruction, which combined CT workshops, face-to-face sessions, supervised Moodle-based adaptive modules, and access to materials. The only variation lay in their grouping for proficiency-based instruction, which is part of the adaptive intervention, not an extraneous variable | |  |  |  |  |

| **Bias related to assessment, detection and measurement of the outcome** | | | | | | |
| --- | --- | --- | --- | --- | --- | --- |
| **5** | **Were there multiple measurements of the outcome, both pre and post the intervention/exposure?** |  | **Yes** | **No** | **Unclear** | **N/A** |
|  | **Outcome 1** | HCTSR and CCTST scores |  |  |  |  |
|  | **Outcome 2** | Questioning |  |  |  |  |
|  | **Outcome 3** | Percentage of online discussion messages |  |  |  |  |
|  | **Outcome 7** |  |  |  |  |  |
|  |  |  |  |  |  |  |
| **6** | **Were the outcomes of participants included in any comparisons measured in the same way?** |  | **Yes** | **No** | **Unclear** | **N/A** |
|  | **Outcome 1** | HCTSR and CCTST scores |  |  |  |  |
|  | **Outcome 2** | Questioning |  |  |  |  |
|  | **Outcome 3** | Percentage of online discussion messages |  |  |  |  |
|  | **Outcome 7** |  |  |  |  |  |
|  |  |  |  |  |  |  |
| **7** | **Were outcomes measured in a reliable way?** |  | **Yes** | **No** | **Unclear** | **N/A** |
|  | **Outcome 1** | HCTSR and CCTST scores |  |  |  |  |
|  | **Outcome 2** | Questioning |  |  |  |  |
|  | **Outcome 3** | Percentage of online discussion messages |  |  |  |  |
|  | **Outcome 7** |  |  |  |  |  |

| **Bias related to participant retention** | | | | | | | | | | | | | | | |
| --- | --- | --- | --- | --- | --- | --- | --- | --- | --- | --- | --- | --- | --- | --- | --- |
| **8** | **Was follow-up complete and if not, were differences between groups in terms of their follow-up adequately described and analyzed?** | | | | | |  | |  | | | | | | |
|  | **Outcome 1** | | | | | | HCTSR and CCTST scores | | **Yes** | **No** | | **Unclear** | | **N/A** | |
|  |  | Result 1 | | | | | Tables 3-6 | |  |  |  | | |  | |
|  |  | Result 2 | | | | | Table 7 | |  |  |  | | |  | |
|  |  | Result 3 | | | | |  | |  |  |  | | |  | |
|  | **Outcome 2** | | | | | | Questioning | | **Yes** | **No** | **Unclear** | | | **N/A** | |
|  |  | Result 1 | | | | | Table 8 | |  |  |  | | |  | |
|  |  | Result 2 | | | | |  | |  |  |  | | |  | |
|  |  | Result 3 | | | | |  | |  |  |  | | |  | |
|  | **Outcome 3** | | | | | | Percentage of online discussion messages | | **Yes** | **No** | **Unclear** | | | **N/A** | |
|  |  | Result 1 | | | | | Fig 10 | |  |  |  | | |  | |
|  |  | Result 3 | | | | |  | |  |  |  | | |  | |
|  |  |  | | | | | | |  |  |  | | |  | |
|  | **Statistical Conclusion Validity** | | | | | | | |  |  |  | | |  | |
| **9** | **Was appropriate statistical analysis used?** | | | | | | |  |  | | | | | | |
|  | **Outcome 1** | | |  | | | | HCTSR and CCTST scores | **Yes** | **No** | **Unclear** | | | **N/A** | |
|  |  | Result 1 | | | | | | Tables 3-6 |  |  |  | | |  | |
|  |  | Result 2 | | | | | | Table 7 |  |  |  | | |  | |
|  |  | Result 3 | | | | | |  |  |  |  | | |  | |
|  | **Outcome 2** | | |  | | | | Questioning | **Yes** | **No** | **Unclear** | | | **N/A** | |
|  |  | Result 1 | | | | | | Table 8 |  |  |  | | |  | |
|  |  | Result 2 | | | | | |  |  |  |  | | |  | |
|  | **Outcome 3** | | |  | | | | Percentage of online discussion messages | **Yes** | **No** | **Unclear** | | | **N/A** | |
|  |  | Result 1 | | | | | | Fig 10 |  |  |  | | |  | |
|  |  | Result 2 | | | | | |  |  |  | | |  | |  |
|  | | | | | | | | | | | | | | | |
| **Overall appraisal:** | | | **Include:** | | **Exclude:** | **Seek Further Info:** | | | | | | | | | |
| **Comments:** | | | | | | | | | | | | | | | |

© JBI, 2022. All rights reserved. JBI grants use of these

 tools for research purposes only. All other enquiries

 should be sent to[jbisynthesis@adelaide.edu.au](mailto:jbisynthesis@adelaide.edu.au)

JBI Critical Appraisal Checklist for
Qualitative Research

Reviewer: S. Rutherford Date: 26/08/2025

Author: Evenhouse, D., Kandakatla, R., Berger, E., Rhoads, J. F. & DeBoer, J. (2020) Motivators and barriers in undergraduate mechanical engineering students’ use of learning resources, *Eur J Eng Educ*, 45, 879-899.

Year: 2020 Record Number: 173 Ref #32

|  | Yes | No | Unclear | Not applicable |
| --- | --- | --- | --- | --- |
| 1. Is there congruity between the stated philosophical perspective and the research methodology? | X | □ | □ | □ |
| 1. Is there congruity between the research methodology and the research question or objectives? | X | □ | □ | □ |
| 1. Is there congruity between the research methodology and the methods used to collect data? | X | □ | □ | □ |
| 1. Is there congruity between the research methodology and the representation and analysis of data? | X | □ | □ | □ |
| 1. Is there congruity between the research methodology and the interpretation of results? | X | □ | □ | □ |
| 1. Is there a statement locating the researcher culturally or theoretically? | □ | □ | X | □ |
| 1. Is the influence of the researcher on the research, and vice- versa, addressed? | □ | □ | X | □ |
| 1. Are participants, and their voices, adequately represented? | X | □ | □ | □ |
| 1. Is the research ethical according to current criteria or, for recent studies, and is there evidence of ethical approval by an appropriate body? | X | □ | □ | □ |
| 1. Do the conclusions drawn in the research report flow from the analysis, or interpretation, of the data? | X | □ | □ | □ |

Overall appraisal: Include X Exclude □ Seek further info □

Comments (Including reason for exclusion): The authors situate their approach in constructivist theory but do not explicitly locate themselves culturally or personally in relation to the research. Some mention is made of reflexivity (memoing, bracketing assumptions), but the authors do not deeply reflect on their own positionality or how they may have influenced the findings.

| **RoB Assessor:** SMR | | **Date of Appraisal:** 28/8/25 | | **Record Number:** 169 Ref # 33 | | | | |
| --- | --- | --- | --- | --- | --- | --- | --- | --- |
| **Study Author:** Lyons, K., Lobczowski, N., Greene, J., Whitley, J. & McLaughlin, J. | | **Study Title:** Using a design-based research approach to develop and study a web-based tool to support collaborative learning | | **Study Year:** 2021 | | | | |
|  | |  | |  | | | | |
| **Internal Validity** | | | **Choice - Comments/Justification** | | **Yes** | **No** | **Unclear** | **N/A** |
| **Bias related to temporal precedence** | | | | | | | | |
| **1** | **Is it clear in the study what is the “cause” and what is the “effect” (i.e. there is no confusion about which variable comes first)?** | | Cause (intervention): The development and iterative refinement of a web-based tool—Collabucate—designed to foster social regulation of learning in collaborative contexts.  Effect (outcomes): How well social regulation processes were supported, as evaluated through log data, student ratings, and focus group feedback. The design of Collabucate precedes and is intended to influence collaborative behaviors | |  |  |  |  |
| **Bias related to selection and allocation** | | | | | | | | |
| **2** | **Was there a control group?** | | The authors applied a classic design based research (DBR) methodology: iterative design–implementation cycles with no separate control group. Instead, refinement occurs through two successive cycles within the same educational context | |  |  |  |  |
| **Bias related to confounding factors** | | | | | | | | |
| **3** | **Were participants included in any comparisons similar?** | | The participants in both cycles were Doctor of Pharmacy students engaging with Collabucate in their collaborative coursework. They are presumably comparable, as they represent the same student population across cycles | |  |  |  |  |
| **Bias related to administration of intervention/exposure** | | | | | | | | |
| **4** | **Were the participants included in any comparisons receiving similar treatment/care, other than the exposure or intervention of interest?** | | Aside from enhancements introduced to Collabucate between cycles, participants experienced similar contexts: similar tasks, same tool environment (with iterative changes), and comparable instructional settings. This consistency ensures other conditions remained stable. | |  |  |  |  |

| **Bias related to assessment, detection and measurement of the outcome** | | | | | | |
| --- | --- | --- | --- | --- | --- | --- |
| **5** | **Were there multiple measurements of the outcome, both pre and post the intervention/exposure?** | Instead of pre-/post-measurements, outcomes were assessed across two DBR cycles, capturing changes in tool usage and perceptions over iterations. This is consistent with DBR methodology but does not involve distinct pre-intervention baseline measures | **Yes** | **No** | **Unclear** | **N/A** |
|  | **Outcome 1** | Personal Ratings |  |  |  |  |
|  | **Outcome 2** | Challenges |  |  |  |  |
|  | **Outcome 3** | Strategies |  |  |  |  |
|  |  |  |  |  |  |  |
| **6** | **Were the outcomes of participants included in any comparisons measured in the same way?** | Across both cycles, the evaluation relied on the same types of data: system log data, student ratings, and focus group feedback. These instruments were consistently applied to assess tool impact and inform design changes | **Yes** | **No** | **Unclear** | **N/A** |
|  | **Outcome 1** | Personal Ratings |  |  |  |  |
|  | **Outcome 2** | Challenges |  |  |  |  |
|  | **Outcome 7** | Strategies |  |  |  |  |
|  |  |  |  |  |  |  |
| **7** | **Were outcomes measured in a reliable way?** |  | **Yes** | **No** | **Unclear** | **N/A** |
|  | **Outcome 1** | Personal Ratings |  |  |  |  |
|  | **Outcome 2** | Challenges |  |  |  |  |
|  | **Outcome 3** | Strategies |  |  |  |  |
|  | **Outcome 7** |  |  |  |  |  |

| **Bias related to participant retention** | | | | | | | | | | | | | | | |
| --- | --- | --- | --- | --- | --- | --- | --- | --- | --- | --- | --- | --- | --- | --- | --- |
| **8** | **Was follow-up complete and if not, were differences between groups in terms of their follow-up adequately described and analyzed?** | | | | | |  | |  | | | | | | |
|  | **Outcome 1** | | | | | | Personal Ratings | | **Yes** | **No** | | **Unclear** | | **N/A** | |
|  |  | Result 1 | | | | | Table 2 | |  |  |  | | |  | |
|  |  | Result 2 | | | | | Figure 1 | |  |  |  | | |  | |
|  |  | Result 3 | | | | |  | |  |  |  | | |  | |
|  | **Outcome 2** | | | | | | Challenges | | **Yes** | **No** | **Unclear** | | | **N/A** | |
|  |  | Result 1 | | | | | Table 5 | |  |  |  | | |  | |
|  |  | Result 2 | | | | | Table 6 | |  |  |  | | |  | |
|  |  | Result 3 | | | | | Table 7 | |  |  |  | | |  | |
|  | **Outcome 3** | | | | | | Strategies | | **Yes** | **No** | **Unclear** | | | **N/A** | |
|  |  | Result 1 | | | | | Table 8 | |  |  |  | | |  | |
|  |  | Result 3 | | | | |  | |  |  |  | | |  | |
|  |  |  | | | | | | |  |  |  | | |  | |
|  | **Statistical Conclusion Validity** | | | | | | | |  |  |  | | |  | |
| **9** | **Was appropriate statistical analysis used?** | | | | | | |  |  | | | | | | |
|  | **Outcome 1** | | |  | | | | Personal Ratings | **Yes** | **No** | **Unclear** | | | **N/A** | |
|  |  | Result 1 | | | | | | Table 2 |  |  |  | | |  | |
|  |  | Result 2 | | | | | | Figure 1 |  |  |  | | |  | |
|  |  | Result 3 | | | | | |  |  |  |  | | |  | |
|  | **Outcome 2** | | |  | | | | Challenges | **Yes** | **No** | **Unclear** | | | **N/A** | |
|  |  | Result 1 | | | | | | Table 5 |  |  |  | | |  | |
|  |  | Result 2 | | | | | | Table 6 |  |  |  | | |  | |
|  |  | Result 3 | | | | | | Table 7 |  |  |  | | |  | |
|  | **Outcome 3** | | |  | | | | Strategies | **Yes** | **No** | **Unclear** | | | **N/A** | |
|  |  | Result 1 | | | | | | Table 8 |  |  |  | | |  | |
|  |  | Result 3 | | | | | |  |  |  | | |  | |  |
|  | | | | | | | | | | | | | | | |
| **Overall appraisal:** | | | **Include:** | | **Exclude:** | **Seek Further Info:** | | | | | | | | | |
| **Comments:** Lyons et al. (2021) executed a design-based research project developing Collabucate, a web tool to support social regulation in collaborative learning. Their approach actively iterates the design informed by mixed-data sources—log analytics, student feedback, and focus groups. While the study lacks a traditional control group or pre-testing, its methodology aligns with DBR conventions, offering practical insights and tool refinements grounded in real-world use. Measurements appear reasonable, though details about rating reliability and qualitative analysis rigor aren't available from abstracts. Overall, the study is methodologically coherent for its purpose and contributes meaningfully to knowledge on tool-supported collaborative learning design. | | | | | | | | | | | | | | | |

© JBI, 2022. All rights reserved. JBI grants use of these

 tools for research purposes only. All other enquiries

 should be sent to[jbisynthesis@adelaide.edu.au](mailto:jbisynthesis@adelaide.edu.au)

JBI Critical Appraisal Checklist for
Qualitative Research

Reviewer: S. Rutherford Date: 26/08/2025

Author: Lyons, K., Lobczowski, N., Greene, J., Whitley, J. & McLaughlin, J. (2021) Using a design-based research approach to develop and study a web-based tool to support collaborative learning, *Comput Educ,* **161**, 104064

Year: 2021 Record Number: 169 Ref #33

|  | Yes | No | Unclear | Not applicable |
| --- | --- | --- | --- | --- |
| 1. Is there congruity between the stated philosophical perspective and the research methodology? | x | □ | □ | □ |
| 1. Is there congruity between the research methodology and the research question or objectives? | x | □ | □ | □ |
| 1. Is there congruity between the research methodology and the methods used to collect data? | x | □ | □ | □ |
| 1. Is there congruity between the research methodology and the representation and analysis of data? | x | □ | □ | □ |
| 1. Is there congruity between the research methodology and the interpretation of results? | x | □ | □ | □ |
| 1. Is there a statement locating the researcher culturally or theoretically? | x | □ | □ | □ |
| 1. Is the influence of the researcher on the research, and vice- versa, addressed? | x | □ | □ | □ |
| 1. Are participants, and their voices, adequately represented? | □ | x | □ | □ |
| 1. Is the research ethical according to current criteria or, for recent studies, and is there evidence of ethical approval by an appropriate body? | □ | x | □ | □ |
| 1. Do the conclusions drawn in the research report flow from the analysis, or interpretation, of the data? | x | □ | □ | □ |

Overall appraisal: Include x Exclude □ Seek further info □

Comments (Including reason for exclusion): Mixed Methods, so some qualitative approaches less relevant. Conclusions do align with the data, but qualitative results are more for support than proving hypotheses.

JBI Critical Appraisal Checklist for
Qualitative Research

Reviewer: S. Rutherford Date: 26/08/2025

Author: Shana, Z. (2009) Learning with Technology: Using Discussion Forums to Augment a Traditional-Style Class, *Educ Technol Soc*, 12, 214-228.

Year: 2009 Record Number: 181 Ref #34

|  | Yes | No | Unclear | Not applicable |
| --- | --- | --- | --- | --- |
| 1. Is there congruity between the stated philosophical perspective and the research methodology? | x | □ | □ | □ |
| 1. Is there congruity between the research methodology and the research question or objectives? | x | □ | □ | □ |
| 1. Is there congruity between the research methodology and the methods used to collect data? | x | □ | □ | □ |
| 1. Is there congruity between the research methodology and the representation and analysis of data? | x | □ | □ | □ |
| 1. Is there congruity between the research methodology and the interpretation of results? | x | □ | □ | □ |
| 1. Is there a statement locating the researcher culturally or theoretically? | x | □ | □ | □ |
| 1. Is the influence of the researcher on the research, and vice- versa, addressed? | □ | x | □ | □ |
| 1. Are participants, and their voices, adequately represented? | x | □ | □ | □ |
| 1. Is the research ethical according to current criteria or, for recent studies, and is there evidence of ethical approval by an appropriate body? | □ | x | □ | □ |
| 1. Do the conclusions drawn in the research report flow from the analysis, or interpretation, of the data? | x | □ | □ | □ |

Overall appraisal: Include x Exclude □ Seek further info □

Comments (Including reason for exclusion): A mix of quantitative and some qualitative work. The qualitative was subsidiary to the quantitative, so despite it being less than ideal, it was still not problematic. The research was undertaken in an ethical manner, it appears, but no ethics statement was included.

| **RoB Assessor:** SMR | | **Date of Appraisal:** 28/8/25 | | **Record Number:** 189 Ref #35 | | | | |
| --- | --- | --- | --- | --- | --- | --- | --- | --- |
| **Study Author:** Reynolds, P. A., Harper, J., Dunne, S., Cox, M. & Myint, Y. K. | | **Study Title:** Portable Digital Assistants (PDAs) in dentistry: Part II - Pilot study of PDA use in the dental clinic | | **Study Year:** 2007 | | | | |
|  | |  | |  | | | | |
| **Internal Validity** | | | **Choice - Comments/Justification** | | **Yes** | **No** | **Unclear** | **N/A** |
| **Bias related to temporal precedence** | | | | | | | | |
| **1** | **Is it clear in the study what is the “cause” and what is the “effect” (i.e. there is no confusion about which variable comes first)?** | | Cause (intervention): Use of PDAs (Portable Digital Assistants) by dental undergraduate students in a clinical setting, enabling access to Virtual Learning Environment (VLE) resources.  Effect (outcomes): Student attitudes, educational usage (e.g., viewing online materials, note-taking, diaries), technical feasibility, and broader perceptions of PDA integration.  The intervention clearly precedes and influences the reported outcomes. | |  |  |  |  |
| **Bias related to selection and allocation** | | | | | | | | |
| **2** | **Was there a control group?** | | The study involved 12 fourth-year undergraduate students, divided into two groups of six. They undertook a 12-week crossover trial, alternating between PDA use and non-use in the clinical environment. | |  |  |  |  |
| **Bias related to confounding factors** | | | | | | | | |
| **3** | **Were participants included in any comparisons similar?** | | All participants were fourth-year dental undergraduates within the same clinical program. The crossover design ensures each group experiences both conditions, supporting comparability. | |  |  |  |  |
| **Bias related to administration of intervention/exposure** | | | | | | | | |
| **4** | **Were the participants included in any comparisons receiving similar treatment/care, other than the exposure or intervention of interest?** | | Aside from the use or absence of PDAs, all students operated in the same Primary Dental Care clinic environment with identical curricular and clinical conditions. | |  |  |  |  |

| **Bias related to assessment, detection and measurement of the outcome** | | | | | | |
| --- | --- | --- | --- | --- | --- | --- |
| **5** | **Were there multiple measurements of the outcome, both pre and post the intervention/exposure?** | Outcomes were assessed using online questionnaires and focus group discussions, conducted during both intervention phases. This enables within-subject comparison between PDA use and non-use conditions, albeit not tied to a temporal pre/post benchmark | **Yes** | **No** | **Unclear** | **N/A** |
|  | **Outcome 1** | Percentage Visits |  |  |  |  |
|  | **Outcome 2** | Participant Perceptions |  |  |  |  |
|  | **Outcome 3** | Questionnaire |  |  |  |  |
|  |  |  |  |  |  |  |
| **6** | **Were the outcomes of participants included in any comparisons measured in the same way?** |  | **Yes** | **No** | **Unclear** | **N/A** |
|  | **Outcome 1** | Percentage Visits |  |  |  |  |
|  | **Outcome 2** | Participant Perceptions |  |  |  |  |
|  | **Outcome 3** | Questionnaire |  |  |  |  |
|  |  |  |  |  |  |  |
| **7** | **Were outcomes measured in a reliable way?** |  | **Yes** | **No** | **Unclear** | **N/A** |
|  | **Outcome 1** | Percentage Visits |  |  |  |  |
|  | **Outcome 2** | Participant Perceptions |  |  |  |  |
|  | **Outcome 3** | Questionnaire |  |  |  |  |

| **Bias related to participant retention** | | | | | | | | | | | | | | | |
| --- | --- | --- | --- | --- | --- | --- | --- | --- | --- | --- | --- | --- | --- | --- | --- |
| **8** | **Was follow-up complete and if not, were differences between groups in terms of their follow-up adequately described and analyzed?** | | | | | |  | |  | | | | | | |
|  | **Outcome 1** | | | | | | Percentage Visits | | **Yes** | **No** | | **Unclear** | | **N/A** | |
|  |  | Result 1 | | | | | Table 1 | |  |  |  | | |  | |
|  |  | Result 3 | | | | |  | |  |  |  | | |  | |
|  | **Outcome 2** | | | | | | Participant Perceptions | | **Yes** | **No** | **Unclear** | | | **N/A** | |
|  |  | Result 1 | | | | | Fig 3 | |  |  |  | | |  | |
|  |  | Result 3 | | | | |  | |  |  |  | | |  | |
|  | **Outcome 3** | | | | | | Questionnaire | | **Yes** | **No** | **Unclear** | | | **N/A** | |
|  |  | Result 1 | | | | | Appendix 1 | |  |  |  | | |  | |
|  |  | Result 2 | | | | | Appendix 2 | |  |  |  | | |  | |
|  |  | Result 3 | | | | |  | |  |  |  | | |  | |
|  |  |  | | | | | | |  |  |  | | |  | |
|  | **Statistical Conclusion Validity** | | | | | | | |  |  |  | | |  | |
| **9** | **Was appropriate statistical analysis used?** | | | | | | |  |  | | | | | | |
|  | **Outcome 1** | | |  | | | | Percentage Visits | **Yes** | **No** | **Unclear** | | | **N/A** | |
|  |  | Result 1 | | | | | | Table 1 |  |  |  | | |  | |
|  |  | Result 3 | | | | | |  |  |  |  | | |  | |
|  | **Outcome 2** | | |  | | | | Participant Perceptions | **Yes** | **No** | **Unclear** | | | **N/A** | |
|  |  | Result 1 | | | | | | Fig 3 |  |  |  | | |  | |
|  |  | Result 3 | | | | | |  |  |  |  | | |  | |
|  | **Outcome 3** | | |  | | | | Questionnaire | **Yes** | **No** | **Unclear** | | | **N/A** | |
|  |  | Result 1 | | | | | | Appendix 1 |  |  |  | | |  | |
|  |  | Result 2 | | | | | | Appendix 2 |  |  | | |  | |  |
|  | | | | | | | | | | | | | | | |
| **Overall appraisal:** | | | **Include:** | | **Exclude:** | **Seek Further Info:** | | | | | | | | | |
| **Comments:** A well-structured crossover pilot trial assessing PDA feasibility and educational impact in a real-world dental clinic setting. Cause and effect are clearly delineated—with strong internal control via crossover. Participants were similar and treated consistently. Measurements, though subjective, were systematically applied, and follow-up was complete. Analysis is appropriately descriptive for a feasibility study, aimed at informing future broader implementation. | | | | | | | | | | | | | | | |

© JBI, 2022. All rights reserved. JBI grants use of these

 tools for research purposes only. All other enquiries

 should be sent to[jbisynthesis@adelaide.edu.au](mailto:jbisynthesis@adelaide.edu.au)

| **RoB Assessor:** SMR | | **Date of Appraisal:** 4/9/25 | | **Record Number:** 195 Ref #36 | | | | |
| --- | --- | --- | --- | --- | --- | --- | --- | --- |
| **Study Author:** Van Es, S. L., Kumar, R. K., Pryor, W. M., Salisbury, E. L. & Velan, G. M. | | **Study Title:** Cytopathology whole slide images and adaptive tutorials for senior medical students: a randomized crossover trial | | **Study Year:** 2016 | | | | |
|  | |  | |  | | | | |
| **Internal Validity** | | | **Choice - Comments/Justification** | | **Yes** | **No** | **Unclear** | **N/A** |
| **Bias related to temporal precedence** | | | | | | | | |
| **1** | **Is it clear in the study what is the “cause” and what is the “effect” (i.e. there is no confusion about which variable comes first)?** | | Cause (intervention): Learning cytopathology using whole slide images (WSI) combined with virtual microscopy adaptive tutorials (VMATs).  Effect (outcomes): Student learning outcomes in diagnosis and feature identification, measured through assessments, along with user perceptions of efficiency, interactivity, and equity.  The randomized crossover design cleanly establishes that exposure to WSI/VMATs precedes and is intended to impact these outcomes. | |  |  |  |  |
| **Bias related to selection and allocation** | | | | | | | | |
| **2** | **Was there a control group?** | | Students were randomized into two groups; each alternately experienced either the intervention (WSI + VMATs) or comparator (existing digital resources like e textbooks and online atlases) across multiple phases. This design serves as an internal control. | |  |  |  |  |
| **Bias related to confounding factors** | | | | | | | | |
| **3** | **Were participants included in any comparisons similar?** | | Participants were senior Year 5 and Year 6 medical students from the same institution, randomly assigned and balanced across groups. This ensures qualitative similarity and comparability between groups | |  |  |  |  |
| **Bias related to administration of intervention/exposure** | | | | | | | | |
| **4** | **Were the participants included in any comparisons receiving similar treatment/care, other than the exposure or intervention of interest?** | | All participants experienced the same clinical context, subject matter, study duration, and assessment schedule. The only systematic difference was whether they used WSI + VMATs or traditional digital resources during a given phase | |  |  |  |  |

| **Bias related to assessment, detection and measurement of the outcome** | | | | | | |
| --- | --- | --- | --- | --- | --- | --- |
| **5** | **Were there multiple measurements of the outcome, both pre and post the intervention/exposure?** | Outcomes were assessed after each learning phase, allowing within-subject comparisons between intervention and control conditions. While not classic pre/post, the crossover design functions similarly by alternately measuring performance under each condition | **Yes** | **No** | **Unclear** | **N/A** |
|  | **Outcome 1** | Assessment |  |  |  |  |
|  | **Outcome 2** | Questionnaire ratings |  |  |  |  |
|  | **Outcome 3** | Open-ended questionnaire responses |  |  |  |  |
|  |  |  |  |  |  |  |
| **6** | **Were the outcomes of participants included in any comparisons measured in the same way?** |  | **Yes** | **No** | **Unclear** | **N/A** |
|  | **Outcome 1** | Assessment |  |  |  |  |
|  | **Outcome 2** | Questionnaire ratings |  |  |  |  |
|  | **Outcome 3** | Open-ended questionnaire responses |  |  |  |  |
|  |  |  |  |  |  |  |
| **7** | **Were outcomes measured in a reliable way?** |  | **Yes** | **No** | **Unclear** | **N/A** |
|  | **Outcome 1** | Assessment |  |  |  |  |
|  | **Outcome 2** | Questionnaire ratings |  |  |  |  |
|  | **Outcome 3** | Open-ended questionnaire responses |  |  |  |  |

| **Bias related to participant retention** | | | | | | | | | | | | | | | |
| --- | --- | --- | --- | --- | --- | --- | --- | --- | --- | --- | --- | --- | --- | --- | --- |
| **8** | **Was follow-up complete and if not, were differences between groups in terms of their follow-up adequately described and analyzed?** | | | | | |  | |  | | | | | | |
|  | **Outcome 1** | | | | | | Assessment | | **Yes** | **No** | | **Unclear** | | **N/A** | |
|  |  | Result 1 | | | | | Fig 3 | |  |  |  | | |  | |
|  |  | Result 3 | | | | |  | |  |  |  | | |  | |
|  | **Outcome 2** | | | | | | Questionnaire ratings | | **Yes** | **No** | **Unclear** | | | **N/A** | |
|  |  | Result 1 | | | | | Fig 4 | |  |  |  | | |  | |
|  |  | Result 2 | | | | |  | |  |  |  | | |  | |
|  |  | Result 3 | | | | |  | |  |  |  | | |  | |
|  | **Outcome 3** | | | | | | Open-ended questionnaire responses | | **Yes** | **No** | **Unclear** | | | **N/A** | |
|  |  | Result 1 | | | | | Table 2 | |  |  |  | | |  | |
|  |  | Result 2 | | | | | Table 3 | |  |  |  | | |  | |
|  |  |  | | | | | | |  |  |  | | |  | |
|  | **Statistical Conclusion Validity** | | | | | | | |  |  |  | | |  | |
| **9** | **Was appropriate statistical analysis used?** | | | | | | |  |  | | | | | | |
|  | **Outcome 1** | | |  | | | | Assessment | **Yes** | **No** | **Unclear** | | | **N/A** | |
|  |  | Result 1 | | | | | | Fig 3 |  |  |  | | |  | |
|  |  | Result 3 | | | | | |  |  |  |  | | |  | |
|  | **Outcome 2** | | |  | | | | Questionnaire ratings | **Yes** | **No** | **Unclear** | | | **N/A** | |
|  |  | Result 1 | | | | | | Fig 4 |  |  |  | | |  | |
|  |  | Result 3 | | | | | |  |  |  |  | | |  | |
|  | **Outcome 3** | | |  | | | | Open-ended questionnaire responses | **Yes** | **No** | **Unclear** | | | **N/A** | |
|  |  | Result 1 | | | | | | Table 2 |  |  |  | | |  | |
|  |  | Result 3 | | | | | |  |  |  | | |  | |  |
|  | | | | | | | | | | | | | | | |
| **Overall appraisal:** | | | **Include:** | | **Exclude:** | **Seek Further Info:** | | | | | | | | | |
| **Comments:** The study effectively compares traditional digital learning resources with adaptive virtual microscopy using WSI + VMATs. The study clearly distinguishes cause and effect, uses comparable participants and environments, applies consistent measurement strategies, and engages robust statistical analysis. While there's modest attrition, it’s acknowledged and does not invalidate the findings. Results show that VMATs are as effective—or in certain aspects, superior—to traditional methods, with strong user satisfaction in interactivity and feedback. | | | | | | | | | | | | | | | |

© JBI, 2022. All rights reserved. JBI grants use of these

 tools for research purposes only. All other enquiries

 should be sent to[jbisynthesis@adelaide.edu.au](mailto:jbisynthesis@adelaide.edu.au)

| **RoB Assessor:** SMR | | **Date of Appraisal:** 28/8/25 | | **Record Number:** 203 Ref # 37 | | | | |
| --- | --- | --- | --- | --- | --- | --- | --- | --- |
| **Study Author:** Ahmed, M. & Hasegawa, S. | | **Study Title:** The effects of a new virtual learning platform on improving student skills in designing and producing online virtual laboratories | | **Study Year:** 2019 | | | | |
|  | |  | |  | | | | |
| **Internal Validity** | | | **Choice - Comments/Justification** | | **Yes** | **No** | **Unclear** | **N/A** |
| **Bias related to temporal precedence** | | | | | | | | |
| **1** | **Is it clear in the study what is the “cause” and what is the “effect” (i.e. there is no confusion about which variable comes first)?** | | Cause (intervention): The introduction of a novel Virtual Learning Platform (VLP), including instructor/student modes, design & creator tools, templates, and course content for creating Online Virtual Laboratories (OVLs).  Effect (outcomes): Students’ improved knowledge, practical skills, and product outputs in designing and producing OVLs, along with positive usability and attitudes. | |  |  |  |  |
| **Bias related to selection and allocation** | | | | | | | | |
| **2** | **Was there a control group?** | | The study used a one-group pretest–posttest design with no separate control or comparison group | |  |  |  |  |
| **Bias related to confounding factors** | | | | | | | | |
| **3** | **Were participants included in any comparisons similar?** | | All participants were 30 Year 4 undergraduate students majoring in Educational Technology, sharing similar background and academic level | |  |  |  |  |
| **Bias related to administration of intervention/exposure** | | | | | | | | |
| **4** | **Were the participants included in any comparisons receiving similar treatment/care, other than the exposure or intervention of interest?** | | All participants underwent the same learning conditions: orientation, pretest, guided use of the VLP in computer labs, and posttest. Instructor support and system access were consistent across the cohort | |  |  |  |  |

| **Bias related to assessment, detection and measurement of the outcome** | | | | | | |
| --- | --- | --- | --- | --- | --- | --- |
| **5** | **Were there multiple measurements of the outcome, both pre and post the intervention/exposure?** |  | **Yes** | **No** | **Unclear** | **N/A** |
|  | **Outcome 1** | Achievement test |  |  |  |  |
|  | **Outcome 2** | Performance observation card |  |  |  |  |
|  | **Outcome 3** | Product evaluation card |  |  |  |  |
|  | **Outcome 4** | Usability questionnaire (user perceptions) |  |  |  |  |
|  |  |  |  |  |  |  |
| **6** | **Were the outcomes of participants included in any comparisons measured in the same way?** |  | **Yes** | **No** | **Unclear** | **N/A** |
|  | **Outcome 1** | Achievement test |  |  |  |  |
|  | **Outcome 2** | Performance observation card |  |  |  |  |
|  | **Outcome 3** | Product evaluation card |  |  |  |  |
|  | **Outcome 4** | Usability questionnaire (user perceptions) |  |  |  |  |
|  |  |  |  |  |  |  |
| **7** | **Were outcomes measured in a reliable way?** |  | **Yes** | **No** | **Unclear** | **N/A** |
|  | **Outcome 1** | Achievement test |  |  |  |  |
|  | **Outcome 2** | Performance observation card |  |  |  |  |
|  | **Outcome 3** | Product evaluation card |  |  |  |  |
|  | **Outcome 4** | Usability questionnaire (user perceptions) |  |  |  |  |

| **Bias related to participant retention** | | | | | | | | | | | | | | | |
| --- | --- | --- | --- | --- | --- | --- | --- | --- | --- | --- | --- | --- | --- | --- | --- |
| **8** | **Was follow-up complete and if not, were differences between groups in terms of their follow-up adequately described and analyzed?** | | | | | |  | |  | | | | | | |
|  | **Outcome 1** | | | | | | Achievement test | | **Yes** | **No** | | **Unclear** | | **N/A** | |
|  |  | Result 1 | | | | | Table 2 | |  |  |  | | |  | |
|  |  | Result 2 | | | | | Fig 5 | |  |  |  | | |  | |
|  | **Outcome 2** | | | | | | Performance observation card | | **Yes** | **No** | **Unclear** | | | **N/A** | |
|  |  | Result 1 | | | | | Table 3 | |  |  |  | | |  | |
|  |  | Result 2 | | | | |  | |  |  |  | | |  | |
|  | **Outcome 3** | | | | | | Product evaluation card | | **Yes** | **No** | **Unclear** | | | **N/A** | |
|  |  | Result 1 | | | | | Table 4 | |  |  |  | | |  | |
|  |  | Result 2 | | | | | Table 5 | |  |  |  | | |  | |
|  | **Outcome 4** | | | | | | Usability questionnaire (user perceptions) | | **Yes** | **No** | **Unclear** | | | **N/A** | |
|  |  | Result 1 | | | | | Table 6 | |  |  |  | | |  | |
|  |  |  | | | | | | |  |  |  | | |  | |
|  | **Statistical Conclusion Validity** | | | | | | | |  |  |  | | |  | |
| **9** | **Was appropriate statistical analysis used?** | | | | | | |  |  | | | | | | |
|  | **Outcome 1** | | |  | | | | Achievement test | **Yes** | **No** | **Unclear** | | | **N/A** | |
|  |  | Result 1 | | | | | | Table 2 |  |  |  | | |  | |
|  |  | Result 2 | | | | | | Fig 5 |  |  |  | | |  | |
|  | **Outcome 2** | | |  | | | | Performance observation card | **Yes** | **No** | **Unclear** | | | **N/A** | |
|  |  | Result 1 | | | | | | Table 3 |  |  |  | | |  | |
|  | **Outcome 3** | | |  | | | | Product evaluation card | **Yes** | **No** | **Unclear** | | | **N/A** | |
|  |  | Result 1 | | | | | | Table 4 |  |  |  | | |  | |
|  |  | Result 2 | | | | | | Table 5 |  |  |  | | |  | |
|  | **Outcome 4** | | | | | | | Usability questionnaire (user perceptions) | Yes | **No** | | | **Unclear** | | **N/A** |
|  |  | Result 1 | | | | | | Table 6 |  |  | | |  | |  |
|  |  | Result 3 | | | | | |  |  |  | | |  | |  |
|  | | | | | | | | | | | | | | | |
| **Overall appraisal:** | | | **Include:** | | **Exclude:** | **Seek Further Info:** | | | | | | | | | |
| **Comments:** The intervention (VLP) and outcomes are clearly defined, measurements are comprehensive and reliable, statistical analysis is appropriate and informative, and the study benefits from full participant follow-through.  While the absence of a control group means results are less generalizable compared to randomized or comparative designs, the large effect sizes and positive usability findings strongly suggest the VLP’s effectiveness. The study sets a solid foundation for future research—ideally incorporating control conditions, larger samples, and long-term assessment. | | | | | | | | | | | | | | | |

© JBI, 2022. All rights reserved. JBI grants use of these

 tools for research purposes only. All other enquiries

 should be sent to[jbisynthesis@adelaide.edu.au](mailto:jbisynthesis@adelaide.edu.au)

| **RoB Assessor:** SMR | | | **Date of Appraisal:** 4/9/25 | | | **Record Number:** 15 Ref # 39 | | | | |  |
| --- | --- | --- | --- | --- | --- | --- | --- | --- | --- | --- | --- |
| **Study Author:** Estriégana, R., Medina-Merodio, J.-A., Robina-Ramírez, R. & Barchino, R. | | | **Study Title:** Analysis of Cooperative Skills Development through Relational Coordination in a Gamified Online Learning Environment | | | **Study Year:** 2021 | | | | |  |
|  | |  | | |  | | | | | | |
| **Internal Validity** | | | | **Choice - Comments/Justification** | | | **Yes** | **No** | **Unclear** | **N/A** | |
| **Bias related to temporal precedence** | | | | | | | | | | | |
| **1** | **Is it clear in the study what is the “cause” and what is the “effect” (i.e. there is no confusion about which variable comes first)?** | | | Cause (intervention): A gamified online learning environment (OLE) incorporating virtual labs, interactive tasks, videos, and game-based methodology.  Effect (outcomes): Enhanced relational coordination (i.e., improved communication and relationships among students), leading to strengthened cooperative competence | | |  |  |  |  | |
| **Bias related to selection and allocation** | | | | | | | | | | | |
| **2** | **Was there a control group?** | | | The study surveyed a single cohort using the OLE and examined relationships through structural equation modeling (SEM). No comparison or non-intervention group was included | | |  |  |  |  | |
| **Bias related to confounding factors** | | | | | | | | | | | |
| **3** | **Were participants included in any comparisons similar?** | | | The participants were 289 students enrolled in core Computer Engineering and Computer Science Engineering courses. Being from the same subject and context, participant similarity is maintained | | |  |  |  |  | |
| **Bias related to administration of intervention/exposure** | | | | | | | | | | | |
| **4** | **Were the participants included in any comparisons receiving similar treatment/care, other than the exposure or intervention of interest?** | | | All students experienced the same gamified learning environment and resources. There were no alternative treatments or differential conditions apart from using the OLE | | |  |  |  |  | |

| **Bias related to assessment, detection and measurement of the outcome** | | | | | | |
| --- | --- | --- | --- | --- | --- | --- |
| **5** | **Were there multiple measurements of the outcome, both pre and post the intervention/exposure?** | Instead of pre/post measurements, the study uses cross-sectional data collected via a survey at one point in time, capturing perceptions and competence levels. Outcomes are inferred via SEM rather than longitudinal change | **Yes** | **No** | **Unclear** | **N/A** |
|  | **Outcome 1** | Questionnaire |  |  |  |  |
|  | **Outcome 2** | Structural Model Analysis |  |  |  |  |
|  | **Outcome 3** | Analysis of Efficiency and Satisfaction |  |  |  |  |
|  | **Outcome 4** | OLE satisfaction survey. |  |  |  |  |
|  | **Outcome 7** |  |  |  |  |  |
|  |  |  |  |  |  |  |
| **6** | **Were the outcomes of participants included in any comparisons measured in the same way?** |  | **Yes** | **No** | **Unclear** | **N/A** |
|  | **Outcome 1** | Questionnaire |  |  |  |  |
|  | **Outcome 2** | Structural Model Analysis |  |  |  |  |
|  | **Outcome 3** | Analysis of Efficiency and Satisfaction |  |  |  |  |
|  | **Outcome 4** | OLE satisfaction survey. |  |  |  |  |
|  |  |  |  |  |  |  |
| **7** | **Were outcomes measured in a reliable way?** |  | **Yes** | **No** | **Unclear** | **N/A** |
|  | **Outcome 1** | Questionnaire |  |  |  |  |
|  | **Outcome 2** | Structural Model Analysis |  |  |  |  |
|  | **Outcome 3** | Analysis of Efficiency and Satisfaction |  |  |  |  |
|  | **Outcome 4** | OLE satisfaction survey. |  |  |  |  |

| **Bias related to participant retention** | | | | | | | | | | | | | | | |
| --- | --- | --- | --- | --- | --- | --- | --- | --- | --- | --- | --- | --- | --- | --- | --- |
| **8** | **Was follow-up complete and if not, were differences between groups in terms of their follow-up adequately described and analyzed?** | | | | | |  | |  | | | | | | |
|  | **Outcome 1** | | | | | | Questionnaire | | **Yes** | **No** | | **Unclear** | | **N/A** | |
|  |  | Result 1 | | | | | Table 1 | |  |  |  | | |  | |
|  |  | Result 2 | | | | | Table 2 | |  |  |  | | |  | |
|  |  | Result 3 | | | | | Table 3 | |  |  |  | | |  | |
|  | **Outcome 2** | | | | | | Structural Model Analysis | | **Yes** | **No** | **Unclear** | | | **N/A** | |
|  |  | Result 1 | | | | | Tables 4 & 5 | |  |  |  | | |  | |
|  |  | Result 2 | | | | | Figures 3 & 4 | |  |  |  | | |  | |
|  |  | Result 3 | | | | | Table 6 | |  |  |  | | |  | |
|  | **Outcome 3** | | | | | | Analysis of Efficiency and Satisfaction | | **Yes** | **No** | **Unclear** | | | **N/A** | |
|  |  | Result 1 | | | | | Table 7 | |  |  |  | | |  | |
|  | **Outcome 4** | | | | | | OLE satisfaction survey. | | **Yes** | **No** | **Unclear** | | | **N/A** | |
|  |  | Result 1 | | | | | Table 8 | |  |  |  | | |  | |
|  |  |  | | | | | | |  |  |  | | |  | |
|  | **Statistical Conclusion Validity** | | | | | | | |  |  |  | | |  | |
| **9** | **Was appropriate statistical analysis used?** | | | | | | |  |  | | | | | | |
|  | **Outcome 1** | | |  | | | | Questionnaire | **Yes** | **No** | **Unclear** | | | **N/A** | |
|  |  | Result 1 | | | | | | Table 1 |  |  |  | | |  | |
|  |  | Result 2 | | | | | | Table 2 |  |  |  | | |  | |
|  |  | Result 3 | | | | | | Table 3 |  |  |  | | |  | |
|  | **Outcome 2** | | |  | | | | Structural Model Analysis | **Yes** | **No** | **Unclear** | | | **N/A** | |
|  |  | Result 1 | | | | | | Tables 4 & 5 |  |  |  | | |  | |
|  |  | Result 2 | | | | | | Figures 3 & 4 |  |  |  | | |  | |
|  |  | Result 3 | | | | | | Table 6 |  |  |  | | |  | |
|  | **Outcome 3** | | |  | | | | Analysis of Efficiency and Satisfaction | **Yes** | **No** | **Unclear** | | | **N/A** | |
|  |  | Result 1 | | | | | | Table 7 |  |  |  | | |  | |
|  | **Outcome 4** | | | | | | | OLE satisfaction survey | **Yes**. | **No** | | | **Unclear** | | **N/A** |
|  |  | Result 1 | | | | | | Table 8 |  |  | | |  | |  |
|  | | | | | | | | | | | | | | | |
| **Overall appraisal:** | | | **Include:** | | **Exclude:** | **Seek Further Info:** | | | | | | | | | |
| **Comments:** A rigorous cross-sectional evaluation of a gamified online learning environment’s impact on relational coordination and cooperative competence. While lacking a longitudinal or control design, the sample size is substantial (n = 289), and the use of SEM ensures statistical sophistication. The constructs showed good reliability and validity, lending confidence to the findings that the OLE positively influences both communication/relationships and cooperative competence. | | | | | | | | | | | | | | | |

© JBI, 2022. All rights reserved. JBI grants use of these

 tools for research purposes only. All other enquiries

 should be sent to[jbisynthesis@adelaide.edu.au](mailto:jbisynthesis@adelaide.edu.au)

| **RoB Assessor:** SMR | | **Date of Appraisal:** 28/8/25 | | **Record Number:** 168 Ref # 40 | | | | |
| --- | --- | --- | --- | --- | --- | --- | --- | --- |
| **Study Author:** Lopez-Pernas, S., Gordillo, A., Barra, E. & Quemada, J. | | **Study Title:** Escapp: A Web Platform for Conducting Educational Escape Rooms | | **Study Year:** 2021 | | | | |
|  | |  | |  | | | | |
| **Internal Validity** | | | **Choice - Comments/Justification** | | **Yes** | **No** | **Unclear** | **N/A** |
| **Bias related to temporal precedence** | | | | | | | | |
| **1** | **Is it clear in the study what is the “cause” and what is the “effect” (i.e. there is no confusion about which variable comes first)?** | | Cause (intervention): Introduction of Escapp, a web-based platform designed to conduct both face-to-face and remote educational escape rooms by automating registration, team formation, hint delivery, progress monitoring, grading, and other logistical features.  Effect (outcomes): Enhanced usability, engagement, and overall effectiveness in conducting educational escape rooms, evidenced by high levels of student interaction and positive perceptions of platform utility. | |  |  |  |  |
| **Bias related to selection and allocation** | | | | | | | | |
| **2** | **Was there a control group?** | | The study comprises three case studies using Escapp itself (one face-to-face; two remote) with no comparative control condition (e.g., escape rooms without Escapp). Evaluations rely on student feedback and system log data, but there's no control group for comparison | |  |  |  |  |
| **Bias related to confounding factors** | | | | | | | | |
| **3** | **Were participants included in any comparisons similar?** | | The platform was used in three different higher-education courses, encompassing over 400 students—though specific course domains and participant demographics vary across case studies, all were higher-education learners participating in Escapp-facilitated escape rooms | |  |  |  |  |
| **Bias related to administration of intervention/exposure** | | | | | | | | |
| **4** | **Were the participants included in any comparisons receiving similar treatment/care, other than the exposure or intervention of interest?** | | In all three case studies, students participated through the same platform, experiencing core common features (team formation, hints, leaderboards, progress monitoring). Core platform functionality remained consistent across deployments, even though one was face-to-face and the others remote | |  |  |  |  |

| **Bias related to assessment, detection and measurement of the outcome** | | | | | | |
| --- | --- | --- | --- | --- | --- | --- |
| **5** | **Were there multiple measurements of the outcome, both pre and post the intervention/exposure?** | The study uses post-activity student questionnaires and system log data from within the escape room events (during use), but lacks pre-activity or baseline measurements for comparison (e.g., prior usability or engagement levels before Escapp). The measurement is singular and post-completion | **Yes** | **No** | **Unclear** | **N/A** |
|  | **Outcome 1** | Usage Statistics |  |  |  |  |
|  | **Outcome 2** | Student questionnaire |  |  |  |  |
|  |  |  |  |  |  |  |
| **6** | **Were the outcomes of participants included in any comparisons measured in the same way?** | All students, across all case studies, completed the same questionnaire assessing usefulness, usability, engagement, and platform features (with means and standard deviations reported), and all their interactions were recorded uniformly via the platform logs | **Yes** | **No** | **Unclear** | **N/A** |
|  | **Outcome 1** | Usage Statistics |  |  |  |  |
|  | **Outcome 2** | Student questionnaire |  |  |  |  |
|  |  |  |  |  |  |  |
| **7** | **Were outcomes measured in a reliable way?** | Student responses were collected through structured questionnaires (quantified via mean and SD scores), albeit details on psychometric validation are not provided.  System log data offers highly reliable, objective evidence of student engagement (e.g., interaction frequency, workload peaks). While formal reliability metrics are not detailed, these instruments appear consistent and suitable | **Yes** | **No** | **Unclear** | **N/A** |
|  | **Outcome 1** | Usage Statistics |  |  |  |  |
|  | **Outcome 2** | Student questionnaire |  |  |  |  |

| **Bias related to participant retention** | | | | | | | | | | | | | | | |
| --- | --- | --- | --- | --- | --- | --- | --- | --- | --- | --- | --- | --- | --- | --- | --- |
| **8** | **Was follow-up complete and if not, were differences between groups in terms of their follow-up adequately described and analyzed?** | | | | | | The study involved over 400 students, with data collected for each event. There is no mention of missing questionnaires or dropout. The completeness of both survey and log data suggests comprehensive follow-up | |  | | | | | | |
|  | **Outcome 1** | | | | | | Usage Statistics | | **Yes** | **No** | | **Unclear** | | **N/A** | |
|  |  | Result 1 | | | | | Table 2 | |  |  |  | | |  | |
|  |  | Result 3 | | | | |  | |  |  |  | | |  | |
|  | **Outcome 2** | | | | | | Student questionnaire | | **Yes** | **No** | **Unclear** | | | **N/A** | |
|  |  | Result 1 | | | | | Table 3 | |  |  |  | | |  | |
|  |  | Result 2 | | | | |  | |  |  |  | | |  | |
|  |  | Result 3 | | | | |  | |  |  |  | | |  | |
|  |  |  | | | | | | |  |  |  | | |  | |
|  | **Statistical Conclusion Validity** | | | | | | | |  |  |  | | |  | |
| **9** | **Was appropriate statistical analysis used?** | | | | | | | The study reports in-table means and standard deviations (e.g., overall positive opinions: M = 4.4, SD = 0.7; usability—M = 4.4, SD = 0.8), reflecting students’ perceptions.  System log data provide descriptive metrics on interaction intensity and scalability (e.g., maximum simultaneous interactions per minute).  While statistical inferential analysis is limited, these methods appropriately support the study’s aims—illustrating usability, engagement, and technical capacity for scalability |  | | | | | | |
|  | **Outcome 1** | | |  | | | | Usage Statistics | **Yes** | **No** | **Unclear** | | | **N/A** | |
|  |  | Result 1 | | | | | | Table 2 |  |  |  | | |  | |
|  |  | Result 3 | | | | | |  |  |  |  | | |  | |
|  | **Outcome 2** | | |  | | | | Student questionnaire | **Yes** | **No** | **Unclear** | | | **N/A** | |
|  |  | Result 1 | | | | | | Table 3 |  |  |  | | |  | |
|  |  | Result 3 | | | | | |  |  |  | | |  | |  |
|  | | | | | | | | | | | | | | | |
| **Overall appraisal:** | | | **Include:** | | **Exclude:** | **Seek Further Info:** | | | | | | | | | |
| **Comments:** Escapp is introduced and validated via three empirical case studies, showing that a specialized platform can effectively support large-scale and remote educational escape rooms. The intervention stacks clearly against outcomes of usability and engagement. While lacking a control condition and baseline measures, the study’s design is fitting for exploratory validation and technology demonstration. Measurements are consistent, follow-up is thorough, and analytical approaches are appropriate for the study’s goals. | | | | | | | | | | | | | | | |

© JBI, 2022. All rights reserved. JBI grants use of these

 tools for research purposes only. All other enquiries

 should be sent to[jbisynthesis@adelaide.edu.au](mailto:jbisynthesis@adelaide.edu.au)

| **RoB Assessor:** SMR | | **Date of Appraisal:** 5/9/25 | | | | **Record Number:** 155 Ref # 41 | | | | |
| --- | --- | --- | --- | --- | --- | --- | --- | --- | --- | --- |
| **Study Author:** Shardlow, M., Sellar, S. & Rousell, D. | | **Study Title:** Collaborative augmentation and simplification of text (CoAST): pedagogical applications of natural language processing in digital learning environments | | | | **Study Year:** 2022 | | | | |
|  | |  | |  | | | | | |  |
| **Internal Validity** | | | **Choice - Comments/Justification** | | **Yes** | | **No** | **Unclear** | **N/A** |  |
| **Bias related to temporal precedence** | | | | | | | | | |  |
| **1** | **Is it clear in the study what is the “cause” and what is the “effect” (i.e. there is no confusion about which variable comes first)?** | | Cause (intervention): The introduction of the CoAST system, a web-based tool leveraging NLP to identify difficult words in academic texts and allowing lecturers to annotate/simplify collaboratively.  Effect (outcomes): Improvements in students' comprehension and recall of theoretical material, as well as enhanced engagement and mediated pedagogical relationships.  The study clearly positions the CoAST environment as preceding and enabling the observed outcomes. | |  | |  |  |  |  |
| **Bias related to selection and allocation** | | | | | | | | | |  |
| **2** | **Was there a control group?** | | A quasi-experimental design involving two cohorts: one using CoAST with annotations, and another with the system but without annotations (i.e., baseline) | |  | |  |  |  |  |
| **Bias related to confounding factors** | | | | | | | | | |  |
| **3** | **Were participants included in any comparisons similar?** | | Both groups comprised undergraduate students: 23 in Education Studies and 23 in Digital & Technology Solutions. Serving similar academic levels/context, they were suitable for comparison. | |  | |  |  |  |  |
| **Bias related to administration of intervention/exposure** | | | | | | | | | |  |
| **4** | **Were the participants included in any comparisons receiving similar treatment/care, other than the exposure or intervention of interest?** | | Both cohorts interacted with the CoAST interface and text content under similar learning conditions, with the only variation being the presence or absence of lecturer annotations. | |  | |  |  |  |  |

| **Bias related to assessment, detection and measurement of the outcome** | | | | | | |
| --- | --- | --- | --- | --- | --- | --- |
| **5** | **Were there multiple measurements of the outcome, both pre and post the intervention/exposure?** | The study reports pretest and posttest measures, showing that students’ comprehension or recall improved, with annotation users experiencing an average increase of 12 percentage points. | **Yes** | **No** | **Unclear** | **N/A** |
|  | **Outcome 1** | Agreement between two teachers |  |  |  |  |
|  | **Outcome 2** | Positive improvement in performance between control and experimental |  |  |  |  |
|  | **Outcome 3** | Word Frequency |  |  |  |  |
|  |  |  |  |  |  |  |
| **6** | **Were the outcomes of participants included in any comparisons measured in the same way?** |  | **Yes** | **No** | **Unclear** | **N/A** |
|  | **Outcome 1** | Agreement between two teachers |  |  |  |  |
|  | **Outcome 2** | Positive improvement in performance between control and experimental |  |  |  |  |
|  | **Outcome 3** | Word Frequency |  |  |  |  |
|  |  |  |  |  |  |  |
| **7** | **Were outcomes measured in a reliable way?** |  | **Yes** | **No** | **Unclear** | **N/A** |
|  | **Outcome 1** | Agreement between two teachers |  |  |  |  |
|  | **Outcome 2** | Positive improvement in performance between control and experimental |  |  |  |  |
|  | **Outcome 3** | Word Frequency |  |  |  |  |

| **Bias related to participant retention** | | | | | | | | | | | | | |
| --- | --- | --- | --- | --- | --- | --- | --- | --- | --- | --- | --- | --- | --- |
| **8** | **Was follow-up complete and if not, were differences between groups in terms of their follow-up adequately described and analyzed?** | | | | | |  | |  | | | | |
|  | **Outcome 1** | | | | | | Agreement between two teachers | | **Yes** | **No** | | **Unclear** | **N/A** |
|  |  | Result 1 | | | | | Table 2 | |  |  |  | |  |
|  | **Outcome 2** | | | | | | Positive improvement in performance between control and experimental | | **Yes** | **No** | **Unclear** | | **N/A** |
|  |  | Result 1 | | | | | Table 3 | |  |  |  | |  |
|  |  | Result 2 | | | | |  | |  |  |  | |  |
|  | **Outcome 3** | | | | | | Word Frequency | | **Yes** | **No** | **Unclear** | | **N/A** |
|  |  | Result 1 | | | | | Table 4 | |  |  |  | |  |
|  |  |  | | | | | | |  |  |  | |  |
|  | **Statistical Conclusion Validity** | | | | | | | |  |  |  | |  |
| **9** | **Was appropriate statistical analysis used?** | | | | | | |  |  | | | | |
|  | **Outcome 1** | | |  | | | | Agreement between two teachers | **Yes** | **No** | **Unclear** | | **N/A** |
|  |  | Result 1 | | | | | | Table 2 |  |  |  | |  |
|  | **Outcome 2** | | |  | | | | Positive improvement in performance between control and experimental | **Yes** | **No** | **Unclear** | | **N/A** |
|  |  | Result 1 | | | | | | Table 3 |  |  |  | |  |
|  | **Outcome 3** | | |  | | | | Word Frequency | **Yes** | **No** | **Unclear** | | **N/A** |
|  |  | Result 1 | | | | | | Table 4 |  |  |  | |  |
|  | | | | | | | | | | | | | |
| **Overall appraisal:** | | | **Include:** | | **Exclude:** | **Seek Further Info:** | | | | | | | |
| **Comments:** The study clearly defines cause (human-augmented NLP annotations) and effect (student comprehension, engagement). It uses comparable learner groups, consistent assessment methods, and includes baseline comparisons. | | | | | | | | | | | | | |

JBI Critical Appraisal Checklist for
Qualitative Research

Reviewer: S. Rutherford Date: 26/08/2025

Author: Shardlow, M., Sellar, S. & Rousell, D. (2022) Collaborative augmentation and simplification of text (CoAST): pedagogical applications of natural language processing in digital learning environments, *Learn Environ Res*, **25**, 399-421.

Year: 2022 Record Number: 155 Ref #41

|  | Yes | No | Unclear | Not applicable |
| --- | --- | --- | --- | --- |
| 1. Is there congruity between the stated philosophical perspective and the research methodology? | x | □ | □ | □ |
| 1. Is there congruity between the research methodology and the research question or objectives? | x | □ | □ | □ |
| 1. Is there congruity between the research methodology and the methods used to collect data? | x | □ | □ | □ |
| 1. Is there congruity between the research methodology and the representation and analysis of data? | x | □ | □ | □ |
| 1. Is there congruity between the research methodology and the interpretation of results? | x | □ | □ | □ |
| 1. Is there a statement locating the researcher culturally or theoretically? | x | □ | □ | □ |
| 1. Is the influence of the researcher on the research, and vice- versa, addressed? | □ | x | □ | □ |
| 1. Are participants, and their voices, adequately represented? | x | □ | □ | □ |
| 1. Is the research ethical according to current criteria or, for recent studies, and is there evidence of ethical approval by an appropriate body? | x | □ | □ | □ |
| 1. Do the conclusions drawn in the research report flow from the analysis, or interpretation, of the data? | x | □ | □ | □ |

Overall appraisal: Include x Exclude □ Seek further info □

Comments (Including reason for exclusion): The study demonstrates strong coherence and an appropriate theoretical grounding. The integration of NLP technology with pedagogical processes, philosophical framing, and mixed-methods evaluation lend it robustness. The work appeared to have been carried out according to ethical guidelines, but no ethics statement was included in the text.

| **RoB Assessor:** SMR | | **Date of Appraisal:** 5/9/25 | | | | **Record Number:** 223 Ref # 45 | | | |  |
| --- | --- | --- | --- | --- | --- | --- | --- | --- | --- | --- |
| **Study Author:** Ibtissam, C., Elmostafa, T., Radid, M. & Yazza, Y. | | **Study Title:** Learning Electrolysis with Podcasting in the Higher Education: From Implementation to Results | | | | **Study Year:** 2019 | | | |  |
|  | |  | |  | | | | | | |
| **Internal Validity** | | | **Choice - Comments/Justification** | | **Yes** | | **No** | **Unclear** | **N/A** | |
| **Bias related to temporal precedence** | | | | | | | | | | |
| **1** | **Is it clear in the study what is the “cause” and what is the “effect” (i.e. there is no confusion about which variable comes first)?** | | Cause (intervention): Implementation of podcasting (video capsules) explaining electrolysis chemistry, delivered via smartphones to students.  Effect (outcomes): Enhanced motivation, self-learning, and informal learning practices among students; and positive shifts in teaching approach among instructors.  This causal relationship is explicit and intentional in the study design | |  | |  |  |  | |
| **Bias related to selection and allocation** | | | | | | | | | | |
| **2** | **Was there a control group?** | | The study employs a descriptive evaluation without a control group. It relies on a survey of 90 students and interviews with 30 students and teachers to capture perceptions and experiences with podcasting | |  | |  |  |  | |
| **Bias related to confounding factors** | | | | | | | | | | |
| **3** | **Were participants included in any comparisons similar?** | | Participants are 90 second-year undergraduates in Materials Science Chemistry at the same university, all engaged in the same module, ensuring homogeneity for evaluation purposes | |  | |  |  |  | |
| **Bias related to administration of intervention/exposure** | | | | | | | | | | |
| **4** | **Were the participants included in any comparisons receiving similar treatment/care, other than the exposure or intervention of interest?** | | All participants received the same podcast content and educational scenario. Interviews were conducted post-lecture in a non-exam period to avoid evaluative pressure. Teaching context and instructional materials were consistent across participants | |  | |  |  |  | |

| **Bias related to assessment, detection and measurement of the outcome** | | | | | | |
| --- | --- | --- | --- | --- | --- | --- |
| **5** | **Were there multiple measurements of the outcome, both pre and post the intervention/exposure?** | The study captures post-intervention perceptions only—through questionnaires and interviews. There is no pre-intervention baseline for comparison | **Yes** | **No** | **Unclear** | **N/A** |
|  | **Outcome 1** | Podcasts usage |  |  |  |  |
|  |  |  |  |  |  |  |
| **6** | **Were the outcomes of participants included in any comparisons measured in the same way?** |  | **Yes** | **No** | **Unclear** | **N/A** |
|  | **Outcome 1** | Podcasts usage |  |  |  |  |
|  |  |  |  |  |  |  |
| **7** | **Were outcomes measured in a reliable way?** |  | **Yes** | **No** | **Unclear** | **N/A** |
|  | **Outcome 1** | Podcasts usage |  |  |  |  |

| **Bias related to participant retention** | | | | | | | | | | | | | |
| --- | --- | --- | --- | --- | --- | --- | --- | --- | --- | --- | --- | --- | --- |
| **8** | **Was follow-up complete and if not, were differences between groups in terms of their follow-up adequately described and analyzed?** | | | | | |  | |  | | | | |
|  | **Outcome 1** | | | | | | Podcasts usage | | **Yes** | **No** | | **Unclear** | **N/A** |
|  |  | Result 1 | | | | | Table 1 | |  |  |  | |  |
|  |  |  | | | | | | |  |  |  | |  |
|  | **Statistical Conclusion Validity** | | | | | | | |  |  |  | |  |
| **9** | **Was appropriate statistical analysis used?** | | | | | | |  |  | | | | |
|  | **Outcome 1** | | |  | | | | Podcasts usage | **Yes** | **No** | **Unclear** | | **N/A** |
|  |  | Result 1 | | | | | | Table 1 |  |  |  | |  |
|  | | | | | | | | | | | | | |
| **Overall appraisal:** | | | **Include:** | | **Exclude:** | **Seek Further Info:** | | | | | | | |
| **Comments:** The study identifies the intervention and intended outcomes, uses a homogeneous student sample, and applies consistent measurement methods. While it lacks pre-intervention data and statistical rigor, the approach is suitable for preliminary insights into feasibility, motivation, and instructional potential. The qualitative and perception-focused methods align with the study’s goals of exploring acceptance and practice change. | | | | | | | | | | | | | |

© JBI, 2022. All rights reserved. JBI grants use of these

 tools for research purposes only. All other enquiries

 should be sent to[jbisynthesis@adelaide.edu.au](mailto:jbisynthesis@adelaide.edu.au)

JBI Critical Appraisal Checklist for
Qualitative Research

Reviewer: S. Rutherford Date: 29/08/2025

Author: Mayo-Cubero, M. (2021) Teaching Innovation Experience for COVID-19 Times: A Case Study on Blended Learning of Television Journalism Courses with Moodle, *Asia Pac Media* *Educ*, **31**, 178-194.

Year: 2021 Record Number: 167 Ref #47

|  | Yes | No | Unclear | Not applicable |
| --- | --- | --- | --- | --- |
| 1. Is there congruity between the stated philosophical perspective and the research methodology? | x | □ | □ | □ |
| 1. Is there congruity between the research methodology and the research question or objectives? | x | □ | □ | □ |
| 1. Is there congruity between the research methodology and the methods used to collect data? | x | □ | □ | □ |
| 1. Is there congruity between the research methodology and the representation and analysis of data? | x | □ | □ | □ |
| 1. Is there congruity between the research methodology and the interpretation of results? | x | □ | □ | □ |
| 1. Is there a statement locating the researcher culturally or theoretically? | □ | x | □ | □ |
| 1. Is the influence of the researcher on the research, and vice- versa, addressed? | □ | x | □ | □ |
| 1. Are participants, and their voices, adequately represented? | x | □ | □ | □ |
| 1. Is the research ethical according to current criteria or, for recent studies, and is there evidence of ethical approval by an appropriate body? | x | □ | □ | □ |
| 1. Do the conclusions drawn in the research report flow from the analysis, or interpretation, of the data? | x | □ | □ | □ |

Overall appraisal: Include x Exclude □ Seek further info □

Comments (Including reason for exclusion): While the philosophical approach is declared, the article does not include reflexive commentary on the researcher’s cultural or positional standpoint. The study does not discuss reflexivity or how the researcher’s presence or perspectives may have influenced data collection or interpretation. Neither of these make the article unfit for use.

| **RoB Assessor:** SMR | | **Date of Appraisal:** 28/8/25 | | **Record Number:** 194 Ref # 48 | | | | |
| --- | --- | --- | --- | --- | --- | --- | --- | --- |
| **Study Author:** Von Sass, P. F., Scheckenbach, K., Wagenmann, M., Klenzner, T., Schipper, J. & Chaker, A. | | **Study Title:** Taking a fresh look at the skull base in otorhinolaryngology with web-based simulation: Student's Interactive Skull-Base Trainer (SISTer) | | **Study Year:** 2019 | | | | |
|  | |  | |  | | | | |
| **Internal Validity** | | | **Choice - Comments/Justification** | | **Yes** | **No** | **Unclear** | **N/A** |
| **Bias related to temporal precedence** | | | | | | | | |
| **1** | **Is it clear in the study what is the “cause” and what is the “effect” (i.e. there is no confusion about which variable comes first)?** | | Cause (intervention): Introduction of SISTer, a self-assessed, web based, adaptive e learning simulation tool for teaching skull-base anatomy.  Effect (outcomes): Medical students’ self-reported enhancement of analytical and clinical problem-solving skills, as well as high levels of acceptance and perceived personal value of the tool. | |  |  |  |  |
| **Bias related to selection and allocation** | | | | | | | | |
| **2** | **Was there a control group?** | | The authors explicitly state that “the design of our investigation did not allow comparison between different groups”. This means all results come from the intervention group only, with no parallel comparison against students not exposed to SISTer. | |  |  |  |  |
| **Bias related to confounding factors** | | | | | | | | |
| **3** | **Were participants included in any comparisons similar?** | | Because there was no control group, there were no direct comparisons between intervention and non-intervention groups. The only comparisons were within the intervention group (e.g., across different semesters, or between usage/acceptance rates). Since all were advanced medical students from the same university cohort, the participants were broadly similar, but no formal matching was required. | |  |  |  |  |
| **Bias related to administration of intervention/exposure** | | | | | | | | |
| **4** | **Were the participants included in any comparisons receiving similar treatment/care, other than the exposure or intervention of interest?** | | All participants were medical students enrolled in the same otolaryngology curriculum, which included standard lectures, seminars, tutorials, and bedside teaching | |  |  |  |  |

| **Bias related to assessment, detection and measurement of the outcome** | | | | | | |
| --- | --- | --- | --- | --- | --- | --- |
| **5** | **Were there multiple measurements of the outcome, both pre and post the intervention/exposure?** |  | **Yes** | **No** | **Unclear** | **N/A** |
|  | **Outcome 1** | Providing Incentive for User Demand |  |  |  |  |
|  | **Outcome 2** | Good Acceptance of e-Learning on the Skull Base |  |  |  |  |
|  | **Outcome 3** | Enabling Students to Identify Learning Deficits by e-Learning |  |  |  |  |
|  | **Outcome 7** |  |  |  |  |  |
|  |  |  |  |  |  |  |
| **6** | **Were the outcomes of participants included in any comparisons measured in the same way?** |  | **Yes** | **No** | **Unclear** | **N/A** |
|  | **Outcome 1** | Providing Incentive for User Demand |  |  |  |  |
|  | **Outcome 2** | Good Acceptance of e-Learning on the Skull Base |  |  |  |  |
|  | **Outcome 3** | Enabling Students to Identify Learning Deficits by e-Learning |  |  |  |  |
|  | **Outcome 7** |  |  |  |  |  |
|  |  |  |  |  |  |  |
| **7** | **Were outcomes measured in a reliable way?** |  | **Yes** | **No** | **Unclear** | **N/A** |
|  | **Outcome 1** | Providing Incentive for User Demand |  |  |  |  |
|  | **Outcome 2** | Good Acceptance of e-Learning on the Skull Base |  |  |  |  |
|  | **Outcome 3** | Enabling Students to Identify Learning Deficits by e-Learning |  |  |  |  |
|  | **Outcome 7** |  |  |  |  |  |

| **Bias related to participant retention** | | | | | | | | | | | | | | | |
| --- | --- | --- | --- | --- | --- | --- | --- | --- | --- | --- | --- | --- | --- | --- | --- |
| **8** | **Was follow-up complete and if not, were differences between groups in terms of their follow-up adequately described and analyzed?** | | | | | |  | |  | | | | | | |
|  | **Outcome 1** | | | | | | Providing Incentive for User Demand | | **Yes** | **No** | | **Unclear** | | **N/A** | |
|  |  | Result 1 | | | | | Figure 2 | |  |  |  | | |  | |
|  |  | Result 2 | | | | | Figure 3 | |  |  |  | | |  | |
|  |  | Result 3 | | | | |  | |  |  |  | | |  | |
|  | **Outcome 2** | | | | | | Good Acceptance of e-Learning on the Skull Base | | **Yes** | **No** | **Unclear** | | | **N/A** | |
|  |  | Result 1 | | | | | Figure 4 | |  |  |  | | |  | |
|  |  | Result 2 | | | | |  | |  |  |  | | |  | |
|  |  | Result 3 | | | | |  | |  |  |  | | |  | |
|  | **Outcome 3** | | | | | | Enabling Students to Identify Learning Deficits by e-Learning | | **Yes** | **No** | **Unclear** | | | **N/A** | |
|  |  | Result 1 | | | | | Figure 5 | |  |  |  | | |  | |
|  |  | Result 3 | | | | |  | |  |  |  | | |  | |
|  |  |  | | | | | | |  |  |  | | |  | |
|  | **Statistical Conclusion Validity** | | | | | | | |  |  |  | | |  | |
| **9** | **Was appropriate statistical analysis used?** | | | | | | |  |  | | | | | | |
|  | **Outcome 1** | | |  | | | | Providing Incentive for User Demand | **Yes** | **No** | **Unclear** | | | **N/A** | |
|  |  | Result 1 | | | | | | Figure 2 |  |  |  | | |  | |
|  |  | Result 2 | | | | | | Figure 3 |  |  |  | | |  | |
|  |  | Result 3 | | | | | |  |  |  |  | | |  | |
|  | **Outcome 2** | | |  | | | | Good Acceptance of e-Learning on the Skull Base | **Yes** | **No** | **Unclear** | | | **N/A** | |
|  |  | Result 1 | | | | | | Figure 4 |  |  |  | | |  | |
|  |  | Result 3 | | | | | |  |  |  |  | | |  | |
|  | **Outcome 3** | | |  | | | | Enabling Students to Identify Learning Deficits by e-Learning | **Yes** | **No** | **Unclear** | | | **N/A** | |
|  |  | Result 1 | | | | | | Figure 5 |  |  |  | | |  | |
|  |  | Result 3 | | | | | |  |  |  | | |  | |  |
|  | | | | | | | | | | | | | | | |
| **Overall appraisal:** | | | **Include:** | | **Exclude:** | **Seek Further Info:** | | | | | | | | | |
| **Comments:** | | | | | | | | | | | | | | | |

© JBI, 2022. All rights reserved. JBI grants use of these

 tools for research purposes only. All other enquiries

 should be sent to[jbisynthesis@adelaide.edu.au](mailto:jbisynthesis@adelaide.edu.au)

| **RoB Assessor:** SMR | | **Date of Appraisal:** 29/8/25 | | **Record Number:** 192 Ref # 50 | | | | |
| --- | --- | --- | --- | --- | --- | --- | --- | --- |
| **Study Author:** Zhang, H., Song, W. & Burston, J. | | **Study Title:** Reexamining the effectiveness of vocabulary learning via mobile phones | | **Study Year:** 2011 | | | | |
|  | |  | |  | | | | |
| **Internal Validity** | | | **Choice - Comments/Justification** | | **Yes** | **No** | **Unclear** | **N/A** |
| **Bias related to temporal precedence** | | | | | | | | |
| **1** | **Is it clear in the study what is the “cause” and what is the “effect” (i.e. there is no confusion about which variable comes first)?** | | Cause (intervention): Delivery of vocabulary via mobile phone SMS (experimental group), compared to traditional paper-based vocabulary lists (control group).  Effect (outcomes): Differences in immediate and delayed vocabulary acquisition, measured via posttest and delayed posttest scores. The study explicitly tests whether mobile-based vocabulary exposure leads to different learning outcomes than traditional methods | |  |  |  |  |
| **Bias related to selection and allocation** | | | | | | | | |
| **2** | **Was there a control group?** | | The study used two intact sophomore classes at a Chinese university: one received vocabulary via SMS (experimental group); the other used paper materials (control group) | |  |  |  |  |
| **Bias related to confounding factors** | | | | | | | | |
| **3** | **Were participants included in any comparisons similar?** | | Participants were sophomore students from the same institution and course, grouped intact. A pretest confirmed no significant difference in baseline vocabulary knowledge between groups (SMS group vs paper group: no significant difference in pretest means) | |  |  |  |  |
| **Bias related to administration of intervention/exposure** | | | | | | | | |
| **4** | **Were the participants included in any comparisons receiving similar treatment/care, other than the exposure or intervention of interest?** | | Apart from the method of vocabulary delivery, both groups engaged in self-regulated learning, with the same vocabulary lists and testing procedures. Tests were administered under similar conditions across groups. | |  |  |  |  |

| **Bias related to assessment, detection and measurement of the outcome** | | | | | | |
| --- | --- | --- | --- | --- | --- | --- |
| **5** | **Were there multiple measurements of the outcome, both pre and post the intervention/exposure?** | Pre-test measured baseline vocabulary knowledge.  Post-test assessed immediate learning.  Delayed post-test checked retention after a period. | **Yes** | **No** | **Unclear** | **N/A** |
|  | **Outcome 1** |  |  |  |  |  |
|  | **Outcome 7** |  |  |  |  |  |
|  |  |  |  |  |  |  |
| **6** | **Were the outcomes of participants included in any comparisons measured in the same way?** | The same tests were used for pretest, posttest, and delayed test across both groups, ensuring consistency in measurement | **Yes** | **No** | **Unclear** | **N/A** |
|  | **Outcome 1** | Level of vocabulary knowledge before and after treatments |  |  |  |  |
|  | **Outcome 7** |  |  |  |  |  |
|  |  |  |  |  |  |  |
| **7** | **Were outcomes measured in a reliable way?** |  | **Yes** | **No** | **Unclear** | **N/A** |
|  | **Outcome 1** | Level of vocabulary knowledge before and after treatments |  |  |  |  |
|  | **Outcome 7** |  |  |  |  |  |

| **Bias related to participant retention** | | | | | | | | | | | | | | | |
| --- | --- | --- | --- | --- | --- | --- | --- | --- | --- | --- | --- | --- | --- | --- | --- |
| **8** | **Was follow-up complete and if not, were differences between groups in terms of their follow-up adequately described and analyzed?** | | | | | |  | |  | | | | | | |
|  | **Outcome 1** | | | | | | Level of vocabulary knowledge before and after treatments | | **Yes** | **No** | | **Unclear** | | **N/A** | |
|  |  | Result 1 | | | | | Table 1 | |  |  |  | | |  | |
|  |  | Result 3 | | | | |  | |  |  |  | | |  | |
|  |  |  | | | | | | |  |  |  | | |  | |
|  | **Statistical Conclusion Validity** | | | | | | | |  |  |  | | |  | |
| **9** | **Was appropriate statistical analysis used?** | | | | | | |  |  | | | | | | |
|  | **Outcome 1** | | |  | | | | Level of vocabulary knowledge before and after treatments | **Yes** | **No** | **Unclear** | | | **N/A** | |
|  |  | Result 1 | | | | | | Table 1 |  |  |  | | |  | |
|  |  | Result 3 | | | | | |  |  |  | | |  | |  |
|  | | | | | | | | | | | | | | | |
| **Overall appraisal:** | | | **Include:** | | **Exclude:** | **Seek Further Info:** | | | | | | | | | |
| **Comments:** The immediate posttest advantage of SMS-based learning is clearly shown, while the lack of delayed test differences indicates ephemeral gains and suggests the value of a blended or sustained practice approach. Statistical treatment is appropriate, and study design solidly supports the findings. | | | | | | | | | | | | | | | |

© JBI, 2022. All rights reserved. JBI grants use of these

 tools for research purposes only. All other enquiries

 should be sent to[jbisynthesis@adelaide.edu.au](mailto:jbisynthesis@adelaide.edu.au)

| **RoB Assessor:** SMR | | **Date of Appraisal:** 7/9/25 | | **Record Number:** 157 Ref # 56 | | | | |
| --- | --- | --- | --- | --- | --- | --- | --- | --- |
| **Study Author:** Alwadei, A. H., Tekian, A. S., Brown, B. P., Alwadei, F. H., Park, Y. S., Alwadei, S. H. & Harris, I. B. | | **Study Title:** Effectiveness of an adaptive eLearning intervention on dental students’ learning in comparison to traditional instruction | | **Study Year:** 2020 | | | | |
|  | |  | |  | | | | |
| **Internal Validity** | | | **Choice - Comments/Justification** | | **Yes** | **No** | **Unclear** | **N/A** |
| **Bias related to temporal precedence** | | | | | | | | |
| **1** | **Is it clear in the study what is the “cause” and what is the “effect” (i.e. there is no confusion about which variable comes first)?** | | Cause (intervention): Use of an Adaptive Learning Platform (ALP) by dental students in a blended learning format, delivered either formatively (practice before instruction) or summatively (primary learning method).  Effect (outcome): Improved student performance, measured via final exam scores on a dental review preparatory course. The study clearly tests how the learning modality (ALP vs traditional face-to-face) drives changes in performance. | |  |  |  |  |
| **Bias related to selection and allocation** | | | | | | | | |
| **2** | **Was there a control group?** | | The study includes a traditional instruction group (face-to-face, no ALP), serving as the control, alongside two intervention groups (formative ALP and summative ALP) in the comparison. | |  |  |  |  |
| **Bias related to confounding factors** | | | | | | | | |
| **3** | **Were participants included in any comparisons similar?** | | Participants were dental students in the same preparatory course across several academic years (2013–2018). Pre-test scores were used to control for baseline differences, confirming group comparability before the intervention | |  |  |  |  |
| **Bias related to administration of intervention/exposure** | | | | | | | | |
| **4** | **Were the participants included in any comparisons receiving similar treatment/care, other than the exposure or intervention of interest?** | | Apart from the mode of instruction (ALP use vs traditional), all participants engaged in the same course, content, and final exam, ensuring standard learning conditions across groups | |  |  |  |  |

| **Bias related to assessment, detection and measurement of the outcome** | | | | | | |
| --- | --- | --- | --- | --- | --- | --- |
| **5** | **Were there multiple measurements of the outcome, both pre and post the intervention/exposure?** | The study used pre-test scores to control baseline equivalence and post-test (final exam) as outcome measures. Although not multiple post-tests, this allows within-group improvement and between-group comparison. | **Yes** | **No** | **Unclear** | **N/A** |
|  | **Outcome 1** | Learning improvement within  groups based on different course modalities |  |  |  |  |
|  | **Outcome 2** | Differences in learning outcome  between formative and summative ALP  groups compared with traditional group |  |  |  |  |
|  | **Outcome 3** | Differences in learning outcome  between summative and formative ALP  groups |  |  |  |  |
|  |  |  |  |  |  |  |
| **6** | **Were the outcomes of participants included in any comparisons measured in the same way?** | All students’ learning outcomes were assessed through the same final exam, and pre-test results were standardised across groups to allow valid comparisons. | **Yes** | **No** | **Unclear** | **N/A** |
|  | **Outcome 1** | Learning improvement within  groups based on different course modalities |  |  |  |  |
|  | **Outcome 2** | Differences in learning outcome  between formative and summative ALP  groups compared with traditional group |  |  |  |  |
|  | **Outcome 3** | Differences in learning outcome  between summative and formative ALP  groups |  |  |  |  |
|  |  |  |  |  |  |  |
| **7** | **Were outcomes measured in a reliable way?** |  | **Yes** | **No** | **Unclear** | **N/A** |
|  | **Outcome 1** | Learning improvement within  groups based on different course modalities |  |  |  |  |
|  | **Outcome 2** | Differences in learning outcome  between formative and summative ALP  groups compared with traditional group |  |  |  |  |
|  | **Outcome 3** | Differences in learning outcome  between summative and formative ALP  groups |  |  |  |  |

| **Bias related to participant retention** | | | | | | | | | | | | | |
| --- | --- | --- | --- | --- | --- | --- | --- | --- | --- | --- | --- | --- | --- |
| **8** | **Was follow-up complete and if not, were differences between groups in terms of their follow-up adequately described and analyzed?** | | | | | |  | |  | | | | |
|  | **Outcome 1** | | | | | | Learning improvement within  groups based on different course modalities | | **Yes** | **No** | | **Unclear** | **N/A** |
|  |  | Result 1 | | | | | Table 2 | |  |  |  | |  |
|  | **Outcome 2** | | | | | | Differences in learning outcome  between formative and summative ALP  groups compared with traditional group | | **Yes** | **No** | **Unclear** | | **N/A** |
|  |  | Result 1 | | | | | Table 3 | |  |  |  | |  |
|  |  | Result 2 | | | | | Table 4 | |  |  |  | |  |
|  | **Outcome 3** | | | | | | Differences in learning outcome  between summative and formative ALP  groups | | **Yes** | **No** | **Unclear** | | **N/A** |
|  |  | Result 1 | | | | | Table 5 | |  |  |  | |  |
|  |  |  | | | | | | |  |  |  | |  |
|  | **Statistical Conclusion Validity** | | | | | | | |  |  |  | |  |
| **9** | **Was appropriate statistical analysis used?** | | | | | | |  |  | | | | |
|  | **Outcome 1** | | |  | | | | Learning improvement within  groups based on different course modalities | **Yes** | **No** | **Unclear** | | **N/A** |
|  |  | Result 1 | | | | | | Table 2 |  |  |  | |  |
|  | **Outcome 2** | | |  | | | | Differences in learning outcome  between formative and summative ALP  groups compared with traditional group | **Yes** | **No** | **Unclear** | | **N/A** |
|  |  | Result 1 | | | | | | Table 3 |  |  |  | |  |
|  |  | Result 2 | | | | | | Table 4 |  |  |  | |  |
|  | **Outcome 3** | | |  | | | | Differences in learning outcome  between summative and formative ALP  groups | **Yes** | **No** | **Unclear** | | **N/A** |
|  |  | Result 1 | | | | | | Table 5 |  |  |  | |  |
|  | | | | | | | | | | | | | |
| **Overall appraisal:** | | | **Include:** | | **Exclude:** | **Seek Further Info:** | | | | | | | |
| **Comments:** This study offers strong evidence that using an adaptive eLearning platform—particularly in a summative format—is more effective than traditional instruction or formative use alone for dental students preparing for exams. The research design includes a control group and uses rigorous statistical controls to account for baseline differences. It is methodologically sound and demonstrates the potential of ALP in professional education settings. | | | | | | | | | | | | | |

© JBI, 2022. All rights reserved. JBI grants use of these

 tools for research purposes only. All other enquiries

 should be sent to[jbisynthesis@adelaide.edu.au](mailto:jbisynthesis@adelaide.edu.au)

JBI Critical Appraisal Checklist for
Qualitative Research

Reviewer: S. Rutherford Date: 26/08/2025

Author: Choo, W. S. (2021) Student perspectives of various learning approaches used in an undergraduate food science and technology subject*, J Food Sci Educ*, **20**, 146-154.

Year: 2021 Record Number: 156 Ref #57

|  | Yes | No | Unclear | Not applicable |
| --- | --- | --- | --- | --- |
| 1. Is there congruity between the stated philosophical perspective and the research methodology? | x | □ | □ | □ |
| 1. Is there congruity between the research methodology and the research question or objectives? | x | □ | □ | □ |
| 1. Is there congruity between the research methodology and the methods used to collect data? | x | □ | □ | □ |
| 1. Is there congruity between the research methodology and the representation and analysis of data? | x | □ | □ | □ |
| 1. Is there congruity between the research methodology and the interpretation of results? | x | □ | □ | □ |
| 1. Is there a statement locating the researcher culturally or theoretically? | □ | x | □ | □ |
| 1. Is the influence of the researcher on the research, and vice- versa, addressed? | □ | x | □ | □ |
| 1. Are participants, and their voices, adequately represented? | x | □ | □ | □ |
| 1. Is the research ethical according to current criteria or, for recent studies, and is there evidence of ethical approval by an appropriate body? | x | □ | □ | □ |
| 1. Do the conclusions drawn in the research report flow from the analysis, or interpretation, of the data? | x | □ | □ | □ |

Overall appraisal: Include x Exclude □ Seek further info □

Comments (Including reason for exclusion): There’s no indication of reflexivity or the researchers positioning themselves culturally or theoretically. The study does not discuss reflexivity regarding potential researcher influence. These do not make the study unviable, however. General conclusions—such as the value of hands-on labs, the role of polling tools, and emphasising alignment of learning approach with intended outcomes—are consistent with the findings reported.

| **RoB Assessor:** SMR | | **Date of Appraisal:** 7/9/25 | | **Record Number:** 165 Ref # 58 | | | | |
| --- | --- | --- | --- | --- | --- | --- | --- | --- |
| **Study Author:** Salas-Rueda, R. A. | | **Study Title:** Impact of the WampServer application in Blended learning considering data science, machine learning, and neural networks | | **Study Year:** 2020 | | | | |
|  | |  | |  | | | | |
| **Internal Validity** | | | **Choice - Comments/Justification** | | **Yes** | **No** | **Unclear** | **N/A** |
| **Bias related to temporal precedence** | | | | | | | | |
| **1** | **Is it clear in the study what is the “cause” and what is the “effect” (i.e. there is no confusion about which variable comes first)?** | | Cause (intervention): Use of WampServer — a free application facilitating local web development using PHP, HTML, SQL, and database integration — in a Blended Learning setting.  Effect (outcomes): Improved assimilation of knowledge and the development of web programming skills among students, demonstrated via predictive modeling.  This causal link is clearly established in the study’s design and articulated | |  |  |  |  |
| **Bias related to selection and allocation** | | | | | | | | |
| **2** | **Was there a control group?** | | The study adopted a one-group design without a non-intervention or comparative control group. All 28 students participated under the same intervention condition | |  |  |  |  |
| **Bias related to confounding factors** | | | | | | | | |
| **3** | **Were participants included in any comparisons similar?** | | The sample comprised 28 students enrolled in a “Development of Applications” course in the 2016 academic year. It appears to be a homogeneous cohort, ensuring internal comparability | |  |  |  |  |
| **Bias related to administration of intervention/exposure** | | | | | | | | |
| **4** | **Were the participants included in any comparisons receiving similar treatment/care, other than the exposure or intervention of interest?** | | All students experienced the same blended learning methodology: in-class discussion and reflection on database concepts, followed by home-based application development using WampServer. The learning context was consistent for all. | |  |  |  |  |

| **Bias related to assessment, detection and measurement of the outcome** | | | | | | |
| --- | --- | --- | --- | --- | --- | --- |
| **5** | **Were there multiple measurements of the outcome, both pre and post the intervention/exposure?** | The study used pre-test scores to control baseline equivalence and post-test (final exam) as outcome measures. Although not multiple post-tests, this allows within-group improvement and between-group comparison. | **Yes** | **No** | **Unclear** | **N/A** |
|  | **Outcome 1** | Assimilation of knowledge |  |  |  |  |
|  | **Outcome 2** | Development of Skills |  |  |  |  |
|  |  |  |  |  |  |  |
| **6** | **Were the outcomes of participants included in any comparisons measured in the same way?** | All students’ learning outcomes were assessed through the same final exam, and pre-test results were standardised across groups to allow valid comparisons. | **Yes** | **No** | **Unclear** | **N/A** |
|  | **Outcome 1** | Assimilation of knowledge |  |  |  |  |
|  | **Outcome 2** | Development of Skills |  |  |  |  |
|  |  |  |  |  |  |  |
| **7** | **Were outcomes measured in a reliable way?** |  | **Yes** | **No** | **Unclear** | **N/A** |
|  | **Outcome 1** | Assimilation of knowledge |  |  |  |  |
|  | **Outcome 2** | Development of Skills |  |  |  |  |

| **Bias related to participant retention** | | | | | | | | | | | | | | | |
| --- | --- | --- | --- | --- | --- | --- | --- | --- | --- | --- | --- | --- | --- | --- | --- |
| **8** | **Was follow-up complete and if not, were differences between groups in terms of their follow-up adequately described and analyzed?** | | | | | |  | |  | | | | | | |
|  | **Outcome 1** | | | | | | Assimilation of knowledge | | **Yes** | **No** | | **Unclear** | | **N/A** | |
|  |  | Result 1 | | | | | Table 3 | |  |  |  | | |  | |
|  |  | Result 2 | | | | | Table 4 | |  |  |  | | |  | |
|  |  | Result 3 | | | | | Table 5 | |  |  |  | | |  | |
|  |  | Result 4 | | | | | Table 6 | |  |  |  | | |  | |
|  |  | Result 5 | | | | | Table 7 | |  |  |  | | |  | |
|  |  | Result 6 | | | | | Table 8 | |  |  |  | | |  | |
|  | **Outcome 2** | | | | | | Development of skills | | **Yes** | **No** | **Unclear** | | | **N/A** | |
|  |  | Result 1 | | | | | Table 9 | |  |  |  | | |  | |
|  |  | Result 2 | | | | | Table 10 | |  |  |  | | |  | |
|  |  | Result 3 | | | | | Table 11 | |  |  |  | | |  | |
|  |  |  | | | | | | |  |  |  | | |  | |
|  | **Statistical Conclusion Validity** | | | | | | | |  |  |  | | |  | |
| **9** | **Was appropriate statistical analysis used?** | | | | | | |  |  | | | | | | |
|  | **Outcome 1** | | |  | | | | Assimilation of knowledge | **Yes** | **No** | **Unclear** | | | **N/A** | |
|  |  | Result 1 | | | | | | Table 3 |  |  |  | | |  | |
|  |  | Result 2 | | | | | | Table 4 |  |  |  | | |  | |
|  |  | Result 3 | | | | | | Table 5 |  |  |  | | |  | |
|  |  | Result 4 | | | | | | Table 6 |  |  |  | | |  | |
|  |  | Result 5 | | | | | | Table 7 |  |  |  | | |  | |
|  |  | Result 6 | | | | | | Table 8 |  |  |  | | |  | |
|  | **Outcome 2** | | |  | | | | Development of skills | **Yes** | **No** | **Unclear** | | | **N/A** | |
|  |  | Result 1 | | | | | | Table 9 |  |  |  | | |  | |
|  |  | Result 2 | | | | | | Table 10 |  |  |  | | |  | |
|  |  | Result 3 | | | | | | Table 11 |  |  | | |  | |  |
|  | | | | | | | | | | | | | | | |
| **Overall appraisal:** | | | **Include:** | | **Exclude:** | **Seek Further Info:** | | | | | | | | | |
| **Comments:** The cause–effect relationship is clear, with uniform treatment across participants and robust modeling techniques used to reveal how WampServer influenced learning outcomes. While the absence of a control group and pre-post design limits causal inference, the use of advanced analytics (e.g., neural network modeling) provides valuable insight into predictors and influencing factors in student learning. | | | | | | | | | | | | | | | |

© JBI, 2022. All rights reserved. JBI grants use of these

 tools for research purposes only. All other enquiries

 should be sent to[jbisynthesis@adelaide.edu.au](mailto:jbisynthesis@adelaide.edu.au)

| **RoB Assessor:** SMR | | **Date of Appraisal:** 29/8/25 | | **Record Number:** 170 Ref # 59 | | | | |
| --- | --- | --- | --- | --- | --- | --- | --- | --- |
| **Study Author:** Pickering, J. D. & Swinnerton, B. J. | | **Study Title:** Exploring the Dimensions of Medical Student Engagement with Technology-Enhanced Learning Resources and Assessing the Impact on Assessment Outcomes | | **Study Year:** 2019 | | | | |
|  | |  | |  | | | | |
| **Internal Validity** | | | **Choice - Comments/Justification** | | **Yes** | **No** | **Unclear** | **N/A** |
| **Bias related to temporal precedence** | | | | | | | | |
| **1** | **Is it clear in the study what is the “cause” and what is the “effect” (i.e. there is no confusion about which variable comes first)?** | | The study treats engagement with technology-enhanced learning (TEL) resources as the exposure (cause) and assessment outcomes as the effect. There is no confusion about directionality. | |  |  |  |  |
| **Bias related to selection and allocation** | | | | | | | | |
| **2** | **Was there a control group?** | | All students had access to TEL resources. The study was observational and did not include a separate control group. | |  |  |  |  |
| **Bias related to confounding factors** | | | | | | | | |
| **3** | **Were participants included in any comparisons similar?** | | All participants were first-year medical students on the same module, making them comparable in baseline academic context. | |  |  |  |  |
| **Bias related to administration of intervention/exposure** | | | | | | | | |
| **4** | **Were the participants included in any comparisons receiving similar treatment/care, other than the exposure or intervention of interest?** | | All students received the same anatomy teaching and assessments; the only difference was their level of engagement with TEL resources. | |  |  |  |  |

| **Bias related to assessment, detection and measurement of the outcome** | | | | | | |
| --- | --- | --- | --- | --- | --- | --- |
| **5** | **Were there multiple measurements of the outcome, both pre and post the intervention/exposure?** |  | **Yes** | **No** | **Unclear** | **N/A** |
|  | **Outcome 1** | Outcomes were based on a single summative spotter exam (GI/renal) and did not include pre- and post-intervention measures. |  |  |  |  |
|  | **Outcome 2** |  |  |  |  |  |
|  |  |  |  |  |  |  |
| **6** | **Were the outcomes of participants included in any comparisons measured in the same way?** |  | **Yes** | **No** | **Unclear** | **N/A** |
|  | **Outcome 1** | All outcomes were measured using the same standardized anatomy spotter examination format. |  |  |  |  |
|  | **Outcome 2** |  |  |  |  |  |
|  |  |  |  |  |  |  |
| **7** | **Were outcomes measured in a reliable way?** |  | **Yes** | **No** | **Unclear** | **N/A** |
|  | **Outcome 1** | The exam was standardized and objectively marked, suggesting reliable outcome measurement. The engagement survey demonstrated strong internal consistency (Cronbach’s α ≈ 0.86). |  |  |  |  |
|  | **Outcome 2** |  |  |  |  |  |

| **Bias related to participant retention** | | | | | | | | | | | | | |
| --- | --- | --- | --- | --- | --- | --- | --- | --- | --- | --- | --- | --- | --- |
| **8** | **Was follow-up complete and if not, were differences between groups in terms of their follow-up adequately described and analyzed?** | | | | | |  | |  | | | | |
|  | **Outcome 1** | | | | | |  | | **Yes** | **No** | | **Unclear** | **N/A** |
|  |  | Result 1 | | | | | Response rate was high (82.8%). Gender distribution of respondents did not differ significantly from the full cohort, reducing risk of bias from incomplete follow-up. | |  |  |  | |  |
|  |  | Result 2 | | | | |  | |  |  |  | |  |
|  |  | Result 3 | | | | |  | |  |  |  | |  |
|  |  |  | | | | | | |  |  |  | |  |
|  | **Statistical Conclusion Validity** | | | | | | | |  |  |  | |  |
| **9** | **Was appropriate statistical analysis used?** | | | | | | |  |  | | | | |
|  | **Outcome 1** | | |  | | | |  | **Yes** | **No** | **Unclear** | | **N/A** |
|  |  | Result 1 | | | | | | The study used appropriate statistical techniques for the data type, including normality tests, exploratory factor analysis, Cronbach’s α, non-parametric tests (Mann-Whitney, Kruskal-Wallis), and Spearman’s correlations. |  |  |  | |  |
|  |  | Result 2 | | | | | |  |  |  |  | |  |
|  |  | Result 3 | | | | | |  |  |  |  | |  |
|  | | | | | | | | | | | | | |
| **Overall appraisal:** | | | **Include:** | | **Exclude:** | **Seek Further Info:** | | | | | | | |
| **Comments:** | | | | | | | | | | | | | |

© JBI, 2022. All rights reserved. JBI grants use of these

 tools for research purposes only. All other enquiries

 should be sent to[jbisynthesis@adelaide.edu.au](mailto:jbisynthesis@adelaide.edu.au)

JBI Critical Appraisal Checklist for
Qualitative Research

Reviewer: S. Rutherford Date: 29/08/2025

Author: Pickering, J. D. & Swinnerton, B. J. (2019) Exploring the Dimensions of Medical Student Engagement with Technology-Enhanced Learning Resources and Assessing the Impact on Assessment Outcomes, *Anat Sci Educ,* **12**, 117-128.

Year: 2019 Record Number: 170 Ref #59

|  | Yes | No | Unclear | Not applicable |
| --- | --- | --- | --- | --- |
| 1. Is there congruity between the stated philosophical perspective and the research methodology? | □ | x | □ | □ |
| 1. Is there congruity between the research methodology and the research question or objectives? | x | □ | □ | □ |
| 1. Is there congruity between the research methodology and the methods used to collect data? | x | □ | □ | □ |
| 1. Is there congruity between the research methodology and the representation and analysis of data? | x | □ | □ | □ |
| 1. Is there congruity between the research methodology and the interpretation of results? | x | □ | □ | □ |
| 1. Is there a statement locating the researcher culturally or theoretically? | □ | x | □ | □ |
| 1. Is the influence of the researcher on the research, and vice- versa, addressed? | □ | x | □ | □ |
| 1. Are participants, and their voices, adequately represented? | x | □ | □ | □ |
| 1. Is the research ethical according to current criteria or, for recent studies, and is there evidence of ethical approval by an appropriate body? | x | □ | □ | □ |
| 1. Do the conclusions drawn in the research report flow from the analysis, or interpretation, of the data? | x | □ | □ | □ |

Overall appraisal: Include x Exclude □ Seek further info □

Comments: The study has a strong quantitative design that effectively identifies engagement dimensions via factor analysis and statistically examines relationships with usage and outcomes. However, it lacks qualitative depth. The quantitative elements, however, are strong. Overall this merits inclusion.
