# Supplementary material for: Navigating new norms: a systematic review of factors for the development of effective digital tools in higher education
Source: FEBS Open Bio. 2025 Oct 30;16(3):610–27. doi: 10.1002/2211-5463.70151 (PMC12955751; doi:10.1002/2211-5463.70151)
Supplement: Supplementary file 4 — Appendix S3. Claude.ai prompt used to utilise generative artificial intelligence to support and confirm the paper evaluations. [file FEB4-16-610-s001.docx]

**Appendix S3**

**Claude.ai prompt**

Inclusion Criteria:

Primary research studies evaluating specific digital technologies or educational technologies (not conceptual papers)

Studies conducted in a higher education or university setting

Provide evidence of the effectiveness or impact of the digital/educational technologies through quantitative data, qualitative insights, or student perspectives

Examine student perceptions, experiences, or satisfaction with using the digital/educational technologies

Explore factors that influence the effectiveness or support adoption of the digital/educational technologies - must evaluate how and why digital tool is/is not effective

Exclude studies focused only on blended learning, online learning, flipped classroom, or hybrid learning approaches unless they also evaluate a specific digital technology tool - exclude Virtual Learning Environments or Learning management systems as digital tools unless article address how/why they are used as digital tools or evaluates specific tools on the platform.

Does this article fit this inclusion criteria?
